# Discovery of Simple Diacylhydrazine-Functionalized Cinnamic Acid Derivatives as Potential Microtubule Stabilizers

**DOI:** 10.3390/ijms232012365

**Published:** 2022-10-15

**Authors:** Xiang Zhou, Yi-Hong Fu, Ya-Yu Zou, Jiao Meng, Gui-Ping Ou-Yang, Qiang-Sheng Ge, Zhen-Chao Wang

**Affiliations:** 1 State Key Laboratory Breeding Base of Green Pesticide and Agricultural Bioengineering, Key Laboratory of Green Pesticide and Agricultural Bioengineering, Ministry of Education, Center for R & D of Fine Chemicals, Guizhou University, Guiyang 550025, China; 2 Science and Technology College, Hubei University of Arts and Science, Xiangyang 441025, China; 3 College of Pharmacy, Guizhou University, Guiyang 550025, China

**Keywords:** cinnamic acid derivatives, antiproliferative activity, microtubule, tubulin stabilizer

## Abstract

To develop novel microtubule-binding agents for cancer therapy, an array of *N*-cinnamoyl-*N*’-(substituted)acryloyl hydrazide derivatives were facilely synthesized through a two-step process. Initially, the antiproliferative activity of these title compounds was explored against A549, 98 PC-3 and HepG2 cancer cell lines. Notably, compound **I_23_** exhibited the best antiproliferative activity against three cancer lines with IC_50_ values ranging from 3.36 to 5.99 μM and concurrently afforded a lower cytotoxicity towards the NRK-52E cells. Anticancer mechanism investigations suggested that the highly bioactive compound **I_23_** could potentially promote the protofilament assembly of tubulin, thus eventually leading to the stagnation of the G2/M phase cell cycle of HepG2 cells. Moreover, compound **I_23_** also disrupted cancer cell migration and significantly induced HepG2 cells apoptosis in a dosage-dependent manner. Additionally, the in silico analysis indicated that compound **I_23_** exhibited an acceptable pharmacokinetic profile. Overall, these easily prepared *N*-cinnamoyl-*N*’-(substituted)acryloyl hydrazide derivatives could serve as potential microtubule-interacting agents, probably as novel microtubule-stabilizers.

## 1. Introduction

Cancer remains the chief and ever-expanding culprit in human mortality that prompts significant concerns in every country [1]. As the International Agency for Research on Cancer (IARC) reported, this intractable disease had been emerging in approximately 18.1 million new cases and thus leading to 9.6 million cancer deaths in 2018 [2,3]. Except for the use of radiotherapy and surgery, chemotherapy is still an effective approach to treat cancer in view of its fast-acting performance against adversaries, the practical applicability and the available effectiveness, but sometimes certain pharmacologically induced side effects coexist [4,5,6,7]. Therefore, exploring and developing a highly effective therapeutic approach to selectively eliminate cancer cells should be actively pursued.

**Figure 1:**
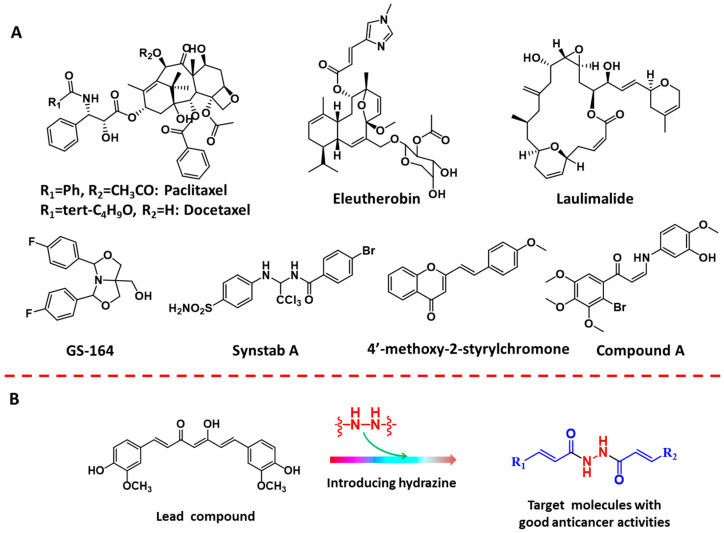
Chemical structures of some microtubule-targeting agents (**A**). Design concepts of target molecules (**B**).

To date, cell cycle modulators or inhibitors, which can arrest uncontrollable tumor growth, are considered as a kind of hopeful antiproliferative agent. Among these modulators, microtubule-interacting agents that target microtubule and subsequently affect multiple cellular processes (e.g., mitosis, cell division, and intracellular transportation) have become a promising group of anticancer agents that have been introduced to the market for cancer therapy over 50 years [8,9,10,11,12,13,14,15]. To date, there have been three key binding sites in tubulin involving paclitaxel (Taxol), colchicine, and vinca alkaloid. These agents have also been divided into two categories: microtubule destabilizing agents and microtubule-stabilizing agents [16,17,18,19,20,21,22]. Particularly, microtubule-stabilizing agents (MSAs) were considered promising for clinical cancer treatment [23]. For instance, Taxol, the first diterpene isolated from the western yew, had been verified with excellent antiproliferative activity and was used in the clinic. Subsequently, docetaxel, obtained from the semisynthetic derivatives of Taxol, was also approved in 1996 by the Food and Drug Administration (FDA) for clinical treatment [24,25]. Moreover, epothilones and laulimalide, as natural microtubule-stabilizing agents that could promote tubulin self-assembly into microtubules, were also commercialized [26]. However, the wide application of these compounds was restricted due to some urgent problems, including high toxicity, limited sources, complex isolation processes, and the already discovered drug resistance [22]. To solve this issue, some chemically synthesized small molecules were persistently explored and actively developed as microtubule stabilizers, such as GS-164, Synstab A, 4′-methoxy-2-styrylchromone, and compound A (Figure 1) [22,27,28,29,30]. It is notable that *α*,*β*-unsaturated ketones, as the privileged chemical scaffolds, frequently appeared in the microtubule modulators, exemplified by curcumin and its derivatives that could inhibit tubulin self-assembly by interacting with the unique binding site of tubulin [22,31,32,33]. Meanwhile, these similar substrates have also been exploited as the correlative olefin polymer materials in the field of advanced materials science [34]. Herein, to discover some potential anti-cancer agents targeting tubulin based on the chemical modifications of natural ingredients of *α*,*β*-unsaturated carbonyl compounds, a series of facilely synthetic *N*-cinnamoyl-*N*′-(substituted)acryloyl hydrazide derivatives from raw material cinnamic acid were designed, synthesized, and screened for their anticancer activity in vitro (Figure 1). We expected that these designed frameworks would have the ability to disrupt tubulin assembly, which should be investigated by fluorescence imaging, tubulin polymerization assay and TEM imaging.

## 2. Results

### 2.1. Chemistry

**Scheme 1:**
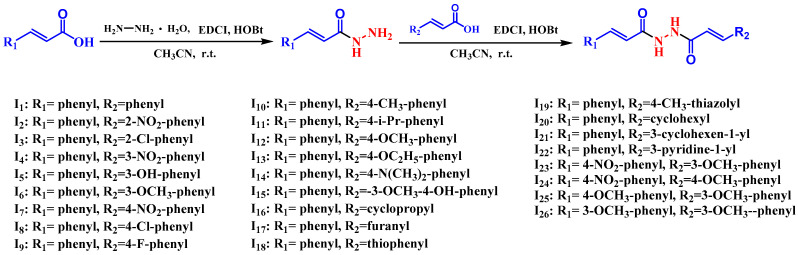
Synthetic route for the target molecules **I_1_**–**I_26_**.

To effectively obtain the target compounds, a facile synthetic route was designed as depicted in Scheme 1. Briefly, the cinnamic acid was reacted with 60% hydrazine hydrate, EDCI, and HOBt under basic conditions to give the intermediate cinnamohydrazide [35]. Then, the corresponding cinnamic acid analogue was reacted with cinnamohydrazide, EDCI, and HOBt and subsequently yielded the precipitates. The final target compounds **I_1_**–**I_22_** were afforded by being filtered and washed by CH_2_Cl_2_, respectively. Similarly, compounds **I_23_**–**I_26_** were synthesized according to the same reaction condition by changing to different starting substrates. Finally, these structures were confirmed by NMR and HRMS analysis.

### 2.2. The Antiproliferative Activity of Title Compounds

**Figure 2:**
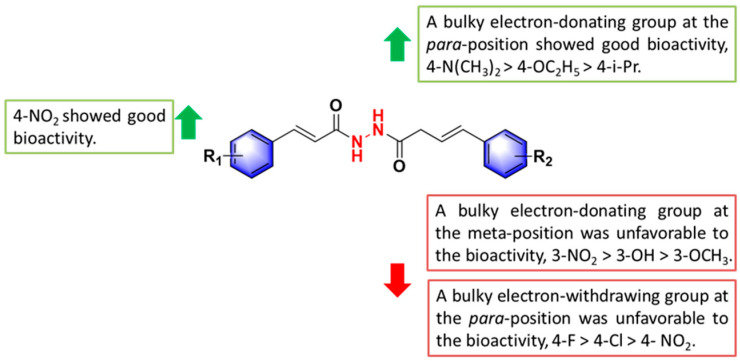
Description diagram of structure-activity relationship (SAR) analysis.

The antiproliferative activity of all the synthesized target compounds against A549, PC-3 and HepG2 cancer cells were evaluated using the MTT assay and expressed by IC_50_ values. As shown in Table 1, most of target compounds exerted certain antiproliferative activity against A549, PC-3, and HepG2. Among them, compounds **I_5_**, **I_7_** and **I_23_** displayed good antiproliferative activity (IC_50_ = 5.09 µM, 4.17 µM, 5.99 µM, respectively) against A549, which were significantly better than curcumin (IC_50_ = 69.6 µM) and similar to gefitinib (IC_50_ = 5.47 µM). In addition, compounds **I_1_**, **I_9_** and **I_23_** revealed excellent anti-PC-3 activity with IC_50_ values of 5.95 µM, 4.68 µM and 4.17 µM, respectively, which were equipotent to the positive drug gifitinib (5.99 µM). Moreover, compounds **I_9_**, **I_14_** and **I_23_** exhibited outstanding antiproliferative activity (IC_50_ = 4.75 µM, 3.42 µM, 3.36 µM, respectively) against HepG2, which were better than curcumin, gefitinib and colchicine (IC_50_ = 40.2, 31.5 and 5.46 µM, respectively). Particularly, compound **I_23_** displayed comprehensive antiproliferative activity with IC_50_ values of 5.99, 4.17 and 3.36 μΜ against A549, PC-3 and HepG2, respectively. It was superior to those of the curcumin and gefitinib. The structure–activity relationship (SAR) of target compounds against HepG2 cancer cells was further summarized as follows: (1) a bulky electron-donating group at the *meta*-position was unfavorable to the bioactivity, such as **I_4_** (3-NO_2_-Ph, 6.32 µM) > **I_5_** (3-OH-Ph, 8.69 µM) > **I_6_** (3-OCH_3_-Ph, 46.8 µM); (2) a bulky electron-donating group at the *para*-position showed good bioactivity, illustrated by **I_14_** (4-N(CH_3_)_2_-Ph, 3.42 µM) > **I_13_** (4-OC_2_H_5_-Ph, 15.4 µM) > **I_12_** (4-OCH_3_-Ph, 21.1 µM) > **I_11_** (4-i-Pr-Ph, 18.6 µM) and **I_10_** (4-CH_3_-Ph, 23.6 µM); (3) a bulky electron-withdrawing group at the *para*-position was unfavorable to the bioactivity, such as **I_9_** (4-F-Ph, 4.75 µM) > **I_8_** (4-Cl-Ph, 6.98 µM) > **I_9_** (4-NO_2_-Ph, 7.63 µM); (4) as R_1_ was 4-NO_2_, a 3-OCH_3_ located at another benzene ring was beneficial to bioactivity, **I_23_** (3-OCH_3_, 3.36 µM) and **I_24_** (4-OCH_3_, 8.08 µM). Finally, a detailed SAR analysis of target compounds is depicted in Figure 2.

To evaluate the selectivity of target compounds against cancer cell lines and normal cell lines, certain target compounds were evaluated by using the NRK-52E cell line. As Table 2 illustrates, compounds **I_5_** and **I_7_** showed good cytotoxicity against A549, PC-3 and HepG2 cell lines, but exhibited weaker cytotoxic towards NRK-52E cells. Meanwhile, compound **I_14_** revealed lower selectivity towards three cell lines and NRK-52E cells. Specially, compared with colchicine and gefitinib, compound **I_23_** afforded a relatively lower cytotoxicity towards normal NRK-52E cells, but exhibited the best cytotoxicity against three cell lines. Given these obtained results, a possible disrupting effect of compound **I_23_** on microtubule was further investigated.

### 2.3. Immunofluorescence Staining of Tubulin

**Figure 3:**
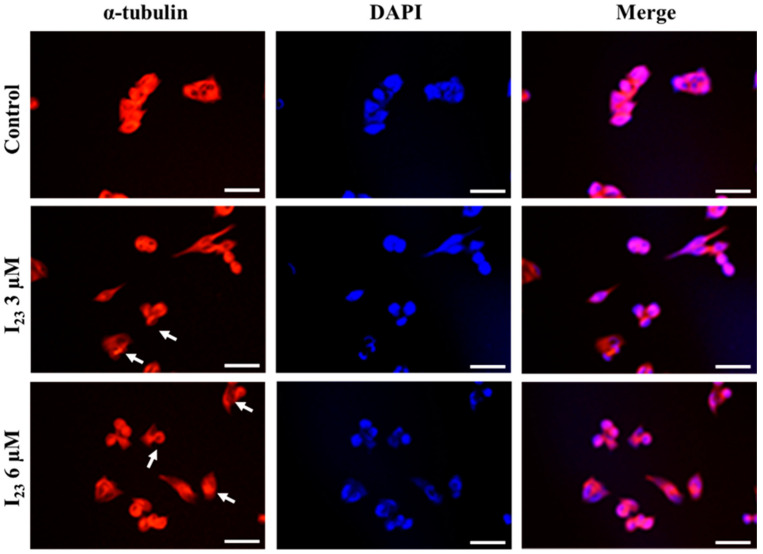
Effects of compound **I_23_** on the cellular microtubule network were visualized by immunofluorescence assay. HepG2 cells were treated with vehicle control 0.1% DMSO, 3 μM and 6 μM **I_23_** for 24 h. Then, cells were fixed and stained with anti-*α*-tubulin antibody (red) and counterstained with DAPI (blue). Detection of the fixed and stained cells was performed using fluorescence microscope. Scale bars are 50 μm.

The disrupting effect of microtubule triggered by compound **I_23_** in living cells was disclosed using the immunofluorescence staining assay. Primarily, the HepG2 cells were treated with 0, 3, and 6 μM of compound **I_23_** for 24 h before cell imaging. Clearly, the agminated microtubule network and wrinkled nucleus were observed in cells after incubating with compound **I_23_**, thereby leading to the morphological change in HepG2 cells (Figure 3). By contrast, the cellular microtubule network was well assembled and arranged normally in the control group. This result revealed that compound **I_23_** might be capable of leading to cell cycle disorder through disturbing microtubule assembly and targeting tubulin.

### 2.4. Effects of Compound ***I_23_*** on Tubulin Polymerization

**Figure 4:**
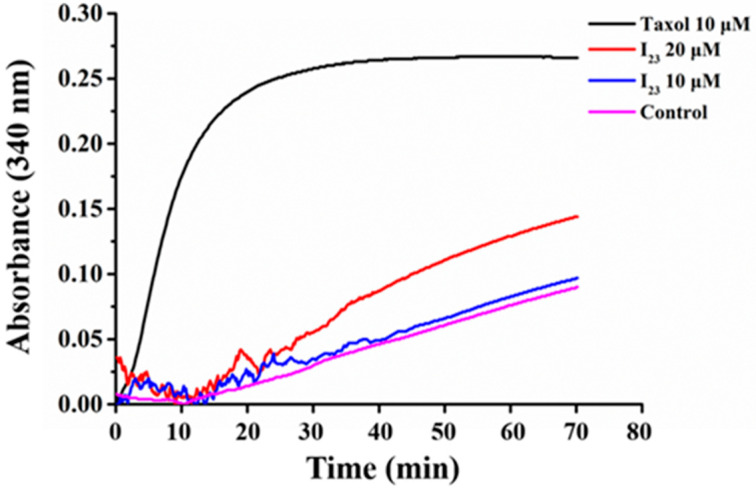
Progress of tubulin polymerization in the presence of 10 μM and 20 μM compound **I_23_**. Taxol (10 μM) was used as positive polymerization control, whereas untreated tubulin was used as negative control.

Tubulin polymerization assay [36,37,38] triggered by compound **I_23_** was carried out to verify the interaction mode. Meanwhile, paclitaxel, served as a known microtubule stabilizer that could promote the protofilament assembly. As shown in Figure 4, similar to paclitaxel, compound **I_23_** could stabilize tubulin assembly and promote protofilament assembly in a dosage-dependent manner, which was consistent with the outcome from previous reports [38,39], indicating that the potential microtubule-stabilizer might be developed.

### 2.5. Tubulin Polymerization Affected by Compound ***I_23_*** via TEM

**Figure 5:**
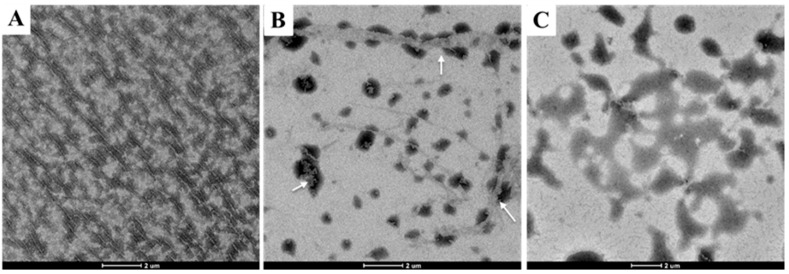
Effect of different concentrations of compound **I_23_** on tubulin polymerization: (**A**) 4 mg/mL tubulin, (**B**) 20 μM **I_23_** + 4 mg/mL tubulin, (**C**) 20 μM **I_23_** without tubulin. Scale bars are 2 μm.

Transmission electron microscopy (TEM) was employed to directly visualize the influence of tubulin polymerization stimulated by compound **I_23_**. Evidently, without the addition of compound **I_23_**, the observed microtubules presented uniform fibrous nanostructures, suggesting the spontaneous formation of microtubules occurred in general tubulin buffer solution (Figure 5A). However, the spontaneous assembly of *α*/*β* tubulin heterodimers was disturbed by the existence of 20 μM compound **I_23_**, subsequently affording more large nonlinear disorganized aggregations (Figure 5B). These microscopic investigation results manifested that **I_23_** could promote the microtubular aggregation.

### 2.6. Cell Cycle Analysis

**Figure 6:**
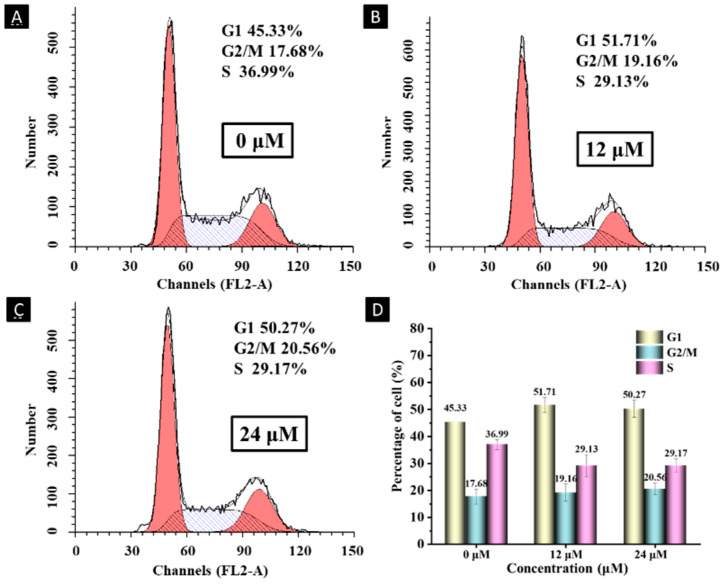
Compound **I_23_** affected the cell cycle distribution in HepG2 cancer cells. Untreated (Control) and cells treated with compound **I_23_** at 24 μM concentration for 24 h.

Microtubules play a critical role during the eukaryotic cell division [40]. Based on the above results, compound **I_23_** could clearly affect the tubulin polymerizations, and this event probably led to the stagnation of cell cycle. To investigate this effect, flow cytometry analysis on HepG2 cell cycle arrest was performed as Figure 6A–C, and the corresponding results were displayed as Figure 6D. Interestingly, the G2/M pattern was arrested after treatment with compound **I_23_** (24 μM), thus providing the relevant percentages from 17.68% (0 μM) to 20.56% (Figure 6), indicating the designed compound could cause cell cycle disorder through targeting tubulin and the subsequent disturbance on microtubule assembly.

### 2.7. Molecular Docking of Compound ***I_23_*** with Tubulin

**Figure 7:**
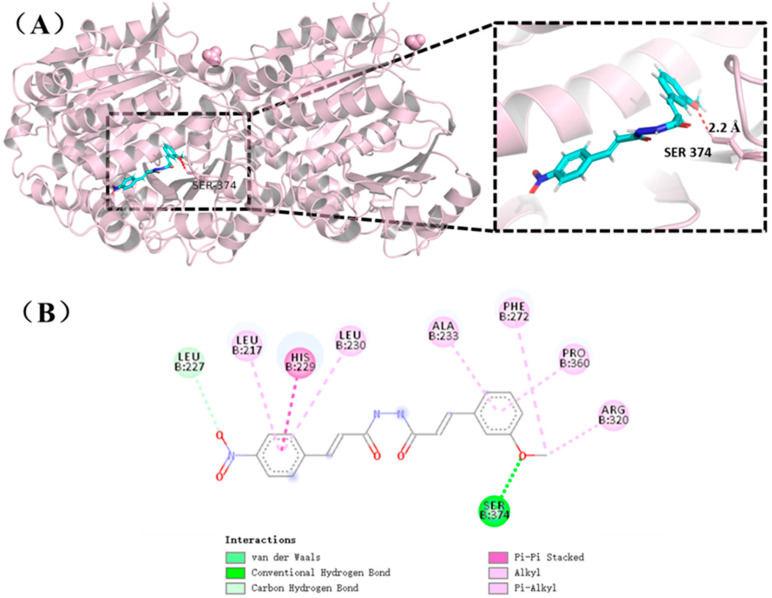
Predicted binding modes of compound **I_23_** with tubulin. Binding of **I_23_** into the active site of tubulin was performed by PyMol software (**A**) and the 2D diagram of binding modes was shown through Discovery Studio 4.5 software (**B**).

To better understand the possible interaction mode and binding sites between compound **I_23_** and tubulin, the related software Sybyl X 2.0, PyMOL and Discovery Studio (DS) 2020 were exploited. Meanwhile, the reported 3D crystal structure of tubulin (PDB code: 5syf) was used by the removal of the ligand molecule paclitaxel. Docking study (Figure 7) displayed that compound **I_23_** could embed in the active pocket around with the amino acid residues, including Leu227, Leu217, His229, Leu230, Ala233, Phe272, Pro360, Arg320, and Ser374. Apart from conventional hydrogen bonds and carbon hydrogen bonds, other non-covalent interactions including π-donor hydrogen bonds, π-alkyl, etc., also were very crucial donations for the interaction [41,42,43]. In detail, the Ser374 residue could form strong hydrogen bond interactions with compound **I_23_**, thus affording a distance of 2.2 Å located in the S9–S10 stabilizing loop of tubulin, which was the binding site of Taxol. The correlative Ala233, Leu217, Leu230, Phe272, Pro360, and Arg320 residues could form π-alkyl interactions. Simultaneously, π-π stacked interaction was also observed between His229 residue and compound **I_23_**. These results indicated that a strong interaction occurred between the designed compound and tubulin, thereby disturbing the normal assembly of tubulin, which was consistent with the aforementioned tubulin polymerization assays.

### 2.8. Effects on Cell Migration of Compound ***I_23_***

**Figure 8:**
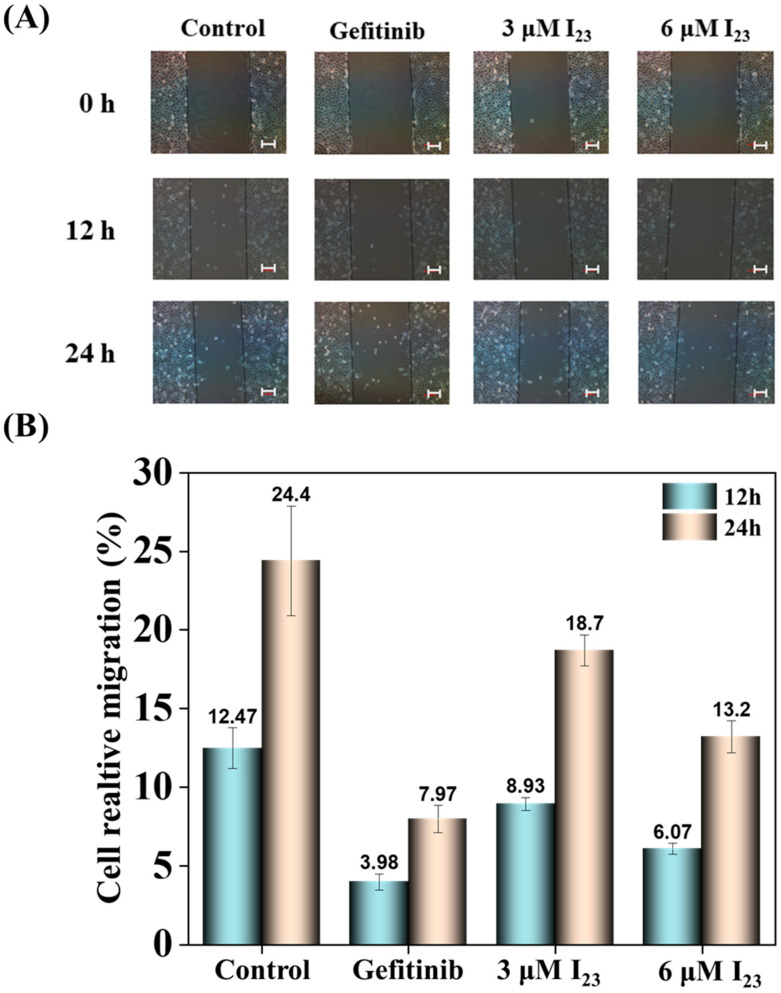
Cell migration assay showed that compound **I_23_** inhibited the migration of A549 cells. (**A**) The cancer cells were incubated with 0 μM, 3 μM, 6 μM compound **I_23_**, the 6 μM gefitinib was served as the positive control. Scale bars are 100 μm (**B**) the cell relative migration was calculated using the software.

Because the targeting-tubulin agents were validated, having the ability to interfere with cell migration [44,45], the cell migration effect triggered by compound **I_23_** was investigated by the calculation of average migration rates of scratched A549 cells monolayer, which was a widely used model. As depicted in Figure 8, a 12.5% migration rate was found in the control after 12 h, while the decreased migration rates reached to 8.90%, 6.10%, and 3.98% after treatment of 3 μM **I_23_**, 6 μM **I_23_**, and 6 μM gefitinib, respectively. After incubation for another 12 h, the migration rate of A549 cells that coexisted with compound **I_23_** changed to 13.2% (blank control reached to 24.4%). This outcome indicated that compound **I_23_** could significantly attenuate the migration of A549 cells in a dosage-dependent manner, which could be a lead structure for the exploration of novel microtubule-stabilizing agents.

### 2.9. Apoptosis Effects of HepG2 Cells Caused by Compound ***I_23_***

**Figure 9:**
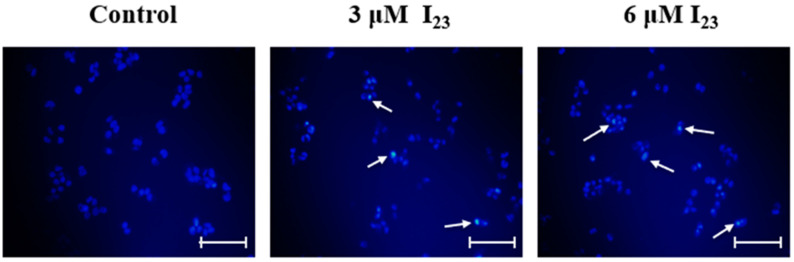
The cells were incubated with 0 μM, 3 μM and 6 μM compound **I_23_** for 24 h, subsequently stained with Hoechst 33258 (10 μg/mL) and visualized by fluorescence microscopy. Scale bars are 100 μm.

To date, many reports have demonstrated that anti-mitotic cancer agents could also cause cell death through inducing apoptosis [46,47]. Thus, the apoptotic behavior of HepG2 cells triggered by compound **I_23_** should be tested since an appreciable antiproliferative activity and substantial disturbance on microtubule assembly were achieved. As shown in Figure 9, apoptosis effects were observed after incubating cells with 0 μM, 3 μM and 6 μM of compound **I_23_**, respectively. Particularly, as the dose was up to 6 μM, a strong apoptotic phenomenon happened, indicating that compound **I_23_** acting as a potential microtubule-stabilizer could slightly induce HepG2 cancer cells apoptosis.

### 2.10. In Silico Drug-likeness Evaluation

**Figure 10:**
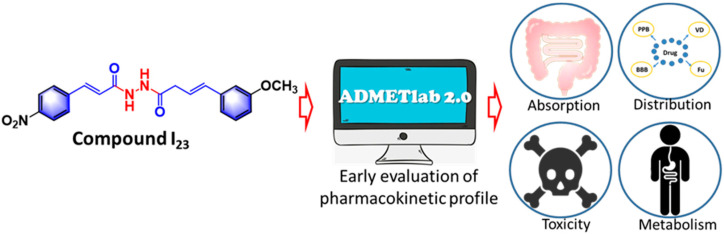
The predicted model of pharmacokinetic profile for compound **I_23_**.

**Figure 11:**
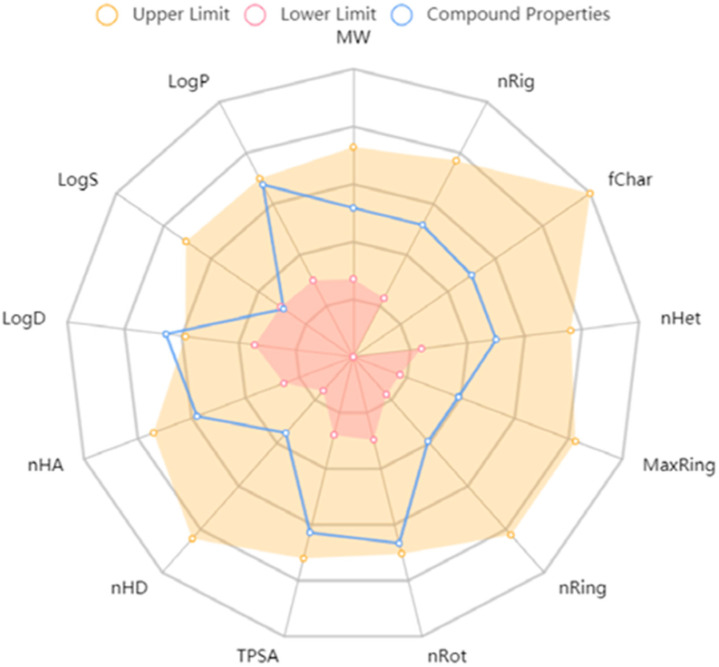
The physicochemical properties of compound **I_23_**.

Finally, the early evaluation of the lead compound for its potential is a critical step in drug development. Therefore, to assess whether compound **I_23_** has the potential as the promising lead compound, ADMETlab 2.0 software was used to obtain more information on the pharmacokinetic profile, including ADMET and drug-likeness properties (Figure 10). The corresponding physicochemical properties, ADMET, and drug-likeness properties of compound **I_23_** are presented in Figure 11 and Table 3, respectively. Notably, the outcomes illustrated that compound **I_23_** exhibited acceptable physicochemical properties, ADMET, and drug-likeness properties. For instance, compound **I_23_** possessed favorable physicochemical properties: molecular weight = 369.13, logS = −4.140, logP = 2.821, and log D = 3.559. Meanwhile, ADMET and drug-likeness properties of compound **I_23_** were provided as follows: (1) compound **I_23_** had appreciable absorption potency: for example, compound **I_23_** was active in both human intestinal absorption (HIA) and had 20% bioavailability (F20%); (2) compound **I_23_** was active in blood–brain barrier (BBB) penetration; (3) compound **I_23_** possessed acceptable safety profiles (e.g., hERG blockers, eye corrosion and respiratory toxicity), and displayed some satisfactory metrics on metabolism (See support information Table S1) and excretion potency; and (4) more interestingly, compound **I_23_** met all drug-likeness properties including Lipinski rule, Pfzer rule, Golden triangle, and GSK rule. Based on the above-mentioned outcomes, compound **I_23_** displayed an acceptable pharmacokinetic profile, making it a promising lead compound to excavate and discover novel microtubule stabilizers.

## 3. Experimental Section

### 3.1. Instruments and Chemicals

NMR spectra were performed using the JEOL-ECX500 instrument (Akishima, Japan) or Bruker Biospin AG-400 instrument (Bruker Optics, Ettlingen, Germany) using DMSO-*d*_6_ as the solvent and tetramethylsilane as the internal standard. HRMS spectra were obtained on Waters Xevo G2-S QTOF MS (Waters MS Technologies, Manchester, UK). Immunofluorescence staining assay was performed by the tubulin-tracker red kit (Beyotime Institute of Biotechnology, Shanghai, China) and observed using a Nikon ECLIPSE Ti—S fluorescent microscope (Nikon, Tokyo, Japan). The tubulin polymerization assay was performed by the tubulin polymerization assay kit (cytoskeleton, ^#^BK004P) and recorded by Cytation™ 5 multi-mode readers (BioTek Instruments, Inc., Winooski, VT, USA). The trans-cinnamic acid and analogues were purchased from Aladdin Industrial Inc. (Shanghai, China). The FACSCalibur™ flow cytometer (Becton Dickinson Immunocytometry Systems, San Jose, CA, USA) was employed to analyze the cell cycle arrest.

### 3.2. Pharmacology

#### 3.2.1. Cell Culture

An A549 (human non-small cell lung cancer cell line) cell line was purchased from the Shanghai Cell Bank of the Chinese Academy of Sciences; PC-3 (human prostate cancer cell line) cell line was donated by the Key Laboratory of Natural Product Chemistry of the Chinese Academy of Sciences of Guizhou Province; HepG2 (human liver cancer cell line) and NRK 52E (normal rat kidney cell line) cell line was donated by Guizhou Medical University; all cell lines were kept by the laboratory. Cells were maintained in RPMI 1640 or DMEM complete medium.

#### 3.2.2. MTT Assay

The antiproliferative activity of target compounds against A549, PC-3, HepG2, and NRK-52E were determined by MTT assay, as described in previous articles [48,49,50]. The assay was recorded at 490 nm by an Infinite^®^ M200 PRO multimode microplate (Tecan, Männedorf, Switzerland). Curcumin, gefitinib, and colchicine were used as positive controls.

#### 3.2.3. Immunofluorescence Staining Pattern

According to the previous methods [51,52], compound solutions of various concentrations were added to a 6-well plate in which HepG2 cells had been seeded for 12 h. After incubation for 24 h and washed with phosphate-buffered saline (PBS, 10 mM, pH 7.3), each well was treated with 4% formaldehyde solution for 15 min and washed with 0.1% Triton X-100 of phosphate-buffered saline (PBS, 10 mM, pH 7.3). After that, the fixed cells were incubated with diluted tubulin-tracker red for 40 min in a dark environment, followed by washing three times by 0.1% Triton X-100 of phosphate-buffered saline (PBS, 10 mM, pH 7.3). Finally, each well was added DAPI (2 μg/mL). Finally, the results were observed using a Nikon ECLIPSE Ti-S fluorescent microscope (Nikon Co, Tokyo, Japan).

#### 3.2.4. Tubulin Polymerization Assay In Vitro

The tubulin polymerization assay in vitro was determined by HTS-Tubulin Polymerization Assay Kit (#BK004P, Cytoskeleton, Inc., Denver, CO, USA) as described in previous articles [36,37,38,53,54]. The plate was pre-warmed at 37 °C, and the reaction assay contained 100 μL 4 mg/mL tubulin in G-PEM buffer. Then, 10 μL (10×) compounds solution or 100 μM paclitaxel solution as a control. Finally, the polymerization was carried out at 37 °C and recorded at 340 nm each 10 s for 70 min using Cytation™ 5 multi-mode readers.

#### 3.2.5. Tubulin Polymerization Affected by Target Compounds via TEM

Briefly, according to the protocol of tubulin polymerization assay [38,39], the 100 μL centrifuge tubes were pre-warmed at 37 °C, and the reaction assay contained 20 μL 4 mg/mL tubulin in G-PEM buffer. Then, 2 μL (10×) compounds solution was added. Finally, the assay was carried out at 37 °C for 30 min. The formed microtubules were transferred to Formvar-caron-coated copper grids, negatively stained with 1% phosphotungstic acid, and visualized under a transmission electron microscope.

#### 3.2.6. Cell Cycle Analysis

The cells in the 6-well plates were carefully collected 24 h after dosing. After the cells were washed with pre-cooled PBS, the pre-cooled 70% ethanol solution was added for fixation (4 °C overnight). After that, the cells were washed with PBS, and incubated with 1 mg/mL RNase A for 30 min at 37 °C). Then, 20 mg/mL PI staining solution was added and incubated in the dark for 30 min at 4 °C. Finally, the cell arrest was analyzed by the FACSCalibur™ flow cytometer (Becton Dickinson Immunocytometry Systems, San Jose, CA, USA) as previously described [40].

#### 3.2.7. Computational Docking Studies

The docking study was performed by Sybyl X 2.0 and the tubulin protein (PDB: 5syf) was downloaded from RCSB Protein Data Bank (www.rcsb.org, accessed on 1 October 2020.). The protein and all ligands were prepared by minimization with the CHARMM force field. Molecular docking was carried out using Sybyl X 2.0 protocol without constraint. All bound water and ligands were eliminated from the protein and the polar hydrogen was added to the proteins. The docking results were performed by PyMol software and Discovery Studio (DS) 2020 [55,56,57].

#### 3.2.8. Scratch Test

According to the previous methods [58], A549 cells of the logarithmic growth stage were cultured in 6-well plates with two lines on the back, with each well containing a density of 1 × 10^6^ cells/mL. After the cells adhered to the wall, three uniform thin lines were drawn in each well by a sterile pipette tip. Afterward, the medium containing 1% fetal bovine serum (FBS) and various concentrations of compounds were added in wells. After the different time of incubation, the cells were performed by an inverted fluorescence microscope. Then the scratch healing rate was calculated: Migration distance (n h) = edge distance (0 h) − edge distance (12 h or 24 h).

#### 3.2.9. Hoechst Apoptosis Experiment

The nuclear morphological modifications were exhibited by fluorescence pattern. In this assay [58,59,60,61], HepG2 cells were seeded in 6-well plates, after 24 h of incubation, different compounds were added at various concentrations, and cells were incubated again for 24 h. Afterward, the medium was removed and cells were washed twice with phosphate-buffered saline (PBS, 10 mM, pH 7.3), and fixed with 4% paraformaldehyde for 15 min at room temperature, then stained with 10 mg/mL Hoechst 33258 in PBS for 20 min at 37 °C in the dark. After incubation, the cells were incubated with an anti-fluorescent attenuator and imaged with an inverted fluorescence microscope.

#### 3.2.10. In Silico Pharmacokinetics

The structures of compound **I_23_** were drawn using ChemDraw (version Ultra 12.0, PerkinElmer Informatics, Waltham, MA, USA) and transformed as SMILES format. In silico drug-likeness predictions were conducted using http://www.swissadme.ch/index.php accessed on 1 May 2022 [62,63].

## 4. Conclusions

To develop novel microtubule-binding agents for cancer therapy, an array of diacylhydrazine-functionalized cinnamic acid derivatives were facilely synthesized through a two-step process. Antiproliferative bioassays showed that compound **I_23_** exhibited the best antiproliferative activity against three cancer lines with IC_50_ values ranging from 3.36 to 5.99 μM and yielded a lower cytotoxicity towards the normal cell line. Anticancer mechanism investigations suggested that the highly bioactive compound **I_23_** could potentially promote the protofilament assembly of tubulin through fluorescence imaging, tubulin polymerization assay and TEM imaging, thus eventually leading to the stagnation of G2/M phase cell cycle of HepG2 cells. Meanwhile, molecular docking studies revealed that compound **I_23_** could interact with Ser374 residue, Ala233, Leu217, Leu230, Phe272, Pro360, and Arg320 residues, and His229 residue of tubulin directed by the corresponding hydrogen bond interactions, π-alkyl interactions, and π-π stacked interactions, which was similar with that of Taxol located at the S9-S10 stabilizing loop of tubulin. Furthermore, compound **I_23_** could also reduce the cancer cell migration rate and induce HepG2 cells apoptosis. Additionally, the in silico analysis indicated that compound **I_23_** exhibited an acceptable pharmacokinetic profile. Based on the present findings, these simple hydrazide derivatives could be considered as potential microtubule-stabilizer lead structures for future anticancer drug discovery.

## Figures and Tables

**Table 1:** The antiproliferative activity of all designed compounds against cancer cell lines ^a^.

No.	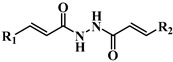		IC_50_ (μM) ^b^		
	R_1_	R_2_	A549	PC-3	HepG2
**I_1_**	-phenyl	-phenyl	26.7 ± 0.43	5.95 ± 1.52	8.17 ± 0.46
**I_2_**	-phenyl	2-NO_2_-phenyl	7.55 ± 1.22	8.08 ± 2.24	9.33 ± 0.95
**I_3_**	-phenyl	2-Cl-phenyl	6.48 ± 0.41	7.38 ± 0.24	9.53 ± 1.74
**I_4_**	-phenyl	3-NO_2_-phenyl	6.05 ± 0.08	15.8 ± 1.67	6.32 ± 1.00
**I_5_**	-phenyl	3-OH-phenyl	5.09 ± 0.08	6.68 ± 0.96	8.69 ± 1.04
**I_6_**	-phenyl	3-OCH_3_-phenyl	41.0 ± 4.47	160 ± 10.9	46.8 ± 0.14
**I_7_**	-phenyl	4-NO_2_-phenyl	4.17 ± 0.93	8.79 ± 3.19	7.63 ± 1.75
**I_8_**	-phenyl	4-Cl-phenyl	9.57 ± 1.92	63.2 ± 3.86	6.98 ± 1.30
**I_9_**	-phenyl	4-F-phenyl	24.9 ± 1.54	4.68 ± 0.69	4.75 ± 0.36
**I_10_**	-phenyl	4-CH_3_-phenyl	19.5 ± 4.95	70.3 ± 9.88	23.6 ± 2.67
**I_11_**	-phenyl	4-i-Pr-phenyl	43.0 ± 7.30	19.2 ± 6.57	18.6 ± 3.90
**I_12_**	-phenyl	4-OCH_3_-phenyl	10.9 ± 0.25	39.4 ± 3.54	21.1 ± 4.07
**I_13_**	-phenyl	4-OC_2_H_5_-phenyl	15.0 ± 4.90	22.5 ± 2.64	15.4 ± 0.93
**I_14_**	-phenyl	4-(CH_3_)_2_N-phenyl	12.8 ± 0.60	7.81 ± 1.69	3.42 ± 0.49
**I_15_**	-phenyl	3-OCH_3_-4-OH-phenyl	35.8 ± 0.59	>300	31.1 ± 7.66
**I_16_**	-phenyl	cyclopropyl	16.4 ± 0.98	80.5 ± 5.43	78.5 ± 1.39
**I_17_**	-phenyl	furanyl	13.0 ± 2.40	36.0 ± 0.70	34.5 ± 0.28
**I_18_**	-phenyl	thiophenyl	13.8 ± 0.29	15.9 ± 1.14	21.1 ± 3.76
**I_19_**	-phenyl	4-CH_3_-5-thiazolyl	26.6 ± 5.08	96.4 ± 8.36	89.3 ± 8.89
**I_20_**	-phenyl	cyclohexyl	16.3 ± 3.25	21.8 ± 3.15	>300
**I_21_**	-phenyl	3-cyclohexen-1-yl	8.27 ± 0.80	21.6 ± 0.89	20.4 ± 0.86
**I_22_**	-phenyl	3-pyridine-1-yl	60.2 ± 2.37	159 ± 5.88	43.3 ± 1.10
**I_23_**	4-NO_2_-phenyl	3-OCH_3_-phenyl	5.99 ± 1.07	4.17 ± 0.57	3.36 ± 0.80
**I_24_**	4-NO_2_-phenyl	4-OCH_3_-phenyl	5.17 ± 0.41	15.7 ± 2.98	8.08 ± 2.25
**I_25_**	4-OCH_3_-phenyl	3-OCH_3_-phenyl	144 ± 8.95	142 ± 6.79	80.0 ± 4.04
**I_26_**	3-OCH_3_-phenyl	3-OCH_3_-phenyl	11.0 ± 1.72	21.0 ± 2.32	14.5 ± 2.56
Curcumin			69.6 ± 1.18	73.4 ± 4.99	40.2 ± 1.74
Colchicine			1.35 ± 0.08	2.12 ± 0.29	5.46 ± 1.76
Gefitinib			5.47 ± 1.06	5.99 ± 1.47	31.5 ± 10.2

^a^ The anti-proliferation activities of all target compounds against cancer cells were determined by MTT assay after 48 h. The data represent the mean of triplicate determinations. ^b^ IC_50_ values are indicated as the mean ± SD (standard error) of at least three independent experiments.

**Table 2:** Cytotoxicity and selectivity of title compounds **I_5_**, **I_7_**_,_ **I_14_** and **I_23_** ^a^.

No.	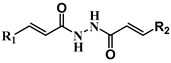		IC_50_ (μM) ^b^			
	R_1_	R_2_	A549	PC-3	HepG2	NRK-52E
**I_5_**	phenyl	3-OH-phenyl	5.09 ± 0.08	6.68 ± 0.96	8.69 ± 1.04	50.0 ± 4.40
**I_7_**	phenyl	4-NO_2_-phenyl	4.17 ± 0.93	8.79 ± 3.19	7.63 ± 1.75	21.0 ± 1.75
**I_14_**	phenyl	4-(CH_3_)_2_N-phenyl	12.8 ± 0.60	7.81 ± 1.69	3.42 ± 0.93	7.00 ± 0.30
**I_23_**	4-NO_2_-phenyl	3-OCH_3_-phenyl	5.99 ± 1.07	4.17 ± 0.57	3.36 ± 0.80	6.99 ± 1.60
Curcumin			69.6 ± 1.18	73.4 ± 4.99	40.2 ± 1.74	>300
Colchicine			1.35 ± 0.08	2.12 ± 0.29	5.46 ± 1.76	3.32 ± 0.81
Gefitinib			5.47 ± 1.06	5.99 ± 1.47	31.5 ± 10.2	21.0 ± 2.10

^a^ The anti-proliferation activities of all target compounds against cancer cells were determined by MTT assay after 48 h. The data represent the mean of triplicate determinations. ^b^ IC_50_ values are indicated as the mean ± SD (standard error) of at least three independent experiments.

**Table 3:** ADMET and drug-likeness properties of compound **I_23_** through the online prediction tool ADMETlab 2.0.

Property	Value	Decision
Absorption		
Caco-2 Permeability	−4.82	Excellent
Madin–Darby canine kidney cells (MDCK) permeability	0.000185	Excellent
P-glycoprotein (Pgp)-inhibitor	0.984	Bad
P-glycoprotein (Pgp)-substrate	0.002	Excellent
Human intestinal absorption (HIA)	0.021	Excellent
20% bioavailability (F20%)	0.002	Excellent
Distribution		
Plasma protein binding (PPB)	95.57%	Bad
Volume distribution (VD)	0.285	Excellent
Blood–brain barrier (BBB) penetration	0.038	Excellent
The fraction unbound in plasma (Fu)	1.635%	Bad
Excretion		
Clearance	7.066	Excellent
The half-life (T_1/2_)	0.404	
Toxicity		
hERG blockers	0.329	Medium
Rat oral acute toxicity	0.195	Excellent
Maximum recommended daily dose (FDAMDD)	0.449	Medium
Skin sensitization	0.916	Bad
Eye corrosion	0.004	Excellent
Eye irritation	0.305	Medium
Respiratory toxicity	0.145	Excellent
Drug-likeness		
Lipinski rule	Accepted	Excellent
Pfzer rule	Accepted	Excellent
Golden triangle	Accepted	Excellent
GSK rule	Accepted	Excellent

## Data Availability

Not applicable.

## Supplementary Materials

The following supporting information can be downloaded at: https://www.mdpi.com/article/10.3390/ijms232012365/s1, References [35,63] are cited in the supplementary materials.
