# Supplementary material for: Discovery of Simple Diacylhydrazine-Functionalized Cinnamic Acid Derivatives as Potential Microtubule Stabilizers
Source: Int J Mol Sci. 2022 Oct 15;23(20):12365. doi: 10.3390/ijms232012365 (PMC9604255; doi:10.3390/ijms232012365)
Supplement: Supplementary file 1 [file ijms-23-12365-s001.zip › ijms-1925196-supplementary.pdf]

Discovery of simple diacylhydrazine-functionalized cinnamic acid derivatives as potential microtubule stabilizers

Xiang Zhou [1,*,†], Yi-Hong Fu [2,†], Ya-Yu Zou [3], Jiao Meng [1], Gui-Ping Ou-Yang [3], Qiang-Sheng Ge [3], and Zhen-Chao Wang [1,3,*]

[1] State Key Laboratory Breeding Base of Green Pesticide and Agricultural Bioengineering, Key Laboratory of Green Pesticide and Agricultural Bioengineering, Ministry of Education, Center for R & D of Fine Chemicals of Guizhou University, Guiyang 550025, China

[2] Science and Technology College of Hubei University of Arts and Science, Xiangyang 441025, China

[3] College of Pharmacy, Guizhou University, Guiyang 550025, China

* Correspondence: xiangzhou@gzu.edu.cn or zhoux1534@163.com (X.Z.); zcwang@gzu.edu.cn or wzc.4884@163.com (Z.-C.W.); Tel./Fax: +86-851-8830-8717 (Z.C.W.)

† These authors contributed equally to this work.

1. The metabolism property of compound I_{23}.

Table S1 The metabolism property of compound I_{23} through the online prediction tool ADMETlab 2.0.

Metabolism Property	Value	Decision
Metabolism		
CYP1A2 inhibitor	0.109	
CYP1A2-substrate	0.182	
CYP2C19-inhibitor	0.373	
CYP2C19-substrate	0.125	
CYP2C9-inhibitor	0.197	
CYP2C9-substrate	0.883	
CYP2D6-inhibitor	0.008	
CYP2D6-substrate	0.337	
CYP3A4-inhibitor	0.517	
CYP3A4-substrate	0.441	

2. General procedure for the synthesis of intermediate A

The intermediate cinnamohydrazide was prepared according to the reported method [1-2]. A mixture of 2.0 g of cinnamic acid (13.50 mmol), 3.11 g of EDCI (16.20 mmol) and 2.19 g of HOBt (16.20 mmol) was dissolved in 300 mL of acetonitrile. Then 0.5 mL triethylamine was added dropwise, and the mixture was stirred at room temperature for 2 h prior to adding 1.00 mL 60% hydrazine hydrate. After the reaction is completed, the filtrate was evaporated using a rotary evaporator and extracted with ethyl acetate (5×50 mL), the organic layer was washed by 30% solution of sodium carbonate and water, dried with sodium sulfate, and followed by the removal of the solvent under vacuum. Finally, the desired cinnamohydrazide was purified by a silica gel using ethyl

acetate as the eluent. A white solid, yield 61.1%; ^{1}H NMR (500 MHz, DMSO-*d6*) δ 9.28 (s, ^{1}H, CO-NH-NH_2), 7.51 (d, *J* = 7.3 Hz, 2H, phenyl-2-H+phenyl-6-H), 7.44–7.30 (m, 4H phenyl-3-H+phenyl-4-H phenyl-5-H+phenyl-CH=CH), 6.51 (d, *J* = 15.9 Hz, 1H phenyl-CH=CH), 4.40 (d, *J* = 3.9 Hz, 2H, CO-NH-NH_2); ^{13}C NMR (126 MHz, DMSO-*d6*) δ 165.0, 138.7, 135.5, 129.9, 129.5, 128.0, 120.9.

3. General procedure for the synthesis of title compounds.

The target compound *N'*-cinnamoylcinnamohydrazide was prepared according to the reported method [1-2]. A mixture of 0.30 g of cinnamic acid (1.88 mmol) and 0.5 mL triethylamine was dissolved in 25 mL of acetonitrile. Then, 0.43 g of EDCI (2.26 mmol), 0.30 g of HOBt (2.26 mmol), and 0.30 g cinnamohydrazide (1.88 mmol) were added into the mixture, respectively. The reaction was stirred at room temperature for 2 h. After the reaction was completed, the crude product was filtered and further purified by washing with CH_2Cl_2. A white solid, yield 78%.

4. The data of title compounds.

***N'*-cinnamoylcinnamohydrazide (I_1)** A white solid, yield 75.0%; ^{1}H NMR (500 MHz, DMSO-*d6*) δ 10.51 (s, 2H, CO-NH-NH-CO), 7.61 (d, *J* = 7.0 Hz, 4H, phenyl-2-H + phenyl-6-H), 7.56 (d, *J* = 15.9 Hz, 2H, phenyl-CH=CH), 7.46-7.39 (m, 6H, phenyl-3-H + phenyl-4-H + phenyl-5-H), 6.78 (d, *J* = 15.9 Hz, 2H, phenyl-CH=CH); ^{13}C NMR (126 MHz, DMSO-*d6*) δ 163.5, 140.6, 135.2, 130.4, 129.6, 128.2, 119. 9. HRMS (ESI) $[M+H]^+$ calcd for $C_{18}H_{17}O_2N_2$: 293.1285, found: 293.1280.

***(E)*-*N'*-cinnamoyl-3-(2-nitrophenyl)acrylohydrazide (I_2)** A yellow solid, yield 71.0%; ^{1}H NMR (500 MHz, DMSO-*d6*) δ 10.69 (s, 1H, 2-nitrophenyl-CH=CH-CO-NH), 10.58 (s, 1H, phenyl-CH=CH-CO-NH), 8.08 (d, *J* = 8.5 Hz, 1H, 2-nitrophenyl-3-H), 7.82 (dd, *J* = 13.8, 7.6 Hz, 3H, 2-nitrophenyl-CH=CH + 2-nitrophenyl-5-H + 2-nitrophenyl-6-H), 7.69–7.65 (m, 1H, 2-nitrophenyl-4-H), 7.64–7.60 (m, 2H, phenyl-2-H + phenyl-6-H), 7.57 (d, *J* = 15.9 Hz, 1H, phenyl-CH=CH), 7.47–7.38 (m, 3H, phenyl-3-H + phenyl-4-H + phenyl-5-H), 6.77 (q, *J* = 15.7, 14.4 Hz, 2H, phenyl-CH=CH + 2-nitrophenyl-CH=CH); ^{13}C NMR (126 MHz, $CDCl_3$) δ 163.5, 162.6, 148.8, 140.8, 135.8, 135.1, 134.5, 131.0, 130.4,130.4, 129.5, 129.4, 128.3, 125.3, 124.5, 119.8. HRMS (ESI)

$[M+H]^+$ calcd for $C_{18}H_{16}O_3N_4$: 338.1135, found: 338.1132.

***(E)*-3-(2-chlorophenyl)-*N'*-cinnamoylacrylohydrazide (I$_3$)** A white solid, yield 65.0%; ^{1}H NMR (500 MHz, DMSO-*d$_6$*) δ 10.63 (s, 1H, 2-chlorophenyl-CH=CH-CO-NH), 10.56 (s, 1H, phenyl-CH=CH-CO-NH), 7.83 (d, *J* = 15.8 Hz, 1H, 2-chlorophenyl-CH=CH), 7.76 (dd, *J* = 5.6, 3.7 Hz, 1H, 2-chlorophenyl-3-H), 7.66–7.51 (m, 4H, 2-nitrophenyl-5-H + 2-nitrophenyl-6-H + phenyl-2-H + phenyl-6-H), 7.49–7.36 (m, 5H, phenyl-CH=CH + phenyl-3-H + phenyl-4-H + phenyl-5-H + 2-chlorophenyl-4-H), 6.84 (d, *J* = 15.7 Hz, 1H, 2-nitrophenyl-CH=CH), 6.79 (d, *J* = 15.9 Hz, 1H, phenyl-CH=CH); ^{13}C NMR (126 MHz, DMSO-*d$_6$*) δ 163.5, 163.0, 140.7, 135.8, 135.1, 134.0, 133.0, 131.8, 130.6, 130.4, 129.6, 128.4, 128.3, 123.1, 119.8. HRMS (ESI) $[M+H]^+$ calcd for $C_{18}H_{16}O_2N_2Cl$: 327.0895, found: 327.0897.

***(E)*-*N'*-cinnamoyl-3-(3-nitrophenyl)acrylohydrazide (I$_4$)** A yellow solid, yield 58.0%; ^{1}H NMR (500 MHz, DMSO-*d$_6$*) δ 10.55 (s, 2H, CO-NH-NH-CO), 8.42 (s, 1H, 3-nitrophenyl-2-H), 8.20 (d, *J* = 7.9 Hz, 1H, 3-nitrophenyl-4-H), 8.03 (d, *J* = 6.8 Hz, 1H, 3-nitrophenyl-6-H), 7.74–7.63 (m, 2H, phenyl-2-H + phenyl-6-H), 7.56 (dd, *J* = 23.1, 10.8 Hz, 3H, 3-nitrophenyl-5-H + 3-nitrophenyl-CH=CH + phenyl-CH=CH), 7.45 – 7.35 (m, 3H, phenyl-3-H + phenyl-4-H + phenyl-5-H), 6.95 (d, *J* = 15.9 Hz, 1H, 3-nitrophenyl-CH=CH), 6.75 (d, *J* = 15.9 Hz, 1H, phenyl-CH=CH); ^{13}C NMR (126 MHz, DMSO-*d$_6$*) δ 163.4, 162.8, 148.9, 140.7, 138.3, 137.1, 135.2, 134.5, 131.1, 130.4, 129.5, 128.2, 124.6, 122.9, 122.4, 119.8. HRMS (ESI) $[M+H]^+$ calcd for $C_{18}H_{17}O_4N_3$: 338.1135, found: 338.1134.

***(E)*-*N'*-cinnamoyl-3-(3-hydroxyphenyl)acrylohydrazide (I$_5$)** A white solid, yield 39.0%; ^{1}H NMR (500 MHz, DMSO-*d$_6$*) δ 10.52 (s, 2H, CO-NH-NH-CO), 9.68 (s, 1H, 3-OH-phenyl), 7.59 (dd, *J* = 26.6, 11.5 Hz, 3H, phenyl-2-H + phenyl-6-H + phenyl-CH=CH), 7.49–7.38 (m, 4H, 3-hydroxyphenyl-CH=CH + phenyl-3-H + phenyl-4-H + phenyl-5-H), 7.23 (t, *J* = 7.8 Hz, 1H, 3-hydroxyphenyl-5-H), 7.03-7.02 (d, *J* = 5.0 Hz, 1H, 3-hydroxyphenyl-6-H), 6.98 (s, 1H, 3-hydroxyphenyl-2-H), 6.80 (dd, *J* = 19.7, 8.8 Hz, 2H, phenyl-CH=CH + 3-hydroxyphenyl-4-H), 6.69 (d, *J* = 15.8 Hz, 1H, 3-hydroxyphenyl-CH=CH); ^{13}C NMR (126 MHz, DMSO-*d$_6$*) δ 163.57, 158.27, 140.9, 140.7, 136.4, 135.1, 130.6, 130.4, 129.6, 128.2, 119.9, 119.6, 119.5, 117.6, 114.2.

HRMS (ESI) $[M+H]^+$ calcd for $C_{18}H_{17}O_3N_2$: 309.1234, found: 309.1238.

***(E)-N′*-cinnamoyl-3-(3-methoxyphenyl)acrylohydrazide (I_6)** A white solid, yield 55.0%; ^{1}H NMR (500 MHz, DMSO-*d6*) δ 10.54 (s, 1H, 3-methoxyphenyl-CH=CH-CO-NH), 10.50 (s, 1H, phenyl-CH=CH-CO-NH), 7.61 (d, *J* = 7.1 Hz, 2H, phenyl-2-H + phenyl-6-H), 7.55 (t, *J* = 15.3 Hz, 2H, 3-methoxyphenyl-CH=CH + phenyl-CH=CH), 7.42 (td, *J* = 14.2, 7.0 Hz, 3H, phenyl-3-H + phenyl-4-H + phenyl-5-H), 7.35 (t, *J* = 7.9 Hz, 1H, 3-methoxyphenyl-5-H), 7.21–7.15 (m, 2H, 3-methoxyphenyl-2-H + 3-methoxyphenyl-4-H), 6.98 (dd, *J* = 8.2, 2.0 Hz, 1H, 3-methoxyphenyl-6-H), 6.80 (d, *J* = 4.5 Hz, 1H, phenyl-CH=CH), 6.77 (d, *J* = 4.5 Hz, 1H, 3-methoxyphenyl-CH=CH), 3.79 (s, 3H, 3-OCH_3-phenyl); ^{13}C NMR (126 MHz, DMSO-*d6*) δ 163.5 (d, *J* = 2.1 Hz), 160.1, 140.7, 140.6, 136.6, 135.2, 130.6, 130.4, 129.6, 128.3, 120.5, 120.2, 119.9, 116.1, 113.4, 55.7. HRMS (ESI) $[M+H]^+$ calcd for $C_{19}H_{19}O_3N_2$: 323.1390, found: 323.1391.

***(E)-N′*-cinnamoyl-3-(4-nitrophenyl)acrylohydrazide (I_7)** A yellow solid, yield 62.0%; ^{1}H NMR (500 MHz, DMSO-*d6*) δ 10.70 (s, 1H, 4-nitrophenyl-CH=CH-CO-NH), 10.62 (s, 1H, phenyl-CH=CH-CO-NH), 8.28 (d, *J* = 8.8 Hz, 2H, 4-nitrophenyl-3-H + 4-nitrophenyl-5-H), 7.89 (d, *J* = 8.8 Hz, 2H, 4-nitrophenyl-2-H + 4-nitrophenyl-6-H), 7.68 (d, *J* = 15.9 Hz, 1H, 4-nitrophenyl-CH=CH), 7.62 (d, *J* = 6.9 Hz, 2H, phenyl-2-H + phenyl-6-H), 7.57 (d, *J* = 15.8 Hz, 1H, phenyl-CH=CH), 7.43 (dq, *J* = 14.0, 6.9 Hz, 3H, phenyl-3-H + phenyl-4-H + phenyl-5-H), 6.97 (d, *J* = 15.9 Hz, 1H, 4-nitrophenyl-CH=CH), 6.79 (d, *J* = 15.8 Hz, 1H, phenyl-CH=CH); ^{13}C NMR (126 MHz, DMSO-*d6*) δ 163.4, 162.7, 148.2, 141.7, 140.8, 138.3, 135.1, 130.4, 129.6, 129.3, 128.3, 124.7, 124.2, 119.8. HRMS (ESI) $[M+H]^+$ calcd for $C_{18}H_{16}O_4N_3$: 338.1135, found: 338.1136.

***(E)-3*-(4-chlorophenyl)-*N′*-cinnamoylacrylohydrazide (I_8)** A white solid, yield 60.0%; ^{1}H NMR (500 MHz, DMSO-*d6*) δ 10.53 (s, 2H, CO-NH-NH-CO), 7.63 (dd, *J* = 15.2, 7.7 Hz, 4H, 4-chlorophenyl-2-H + 4-chlorophenyl-3-H + 4-chlorophenyl-5-H + 4-chlorophenyl-6-H), 7.56 (dd, *J* = 15.9, 2.1 Hz, 2H, phenyl-CH=CH + 4-nitrophenyl-CH=CH), 7.50 (d, *J* = 8.3 Hz, 2H, phenyl-2-H + phenyl-6-H), 7.42 (dt, *J* = 17.8, 6.7 Hz, 3H, phenyl-3-H + phenyl-4-H + phenyl-5-H), 6.78 (d, *J* = 15.8 Hz, 2H, 4-nitrophenyl-CH=CH + phenyl-CH=CH); ^{13}C NMR (126 MHz, DMSO-*d6*) δ 163.5,

163.3, 140.7, 139.3, 135.2, 134.8, 134.1, 130.4, 129.9, 129.6, 129.6, 128.2, 120.7, 119.9. HRMS (ESI) $[M+H]^+$ calcd for $C_{18}H_{16}O_2N_2Cl$: 327.0895, found: 327.0898.

***(E)-N'*-cinnamoyl-3-(4-fluorophenyl)acrylohydrazide (I_9)** A white solid, yield 63.0%; ^1H NMR (500 MHz, DMSO-d_6) δ 10.47 (s, 2H, CO-NH-NH-CO), 7.72 – 7.65 (m, 2H, 4-fluorophenyl-2-H + 4-fluorophenyl-6-H), 7.61 (d, J = 6.5 Hz, 2H, 4-fluorophenyl-3-H + 4-fluorophenyl-5-H), 7.55 (d, J = 15.8 Hz, 2H, phenyl-CH=CH + 4-nitrophenyl-CH=CH), 7.48–7.38 (m, 3H, phenyl-3-H + phenyl-4-H + phenyl-5-H), 7.31–7.23 (m, 2H, phenyl-2-H + phenyl-6-H), 6.75 (dd, J = 27.3, 15.9 Hz, 2H, 4-nitrophenyl-CH=CH + phenyl-CH=CH); ^{13}C NMR (126 MHz, DMSO-d_6) δ 163.5, 163.5, 140.6, 139.4, 135.2, 131.8 (d, $^2J_{C\text{-}F}$ = 2.3 Hz), 130.4, 130.4 (d, $^1J_{C\text{-}F}$ = 17.1 Hz), 129.5, 128.2, 120.0, 119.9, 116.6, 116.4. HRMS (ESI) $[M+H]^+$ calcd for $C_{18}H_{16}O_2N_2F$: 311.1190, found: 311.1189.

***(E)-N'*-cinnamoyl-3-(p-tolyl)acrylohydrazide (I_{10})** A white solid, yield 58.0%; ^1H NMR (500 MHz, DMSO-d_6) δ 10.48 (s, 1H, phenyl-CH=CH-CO-NH), 10.45 (s, 1H, 4-CH_3-phenyl-CH=CH-CO-NH), 7.63–7.56 (m, 3H, phenyl-2-H + phenyl-6-H + phenyl-CH=CH), 7.54 (d, J = 2.2 Hz, 1H, 4-CH_3-phenyl-CH=CH), 7.52–7.48 (m, 2H, 4-CH_3-phenyl-3-H + 4-CH_3-phenyl-5-H), 7.47–7.37 (m, 3H, phenyl-3-H + phenyl-4-H + phenyl-5-H), 7.25 (d, J = 7.8 Hz, 2H, 4-CH_3-phenyl-2-H + 4-CH_3-phenyl-6-H), 6.78 (d, J = 15.9 Hz, 1H, phenyl-CH=CH), 6.72 (d, J = 15.8 Hz, 1H, 4-CH_3-phenyl-CH=CH), 2.33 (s, 3H, 4-CH_3-phenyl); ^{13}C NMR (126 MHz, DMSO-d_6) δ 163.8, 163.6, 140.6, 140.2, 135.2, 132.4, 130.3, 130.1, 129.5, 128.2, 119.9, 118.8, 21.5. HRMS (ESI) $[M+H]^+$ calcd for $C_{19}H_{19}O_2N_2$: 307.1441, found: 307.1437.

***(E)-N'*-cinnamoyl-3-(4-isopropylphenyl)acrylohydrazide (I_{11})** A white solid, yield 54.0%; ^1H NMR (500 MHz, DMSO-d_6) δ 10.44 (d, J = 7.9 Hz, 2H, CO-NH-NH-CO), 7.65–7.49 (m, 6H, 4-isoprophenyl-3-H + 4-isoprophenyl-5-H + 4-isoprophenyl-3-H + 4-isoprophenyl-5-H + phenyl-CH=CH + 4-isopropylphenyl-CH=CH), 7.42 (dd, J = 9.4, 6.7 Hz, 3H, phenyl-3-H + phenyl-4-H + phenyl-5-H), 7.31 (d, J = 5.3 Hz, 2H, phenyl-2-H + phenyl-6-H), 6.78 (dd, J = 16.0, 3.1 Hz, 1H, phenyl-CH=CH), 6.73 (dd, J = 15.8, 3.1 Hz, 1H, 4-isopropylphenyl-CH=CH), 2.97–2.85 (m, 1H, $CH(CH_3)_2$), 1.23–1.19 (m, 6H, $CH(CH_3)_2$); ^{13}C NMR (126 MHz, $CDCl_3$) δ 168.5, 168.4, 155.7, 145.4, 140.0, 137.6,

135.1, 134.3, 133.1, 133.1, 133.0, 132.3, 124.7, 123.7, 38.6, 28.9. HRMS (ESI) $[M+H]^+$ calcd for $C_{21}H_{23}O_2N_2$: 335.1754, found: 335.1754.

***(E)-N'*-cinnamoyl-3-(4-methoxyphenyl)acrylohydrazide (I_{12})** A white solid, yield 53.0%; ^{1}H NMR (500 MHz, DMSO-*d6*) δ 10.47 (s, 1H, phenyl-CH=CH-CO-NH), 10.40 (s, 1H, 4-CH_3O-phenyl-CH=CH-CO-NH), 7.61 (d, *J* = 7.1 Hz, 2H, 4-CH_3O-phenyl-2-H + 4-CH_3O-phenyl-6-H), 7.59–7.53 (m, 3H, phenyl-CH=CH + 4-CH_3O-phenyl-3-H + 4-CH_3O-phenyl-5-H), 7.51 (d, *J* = 15.8 Hz, 1H, 4-CH_3O-phenyl-CH=CH), 7.47–7.36 (m, 3H, phenyl-3-H + phenyl-4-H + phenyl-5-H), 7.00 (d, *J* = 8.7 Hz, 2H, phenyl-2-H + phenyl-6-H), 6.78 (d, *J* = 15.9 Hz, 1H, 4-CH_3O-phenyl-CH=CH), 6.63 (d, *J* = 15.8 Hz, 1H, phenyl-CH=CH), 3.79 (s, 3H,4-CH_3O-phenyl); ^{13}C NMR (126 MHz, DMSO-*d6*) δ 163.9, 163.6, 161.1, 140.6, 140.4, 135.2, 130.3, 129.9, 129.6, 128.2, 127.7, 120.0, 117.3, 115.0, 55.8. HRMS (ESI) $[M+H]^+$ calcd for $C_{19}H_{19}O_3N_2$: 323.1390, found: 323.1394.

***(E)-N'*-cinnamoyl-3-(4-ethoxyphenyl)acrylohydrazide (I_{13})** A white solid, yield 60.0%; ^{1}H NMR (500 MHz, DMSO-*d6*) δ 10.42 (s, 1H, phenyl-CH=CH-CO-NH), 10.35 (s, 1H, 4-C_2H_5-phenyl-CH=CH-CO-NH), 7.64–7.36 (m, 9H, phenyl-CH=CH + 4-C_2H_5-phenyl-CH=CH + 4-C_2H_5-phenyl-2-H + 4-C_2H_5-phenyl-4-H + phenyl-2-H + phenyl-3-H + phenyl-4-H + phenyl-5-H + phenyl-6-H), 6.98 (d, *J* = 5.8 Hz, 2H, 4-C_2H_5-phenyl-3-H + 4-C_2H_5-phenyl-5-H), 6.78 (d, *J* = 15.2 Hz, 1H, phenyl-CH=CH), 6.62 (d, *J* = 15.5 Hz, 1H, 4-C_2H_5-phenyl-CH=CH), 4.07 (d, *J* = 4.1 Hz, 2H, CH_2-CH_3), 1.33 (s, 3H, CH_2-CH_3); ^{13}C NMR (126 MHz, DMSO-*d6*) δ 164.0, 163.6, 160.4, 140.6, 140.4, 135.2, 130.3, 129.9, 129.5, 128.2, 127.6, 120.0, 117.3, 115.4, 63.8, 15.1. HRMS (ESI) $[M+H]^+$ calcd for $C_{20}H_{21}O_3N_2$: 337.1547, found: 337.1547.

***(E)-N'*-cinnamoyl-3-(4-(dimethylamino)phenyl)acrylohydrazide (I_{14})** A yellow solid, yield 43.0%; ^{1}H NMR (500 MHz, DMSO-*d6*) δ 10.39 (s, 1H, phenyl-CH=CH-CO-NH), 10.23 (s, 1H, 4-N(CH_3)$_2$-phenyl-CH=CH-CO-NH), 7.61 (d, *J* = 7.0 Hz, 2H, phenyl-2-H + phenyl-6-H), 7.54 (d, *J* = 15.8 Hz, 1H, phenyl-CH=CH), 7.47–7.37 (m, 6H, 4-N(CH_3)$_2$-phenyl-CH=CH + phenyl-3-H + phenyl-4-H + phenyl-5-H + 4-N(CH_3)$_2$-phenyl-2-H + 4-N(CH_3)$_2$-phenyl-6-H), 6.75 (q, *J* = 17.8, 12.3 Hz, 3H, phenyl-CH=CH + 4-N(CH_3)$_2$-phenyl-3-H + 4-N(CH_3)$_2$-phenyl-5-H), 6.49 (d, *J* = 15.8 Hz, 1H,

4-N(CH_3)$_2$-phenyl-CH=CH), 2.97 (s, 6H, N(CH_3)$_2$); ^{13}C NMR (126 MHz, DMSO-d_6) δ 164.5, 163.6, 151.9, 141.2, 140.4, 135.2, 130.3, 129.6, 129.5, 128.2, 122.6, 120.1, 114.1, 112.5. HRMS (ESI) $[M+H]^+$ calcd for $C_{20}H_{22}O_2N_3$: 336.1707, found: 336.1708.

***(E)-N′*-cinnamoyl-3-(4-hydroxy-3-methoxyphenyl)acrylohydrazide (I_{15})** A white solid, yield 30.0%; ^{1}H NMR (500 MHz, DMSO-d_6) δ 10.46 (d, J = 2.9 Hz, 1H, phenyl-CH=CH-CO-NH), 10.32 (d, J = 2.9 Hz, 1H, 4-OH-3-CH_3O-phenyl-CH=CH-CO-NH), 9.54 (s, 1H, 4-OH-3-CH_3O-phenyl), 7.61 (d, J = 7.2 Hz, 2H, phenyl-2-H + phenyl-6-H), 7.55 (d, J = 15.9 Hz, 1H, phenyl-CH=CH), 7.48–7.37 (m, 4H, phenyl-3-H + phenyl-4-H + phenyl-5-H + 4-OH-3-CH_3O-phenyl-CH=CH), 7.18 (s, 1H, 4-OH-3-CH_3O-phenyl-2-H), 7.06 (d, J = 8.1 Hz, 1H, 4-OH-3-CH_3O-phenyl-5-H), 6.82 (d, J = 8.1 Hz, 1H, 4-OH-3-CH_3O-phenyl-6-H), 6.77 (d, J = 15.8 Hz, 1H, phenyl-CH=CH), 6.60 (d, J = 15.8 Hz, 1H, 4-OH-3-CH_3O-phenyl-CH=CH), 3.82 (s, 3H, 4-OH_3-CH_3O-phenyl); ^{13}C NMR (126 MHz, DMSO-d_6) δ 164.1, 163.5, 149.2, 148.4, 141.1, 140.5, 135.2, 130.3, 129.6, 128.2, 126.7, 122.2, 120.0, 116.5, 116.2, 111.6, 56.0. HRMS (ESI) $[M+H]^+$ calcd for $C_{19}H_{19}O_4N_2$: 339.1339, found: 339.1335.

***(E)-N′*-cinnamoyl-3-cyclopropylacrylohydrazide (I_{16})** A white solid, yield 28.0%; ^{1}H NMR (500 MHz, DMSO-d_6) δ 10.19 (s, 1H, phenyl-CH=CH-CO-NH), 10.06 (s, 1H, 3-cyclopropylphenyl-CH=CH-CO-NH), 7.56 (d, J = 6.5 Hz, 2H, phenyl-2-H + phenyl-6-H), 7.49 (dd, J = 15.6, 2.3 Hz, 1H, phenyl-CH=CH), 7.44–7.32 (m, 3H, phenyl-3-H + phenyl-4-H + phenyl-5-H), 6.70 (dd, J = 15.9, 2.9 Hz, 1H, phenyl-CH=CH), 6.28–6.16 (m, 1H, 3-cyclopropylphenyl-CH=CH), 6.06 (dd, J = 15.2, 2.8 Hz, 1H, 3-cyclopropylphenyl-CH=CH), 1.62–1.50 (m, 1H, 3-cyclopropyl-1-H), 0.91–0.74 (m, 2H, 3-cyclopropyl-2-H), 0.62 – 0.52 (m, 2H, 3-cyclopropyl-3-H); ^{13}C NMR (126 MHz, $CDCl_3$) δ 168.5, 154.7, 145.3, 139.9, 135.1, 134.3, 133.0, 124.8, 124.0, 19.3, 13.3. HRMS (ESI) $[M+H]^+$ calcd for $C_{15}H_{16}O_2N_2$: 257.1285, found: 257.1283.

***(E)-N′*-cinnamoyl-3-(furan-2-yl)acrylohydrazide (I_{17})** A yellow solid, yield 36.0%; ^{1}H NMR (500 MHz, DMSO-d_6) δ 10.51 (s, 1H, phenyl-CH=CH-CO-NH), 10.47 (s, 1H, furan-2-yl-CH=CH-CO-NH), 7.82 (s, 1H, furan-2-yl-5-H), 7.61 (d, J = 7.2 Hz, 2H, phenyl-2-H + phenyl-6-H), 7.55 (dd, J = 15.8, 1.7 Hz, 1H, phenyl-CH=CH), 7.43 (dd, J = 15.1, 9.0 Hz, 3H, furan-2-yl-CH=CH + phenyl-3-H + phenyl-5-H), 7.39 – 7.34 (m,

1H, phenyl-4-H), 6.84 (s, 1H, furan-2-yl-3-H), 6.77 (dd, *J* = 15.9, 2.2 Hz, 1H, phenyl-CH=CH), 6.61 (d, *J* = 1.8 Hz, 1H, furan-2-yl-4-H), 6.54 (dd, *J* = 15.6, 2.0 Hz, 1H, furan-2-yl-CH=CH); ^{13}C NMR (126 MHz, DMSO-d_6) δ 163.6, 163.5, 151.3, 145.7, 140.6, 135.2, 130.4, 129.5, 128.2, 127.9, 119.9, 116.9, 115.2, 113.1. HRMS (ESI) $[M+H]^+$ calcd for $C_{16}H_{15}O_3N_2$: 283.1077, found: 283.1074.

***(E)-N′*-cinnamoyl-3-(thiophen-2-yl)acrylohydrazide (I_{18})** A yellow solid, yield 42.0%; ^{1}H NMR (500 MHz, DMSO-d_6) δ 10.44 (s, 2H, CO-NH-NH-CO), 7.71 (dd, *J* = 15.3, 2.5 Hz, 1H, thiophen-2-yl-CH=CH), 7.66–7.59 (m, 3H, phenyl-2-H + phenyl-6-H + thiophen-2-yl-5-H), 7.55 (dd, *J* = 15.9, 2.9 Hz, 1H, phenyl-CH=CH), 7.47–7.37 (m, 4H, phenyl-3-H + phenyl-4-H + phenyl-5-H + thiophen-2-yl-3-H), 7.19–7.09 (m, 1H, thiophen-2-yl-4-H), 6.77 (dd, *J* = 15.9, 3.2 Hz, 1H, phenyl-CH=CH), 6.53 (dd, *J* = 15.5, 3.1 Hz, 1H, thiophen-2-yl-CH=CH); ^{13}C NMR (126 MHz, DMSO-d_6) δ 163.6, 163.4, 140.6, 140.2, 135.2, 133.7, 131.8, 130.3, 129.5, 129.0, 128.9, 128.2, 119.9, 118.5. HRMS (ESI) $[M+H]^+$ calcd for $C_{16}H_{15}O_2N_2S$: 299.0849, found: 299.0842.

***(E)-N′*-cinnamoyl-3-(4-methylthiazol-5-yl)acrylohydrazide (I_{19})** A yellow solid, yield 52.0%; ^{1}H NMR (500 MHz, DMSO-d_6) δ 10.48 (s, 2H, CO-NH-NH-CO), 9.01 (s, 1H,), 7.64 (d, *J* = 15.4 Hz, 1H, 4-methylthiazol-5-yl-CH=CH), 7.58 (d, *J* = 6.8 Hz, 2H, phenyl-2-H + phenyl-6-H), 7.52 (d, *J* = 15.8 Hz, 1H, phenyl-CH=CH), 7.45–7.33 (m, 3H, phenyl-3-H + phenyl-4-H + phenyl-5-H), 6.74 (d, *J* = 15.8 Hz, 1H, phenyl-CH=CH), 6.45 (d, *J* = 15.4 Hz, 1H, 4-methylthiazol-5-yl-CH=CH), 2.46 (s, 3H, CH_3); ^{13}C NMR (126 MHz, DMSO-d_6) δ 163.5, 162.9, 155.0, 154.3, 140.7, 135.2, 130.4, 130.0, 129.5, 128.8, 128.2, 121.7, 119.8, 15.8. HRMS (ESI) $[M+H]^+$ calcd for $C_{16}H_{16}O_2N_3S$: 314.0958, found: 314.0954.

***(E)-N′*-cinnamoyl-3-cyclohexylacrylohydrazide (I_{20})** A white solid, yield 48.0%; ^{1}H NMR (500 MHz, DMSO-d_6) δ 10.29 (d, *J* = 2.9 Hz, 1H, phenyl-CH=CH-CO-NH), 10.21 (d, *J* = 2.9 Hz, 1H, 3-cyclohexyl-CH=CH-CO-NH), 7.60 (d, *J* = 6.9 Hz, 2H, phenyl-2-H + phenyl-6-H), 7.53 (d, *J* = 15.8 Hz, 1H, phenyl-CH=CH), 7.41 (dq, *J* = 14.1, 6.9 Hz, 3H, phenyl-3-H + phenyl-4-H + phenyl-5-H), 6.81–6.63 (m, 2H, phenyl-CH=CH + 3-cyclohexyl-CH=CH), 5.98 (dd, *J* = 15.6, 1.1 Hz, 1H, 3-cyclohexyl-CH=CH), 2.19–2.06 (m, 1H, 3-cyclohexyl-1-H), 1.75–1.58 (m, 5H, 3-cyclohexyl-3-H

+ 3-cyclohexyl-4-H + 3-cyclohexyl-5-H), 1.34–1.04 (m, 5H, 3-cyclohexyl-2-H + 3-cyclohexyl-4-H + 3-cyclohexyl-6-H); ^{13}C NMR (126 MHz, DMSO-*d6*) δ 164.0, 163.8, 149.9, 140.5, 135.2, 130.3, 129.5, 128.2, 120.0, 119.9, 32.0, 26.1, 25.8. HRMS (ESI) $[M+H]^+$ calcd for $C_{18}H_{23}O_2N_2$: 299.1754, found: 299.1755.

***(E)-N'*-cinnamoyl-3-(cyclohex-3-en-1-yl)acrylohydrazide (I_{21})** A white solid, yield 49.0%; ^{1}H NMR (500 MHz, DMSO-*d6*) δ 10.24 (s, 1H, phenyl-CH=CH-CO-NH), 10.18 (s, 1H, cyclohex-3-en-1-yl-CH=CH-CO-NH), 7.60–7.54 (m, 2H, phenyl-2-H + phenyl-6-H), 7.50 (dd, *J* = 15.8, 3.1 Hz, 1H, phenyl-CH=CH), 7.38 (dd, *J* = 11.3, 7.2 Hz, 3H, phenyl-3-H + phenyl-4-H + phenyl-5-H), 6.79–6.62 (m, 2H, phenyl-CH=CH + cyclohex-3-en-1-yl-CH=CH), 6.01 (d, *J* = 15.5 Hz, 1H, cyclohex-3-en-1-yl-CH=CH), 5.65 (s, 2H, cyclohex-3-en-1-yl-3-H + cyclohex-3-en-1-yl-4-H), 2.38 (s, 1H, cyclohex-3-en-1-yl-1-H), 2.16–1.99 (m, 3H, cyclohex-3-en-1-yl-2-H + cyclohex-3-en-1-yl-5-H), 1.90–1.71 (m, 2H, cyclohex-3-en-1-yl-6-H), 1.37 (dd, *J* = 12.6, 6.8 Hz, 1H, cyclohex-3-en-1-yl-2-H). ^{13}C NMR (101 MHz, DMSO-*d6*) δ 163.8, 163.8, 148.9, 140.5, 135.1, 130.3, 129.5, 128.1, 127.3, 125.9, 120.5, 119.9, 36.0, 30.3, 27.7, 24.5. HRMS (ESI) $[M+H]^+$ calcd for $C_{18}H_{21}O_2N_2$: 297.1598, found: 297.1596.

***(E)-N'*-cinnamoyl-3-(pyridin-3-yl)acrylohydrazide (I_{22})** A yellow solid, yield 63.0%; ^{1}H NMR (500 MHz, DMSO-*d6*) δ 10.61 (s, 2H, CO-NH-NH-CO), 8.81 (s, 1H, pyridin-2-H), 8.62–8.54 (m, 1H, pyridin-6-H), 8.03 (d, *J* = 6.2 Hz, 1H, pyridin-4-H), 7.62-7.55 (m, *J* = 19.9, 14.0 Hz, 4H, phenyl-2-H + phenyl-6-H + pyridin-3-yl-CH=CH + pyridin-3-H), 7.51–7.35 (m, 4H, phenyl-3-H + phenyl-4-H + phenyl-5-H + phenyl-CH=CH), 6.89 (dd, *J* = 15.9, 2.5 Hz, 1H, pyridin-3-yl-CH=CH), 6.79 (dd, *J* = 15.8, 2.5 Hz, 1H, phenyl-CH=CH); ^{13}C NMR (126 MHz, DMSO-*d6*) δ 163.5, 163.1, 151.0, 149.9, 140.8, 137.4, 135.1, 134.6, 131.0, 130.4, 129.6, 128.3, 124.6, 121.8, 119.8. HRMS (ESI) $[M+H]^+$ calcd for $C_{17}H_{16}O_2N_3$: 294.1237, found: 294.1238.

(*E*)-3-(3-methoxyphenyl)-*N'*-((*E*)-3-(4-nitrophenyl)acryloyl)acrylohydrazide (I_{23}) A yellow solid, yield 61%; ^{1}H NMR (400 MHz, DMSO-*d6*) δ 10.66 (s, 1H, 4-NO_2-phenyl-CH=CH-CO-NH), 10.58 (s, 1H, 3-OCH_3-phenyl-CH=CH-CO-NH), 8.28 (d, *J* = 8.6 Hz, 2H, 4-NO_2-phenyl-3-H + 4-NO_2-phenyl-5-H), 7.88 (d, *J* = 8.6 Hz, 2H, 4-NO_2-phenyl-2-H + 4-NO_2-phenyl-6-H), 7.68 (dd, *J* = 15.8, 3.7 Hz, 1H, 4-NO_2-phenyl-

CH=CH), 7.53 (d, J = 15.8 Hz, 1H, 3-OCH_3-phenyl-CH=CH), 7.35 (t, J = 7.9 Hz, 1H, 3-OCH_3-phenyl-2-H), 7.23–7.14 (m, 2H, 3-OCH_3-phenyl-5-H + 3-OCH_3-phenyl-6-H), 6.97 (dd, J = 11.3, 4.7 Hz, 2H, + 4-NO_2-phenyl-CH=CH + 3-OCH_3-phenyl-3-H), 6.79 (d, J = 15.8 Hz, 1H, 4-NO_2-phenyl-CH=CH), 3.80 (s, 3H, 3-CH_3O-Phenyl); ^{13}C NMR (101 MHz, DMSO-d_6) δ 163.3, 162.6, 160.1, 148.2, 141.7, 140.6, 138.3, 136.5, 130.5, 129.2, 124.6, 124.1, 120.5, 120.1, 116.1, 113.4, 55.6. HRMS (ESI) $[M+H]^+$ calcd for $C_{19}H_{18}O_5N_3$: 368.1241, found: 368.1233.

(*E*)-3-(4-methoxyphenyl)-*N*'-((*E*)-3-(4-nitrophenyl)acryloyl)acrylohydrazide (I$_{24}$) A yellow solid, yield 53%; ^{1}H NMR (400 MHz, DMSO-d_6) δ 10.63 (s, 1H, 4-NO_2-phenyl-CH=CH-CO-NH), 10.48 (s, 1H, 4-OCH_3-phenyl-CH=CH-CO-NH), 8.28 (d, J = 8.8 Hz, 2H, 4-NO_2-phenyl-3-H + 4-NO_2-phenyl-5-H), 7.88 (d, J = 8.8 Hz, 2H, 4-NO_2-phenyl-2-H + 4-NO_2-phenyl-6-H), 7.67 (d, J = 15.9 Hz, 1H, 4-NO_2-phenyl-CH=CH), 7.56 (d, J = 8.7 Hz, 2H, 4-OCH_3-phenyl-3-H + 4-OCH_3-phenyl-5-H), 7.51 (d, J = 15.8 Hz, 1H, 4-OCH_3-phenyl-CH=CH), 6.98 (t, J = 12.7 Hz, 3H, 4OCH_3-phenyl-CH=CH + 4-OCH_3-phenyl-2-H + 4-OCH_3-phenyl-6-H), 6.63 (d, J = 15.8 Hz, 1H, 4-NO_2-phenyl-CH=CH), 3.80 (s, 3H, 4-CH_3O-Phenyl); ^{13}C NMR (101 MHz, DMSO-d_6) 163.8, 162.7, 161.1, 148.2, 141.7, 140.4, 138.1, 129.8, 129.2, 127.7, 124.6, 124.2, 117.1, 114.9, 55.8. HRMS (ESI) $[M+H]^+$ calcd for $C_{19}H_{18}O_5N_3$: 368.1241, found: 368.1232.

(*E*)-3-(3-methoxyphenyl)-*N*'-((*E*)-3-(3-methoxyphenyl)acryloyl)acrylohydrazide (I$_{25}$) A white solid, yield 50%; ^{1}H NMR (500 MHz, DMSO-d_6) δ 10.44 (d, J = 3.1 Hz, 1H, 4-OCH_3-phenyl-CH=CH-CO-NH), 10.41 (d, J = 3.0 Hz, 1H, 3-OCH_3-phenyl-CH=CH-CO-NH), 7.59–7.46 (m, 4H, 3-OCH_3-phenyl-CH=CH + 4-OCH_3-phenyl-CH=CH + 4-OCH_3-phenyl-3-H + 4-OCH_3-phenyl-5-H), 7.35 (t, J = 7.9 Hz, 1H, 3-OCH_3-phenyl-6-H), 7.18 (dd, J = 10.1, 4.9 Hz, 2H, 4-OCH_3-phenyl-2-H + 4-OCH_3-phenyl-4-H), 6.98 (dd, J = 13.6, 5.5 Hz, 3H, 3-OCH_3-phenyl-2-H + 3-OCH_3-phenyl-4-H + 3-OCH_3-phenyl-5-H), 6.78 (d, J = 15.9 Hz, 1H, 4-OCH_3-phenyl-CH=CH), 6.62 (d, J = 15.9 Hz, 1H, 3-OCH_3-phenyl-CH=CH), 3.80 (s, 6H, OCH_3). ^{13}C NMR (126 MHz, DMSO-d_6) δ 163.9, 163.5, 161.1, 160.1, 140.5, 140.4, 136.6, 130.6, 129.9, 127.7, 120.5, 120.3, 117.3, 116.1, 115.0, 113.4, 55.8, 55.7. HRMS (ESI) $[M+H]^+$ calcd for $C_{20}H_{21}O_4N_2$: 353.1495, found: 353.1482.

(*E*)-3-(3-methoxyphenyl)-*N*'-((*E*)-3-(4-methoxyphenyl)acryloyl)acrylohydrazide (I_{26}) A white solid, yield 43%; ^{1}H NMR (500 MHz, DMSO-d_6) δ 10.52 (s, 2H, CO-NH-NH-CO), 7.52 (s, 1H, 3-OCH_3-phenyl-2-H), 7.49 (s, 1H, 3-OCH_3-phenyl-2-H), 7.34 (t, J = 7.9 Hz, 2H, 3-OCH_3-phenyl-5-H), 7.17 (dd, J = 10.7, 4.9 Hz, 4H, 3-OCH_3-phenyl-4-H + 3-OCH_3-phenyl-6-H), 6.98 (d, J = 10.0 Hz, 1H, 3-OCH_3-phenyl-CH=CH), 6.96 (d, J =10.0 Hz, 1H, 3-OCH_3-phenyl-CH=CH), 6.84 (s, 1H, 3-OCH_3-phenyl-CH=CH), 6.81 (s, 1H, 3-OCH_3-phenyl-CH=CH), 3.78 (d, J = 4.0 Hz, 6H, OCH_3). ^{13}C NMR (126 MHz, DMSO-d_6) δ 163.7, 160.1, 140.4, 136.6, 130.6, 120.5, 120.4, 116.1, 113.4, 55.7. HRMS (ESI) $[M+H]^+$ calcd for $C_{20}H_{21}O_4N_2$: 353.1496, found: 353.1483.

5. ^{1}H NMR, ^{13}C NMR, ^{19}F NMR and HRMS spectra for target compounds

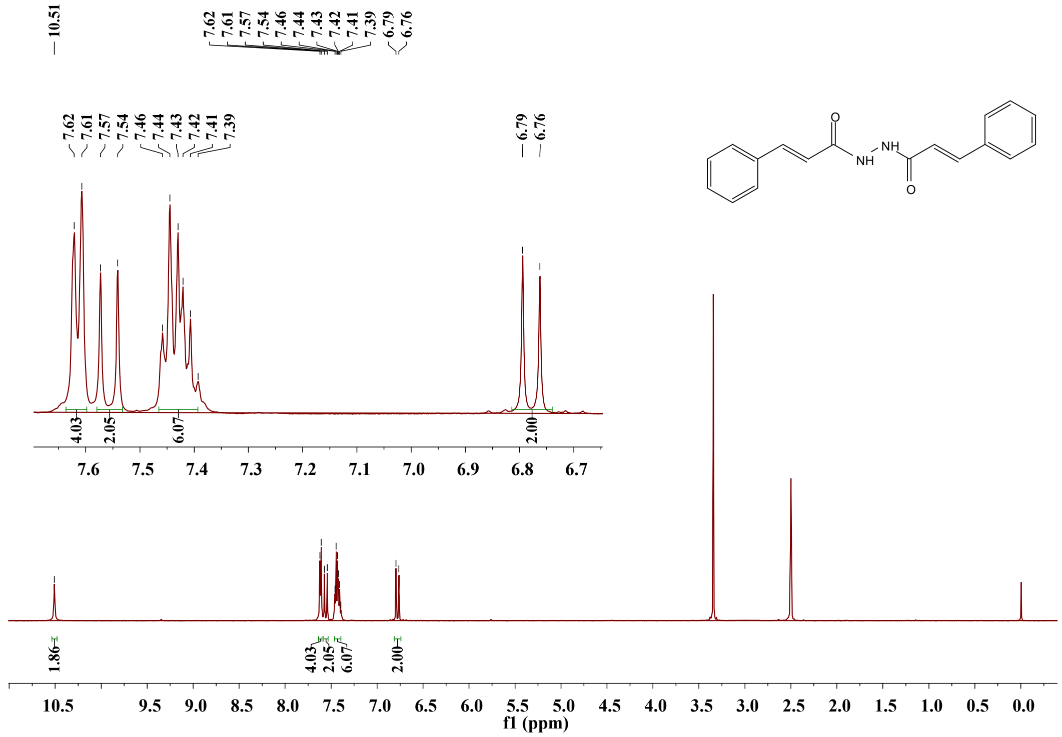

Figure S1. ^{1}H NMR Spectrum (DMSO-d_6, 500 MHz) of I_1.

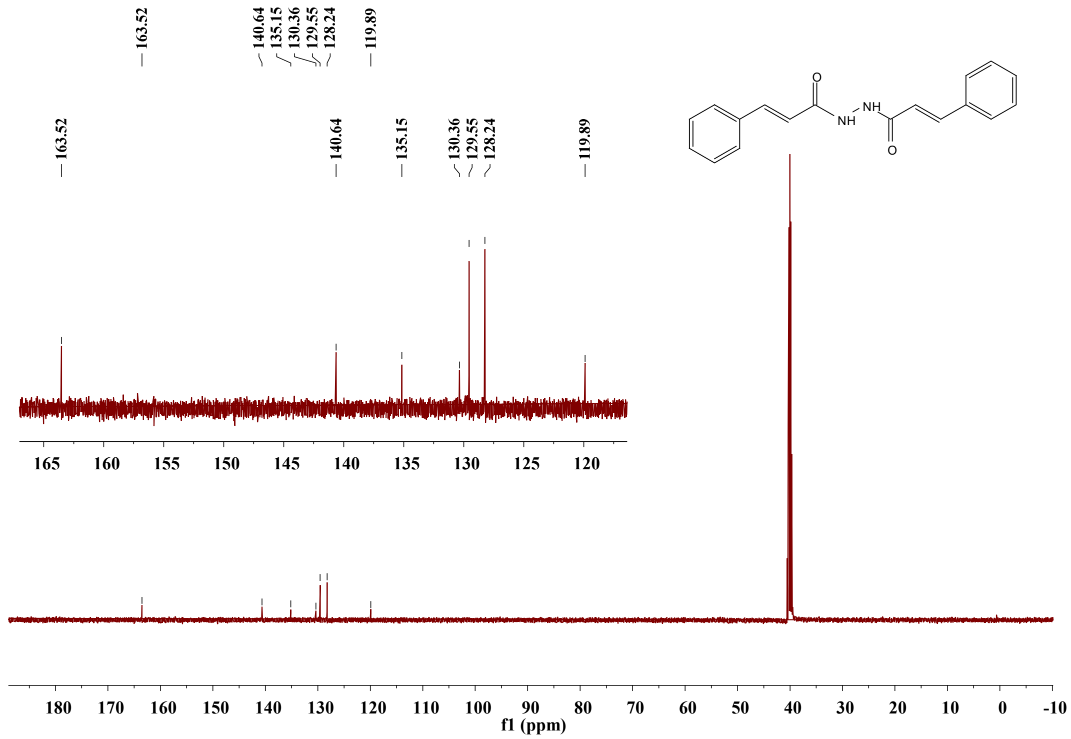

Figure S2. ^{13}C NMR Spectrum (DMSO-d_6, 126 MHz) of I_1.

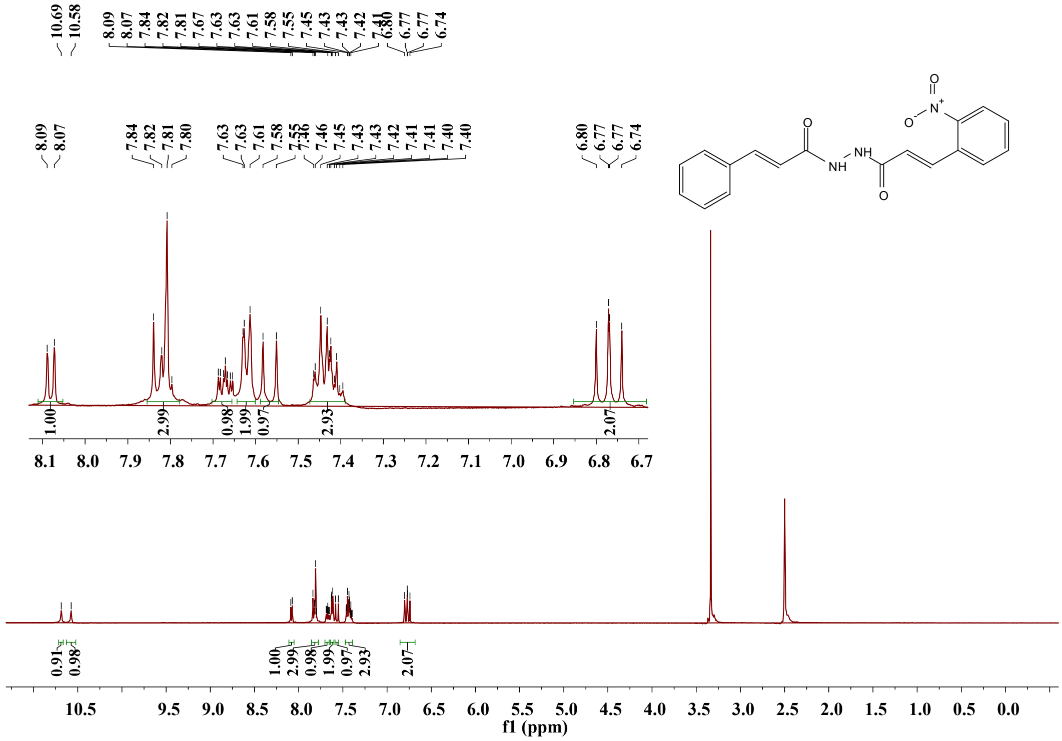

Figure S3. ^{1}H NMR Spectrum (DMSO-d_6, 500 MHz) of I_2.

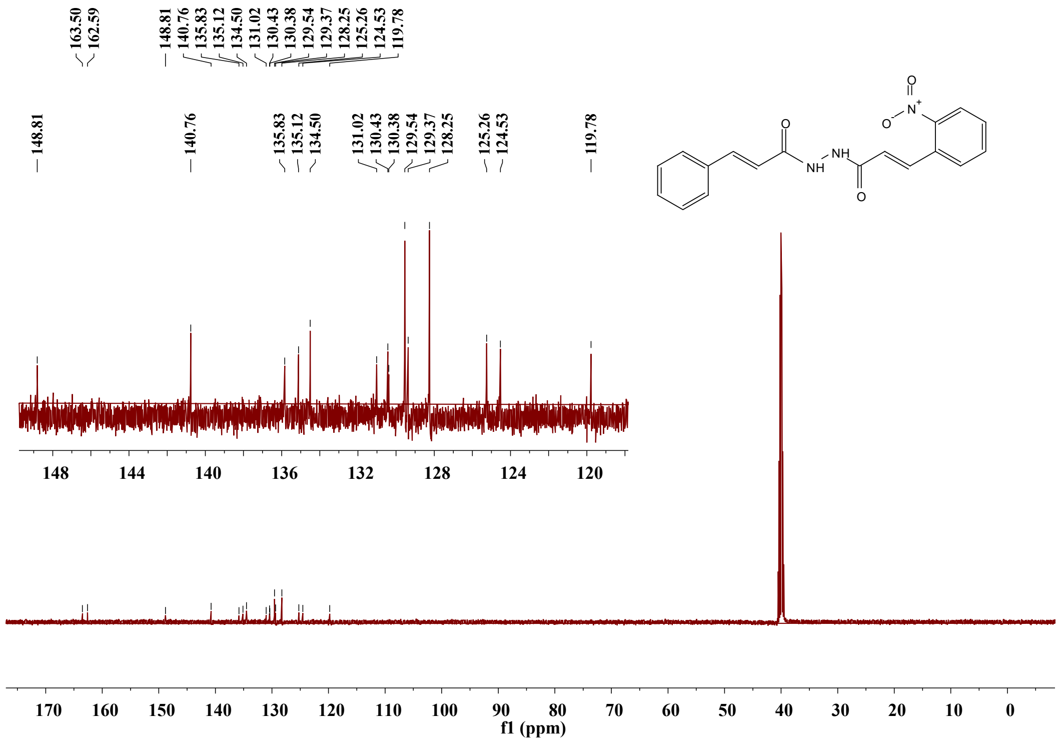

Figure S4. ^{13}C NMR Spectrum (DMSO-d_6, 126 MHz) of I_2.

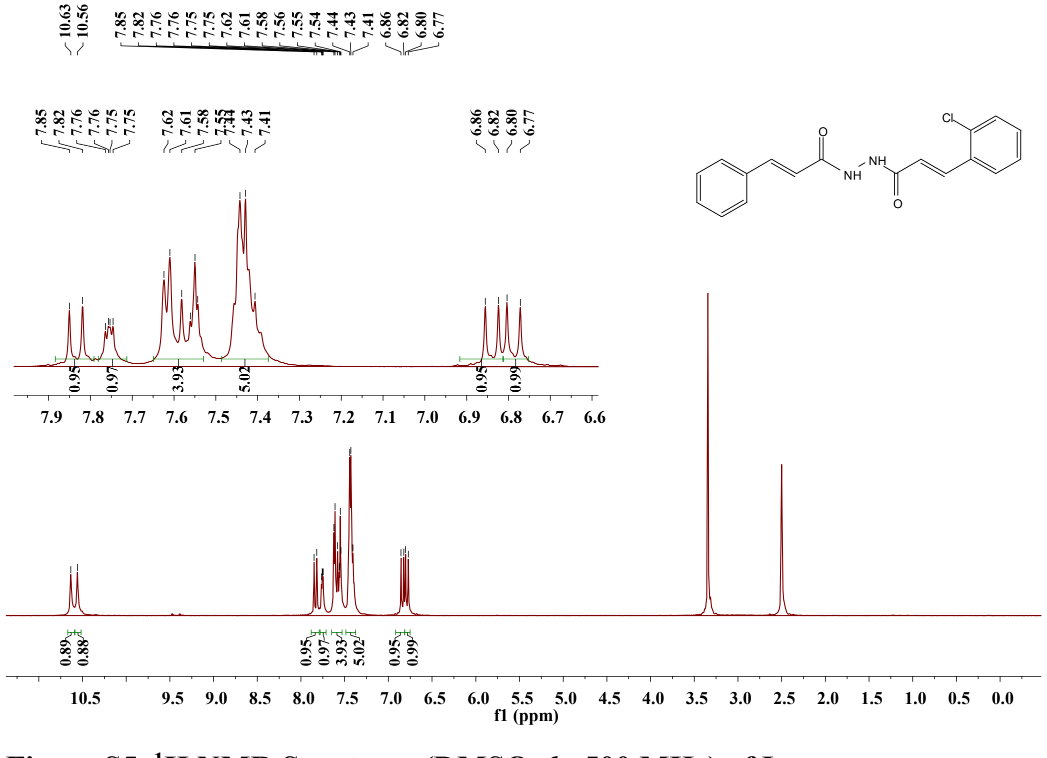

Figure S5. ^{1}H NMR Spectrum (DMSO-d_6, 500 MHz) of I_3.

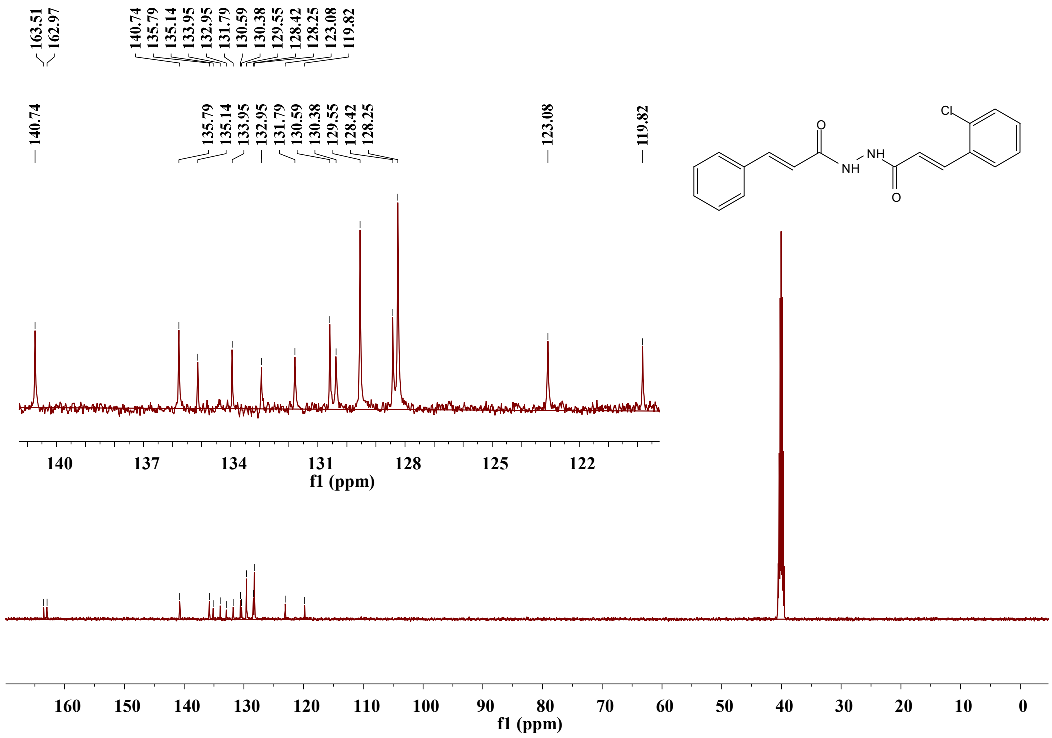

Figure S6. ^{13}C NMR Spectrum (DMSO-d_6, 126 MHz) of I_3.

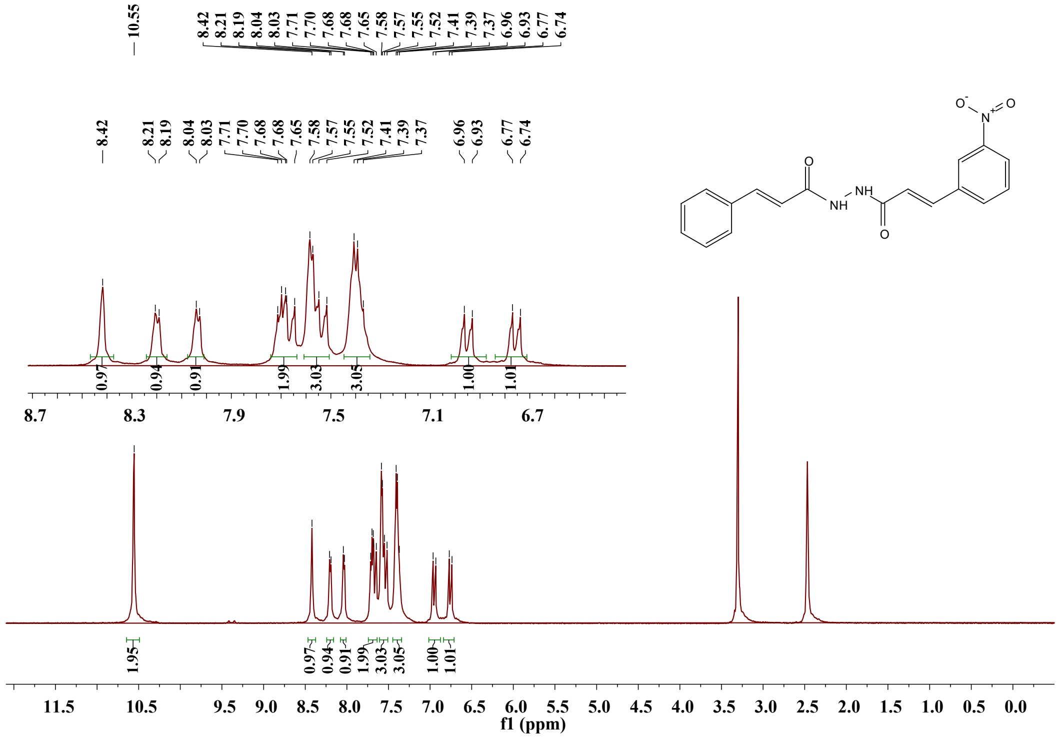

Figure S7. ^{1}H NMR Spectrum (DMSO-d_6, 500 MHz) of I_4.

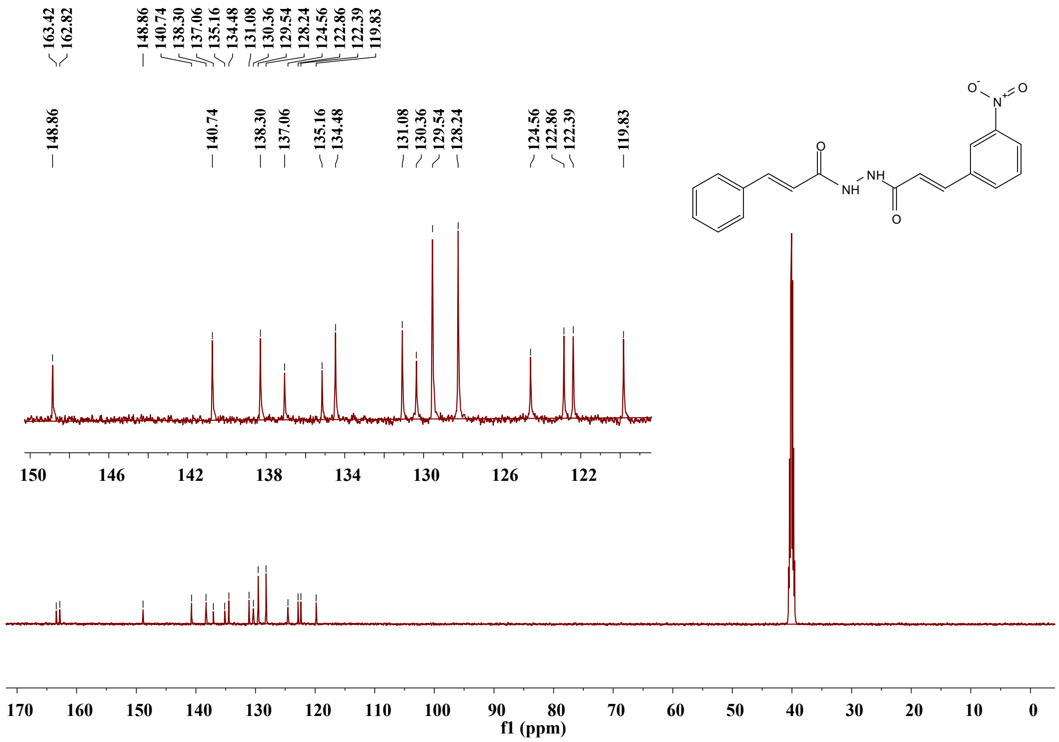

Figure S8. ^{13}C **NMR Spectrum (DMSO-*d*$_6$, 126 MHz) of I$_4$.**

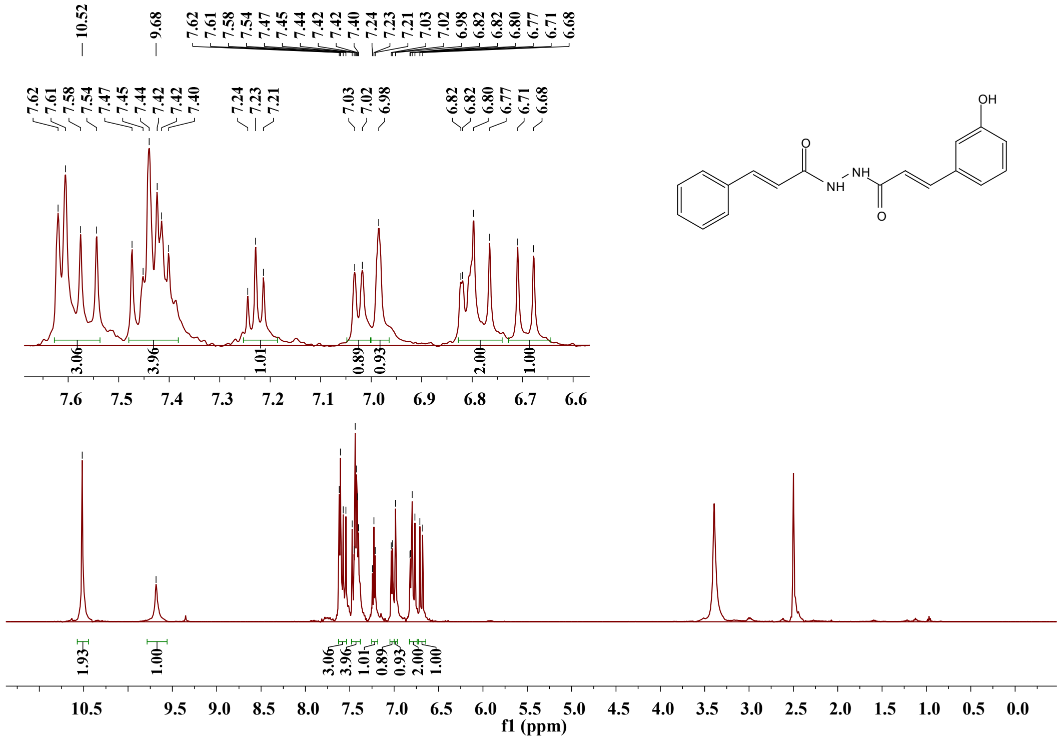

Figure S9. ^{1}H **NMR Spectrum (DMSO-*d*$_6$, 500 MHz) of I$_5$.**

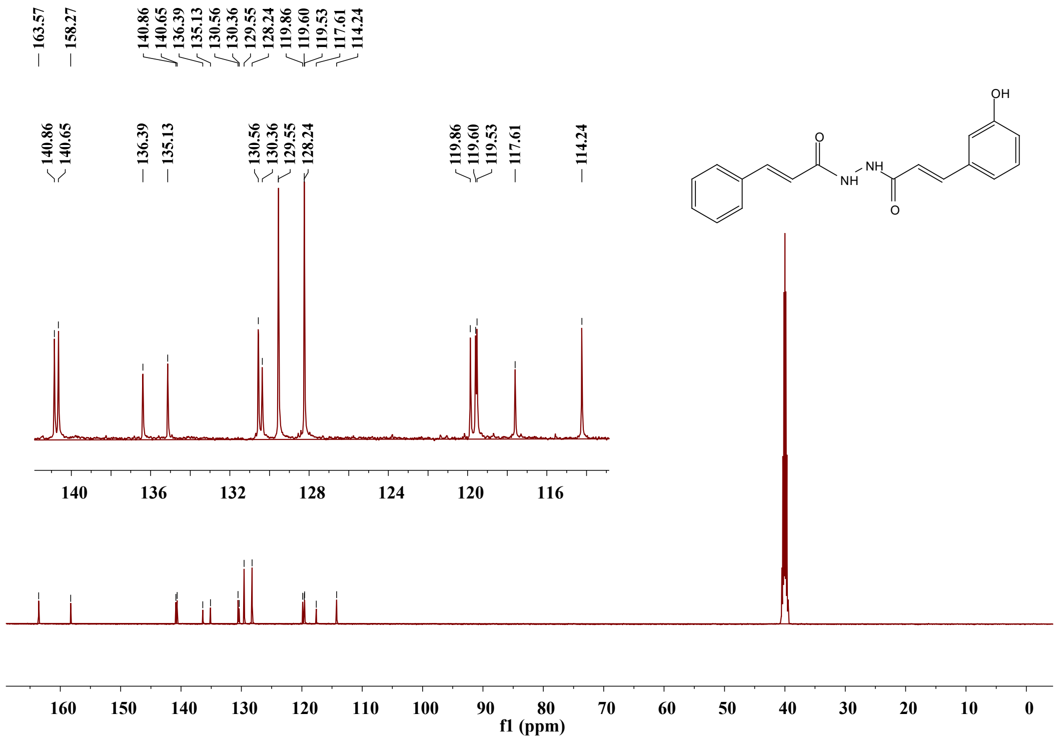

Figure S10. ^{13}C NMR Spectrum (DMSO-d_6, 126 MHz) of I_5.

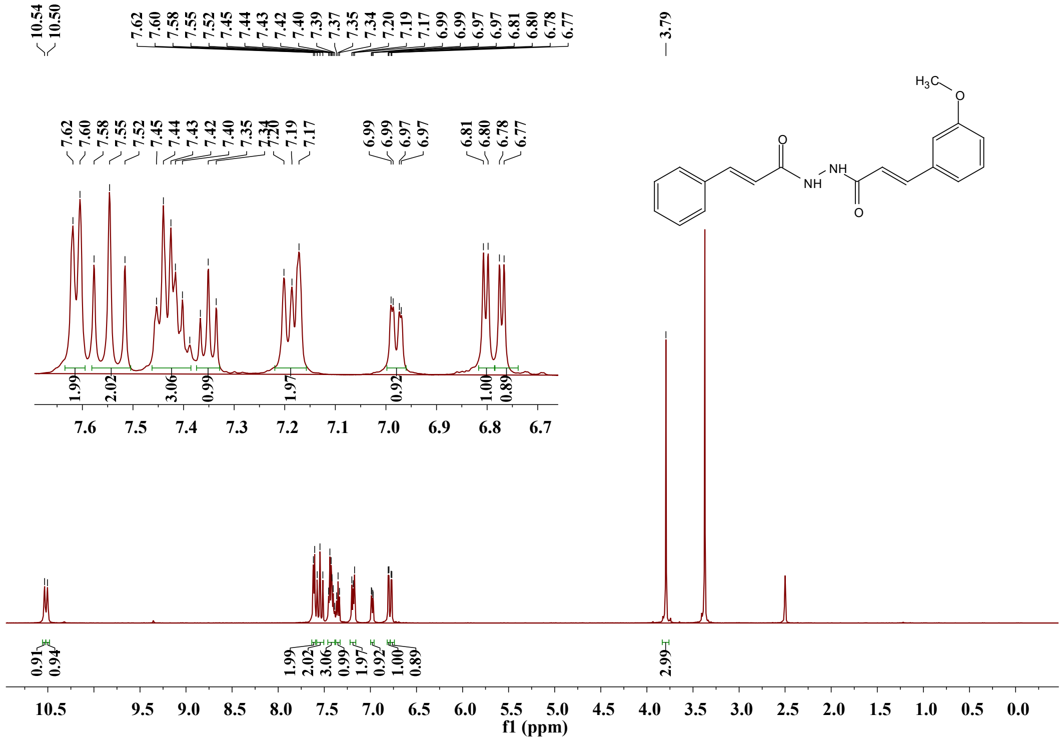

Figure S11. ^{1}H NMR Spectrum (DMSO-d_6, 500 MHz) of I_6.

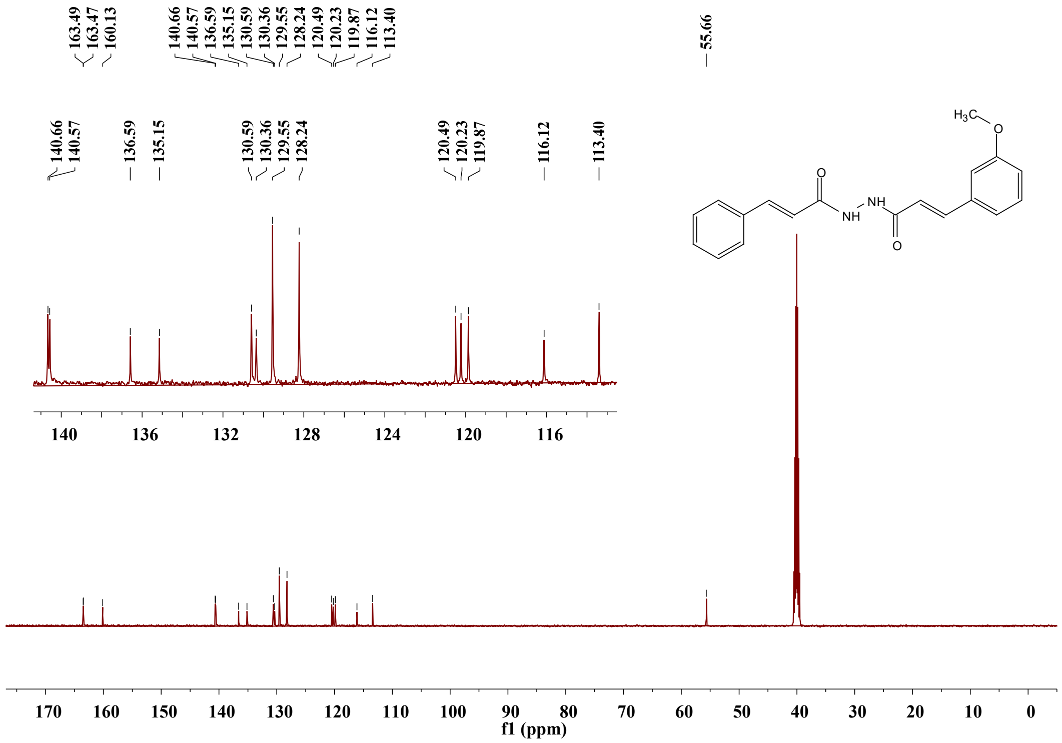

Figure S12. ^{13}C NMR Spectrum (DMSO-d_6, 126 MHz) of I_6.

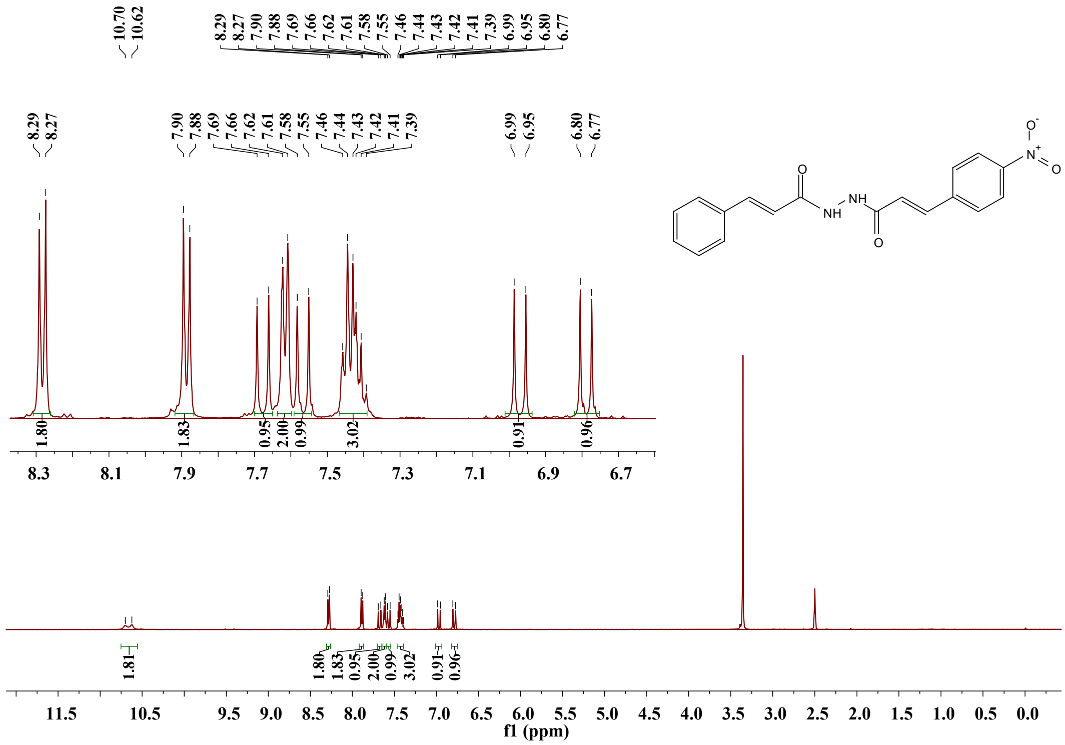

Figure S13. ^{1}H NMR Spectrum (DMSO-d_6, 500 MHz) of I_7.

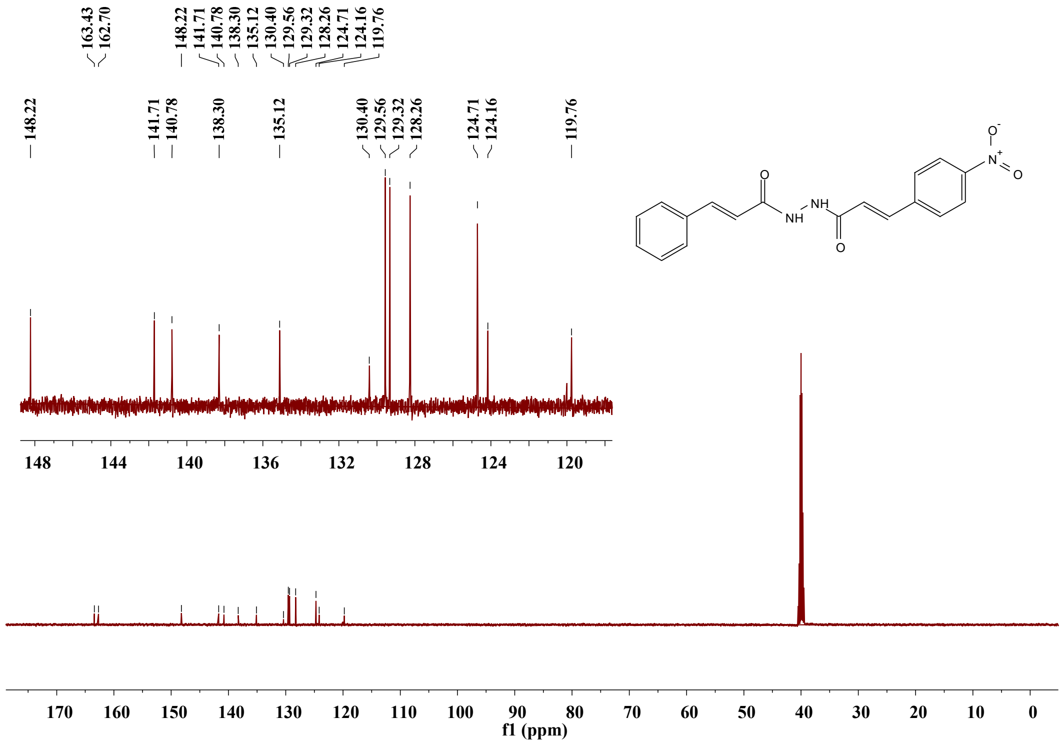

Figure S14. ^{13}C NMR Spectrum (DMSO-d_6, 126 MHz) of I_7.

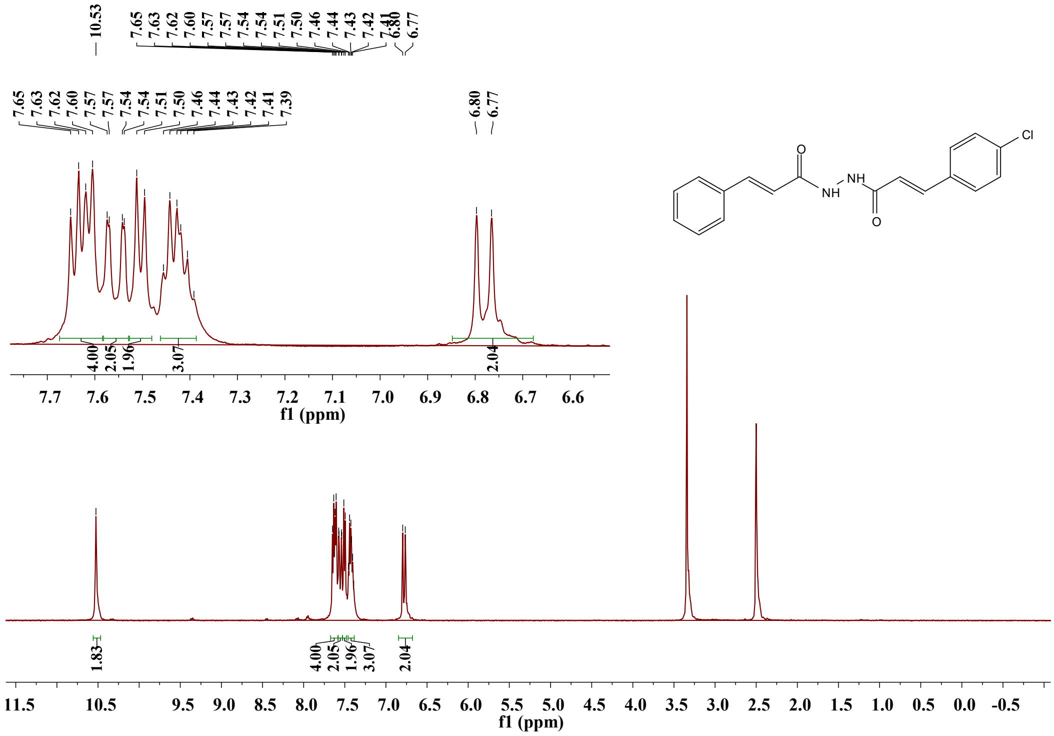

Figure S15. ^{1}H NMR Spectrum (DMSO-d_6, 500 MHz) of I_8.

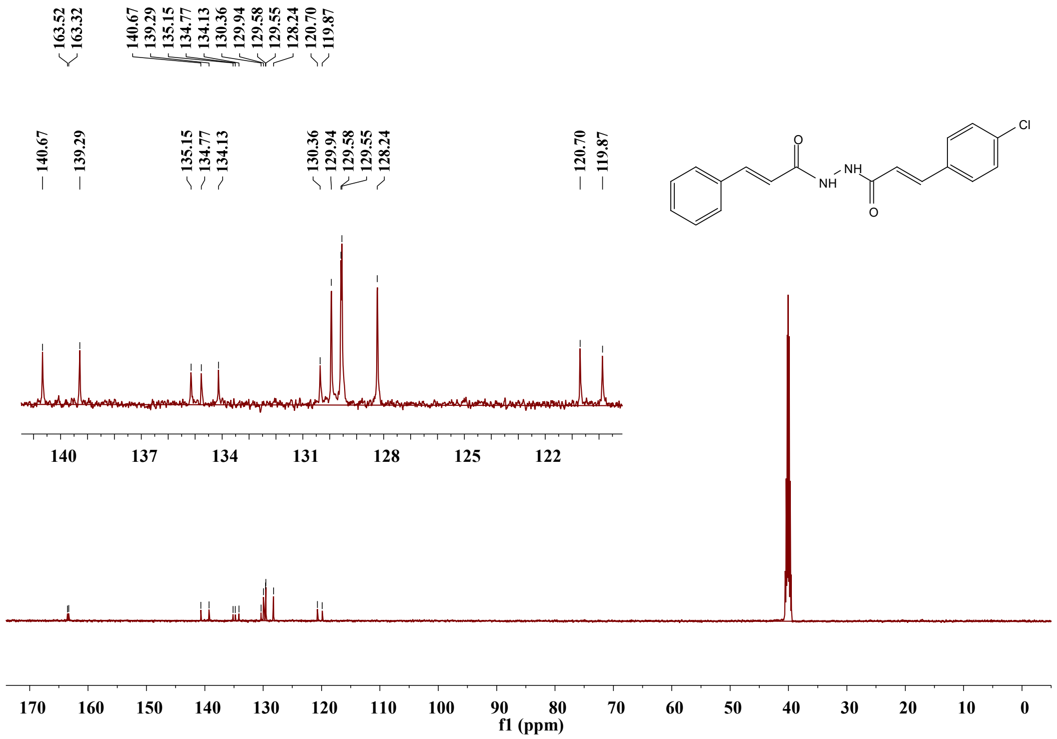

Figure S16. ^{13}C NMR Spectrum (DMSO-d_6, 126 MHz) of I_8.

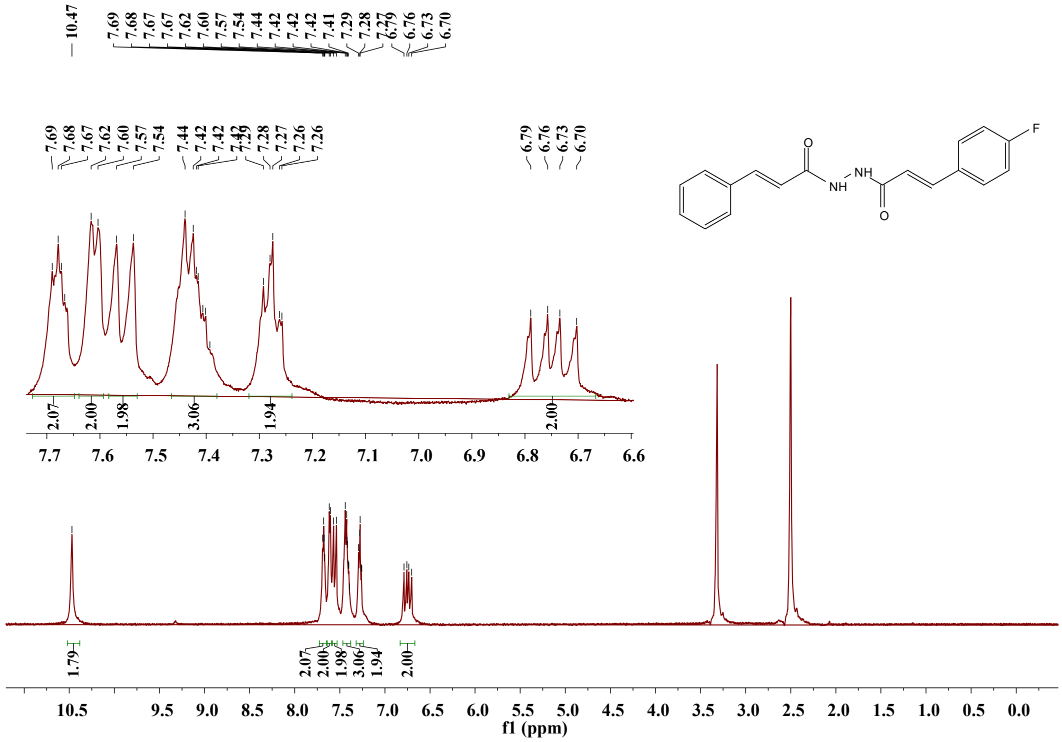

Figure S17. ^{1}H NMR Spectrum (DMSO-d_6, 500 MHz) of I_9.

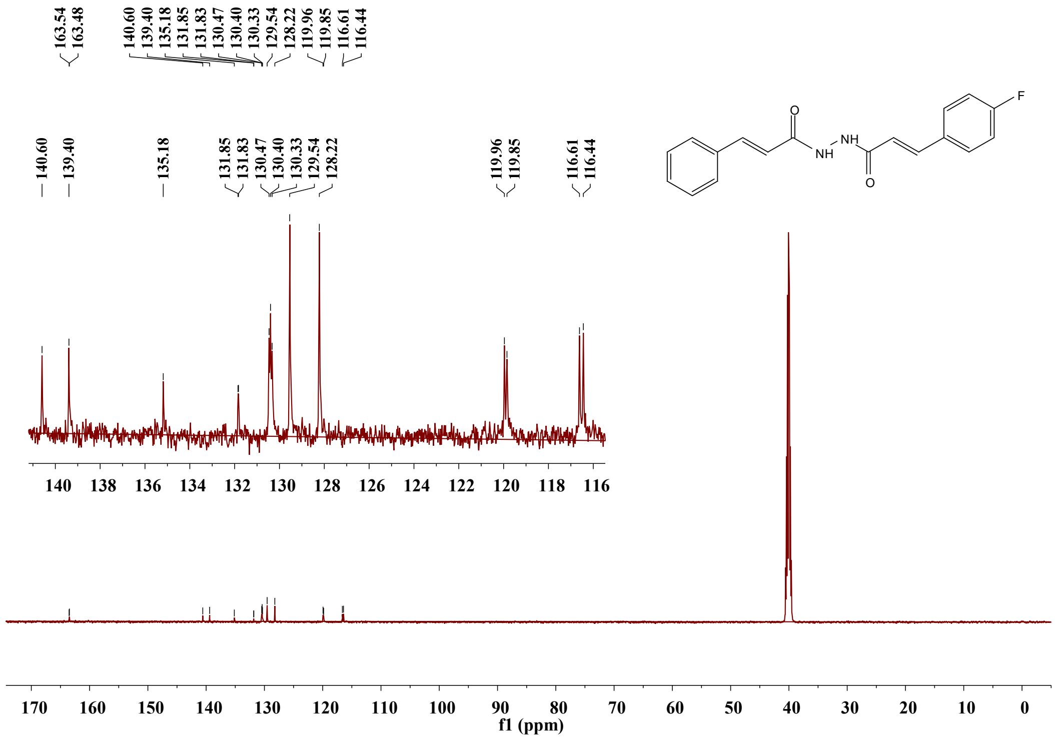

Figure S18. ^{13}C NMR Spectrum (DMSO-d_6, 126 MHz) of I$_9$.

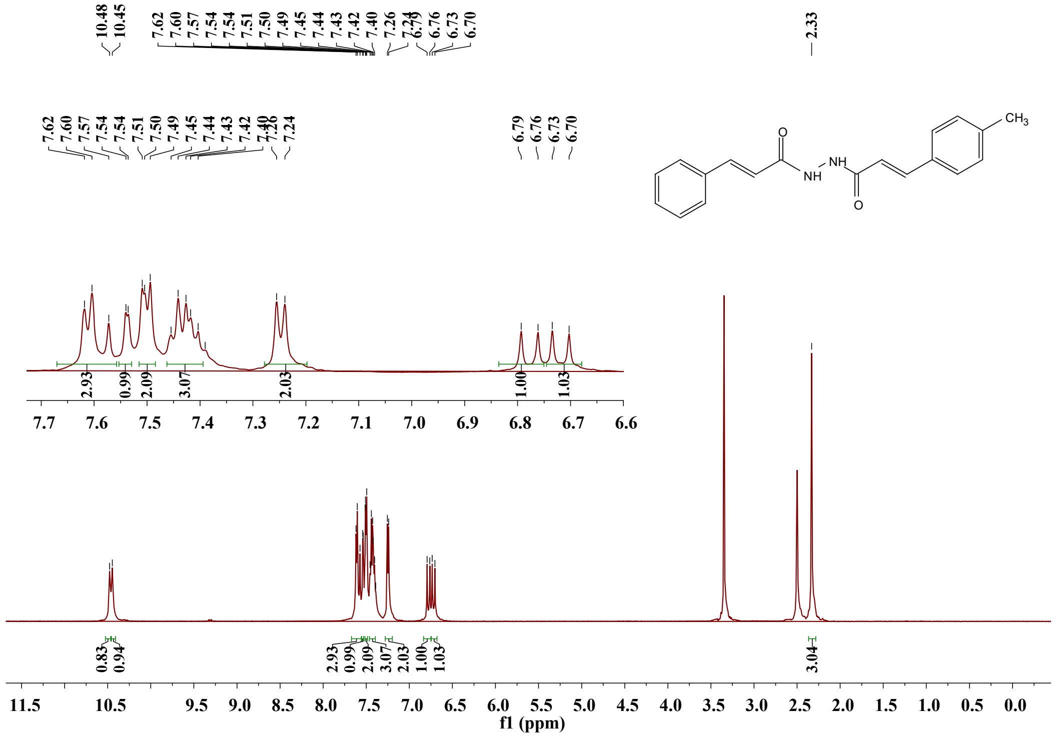

Figure S19. ^{1}H NMR Spectrum (DMSO-d_6, 500 MHz) of I$_{10}$.

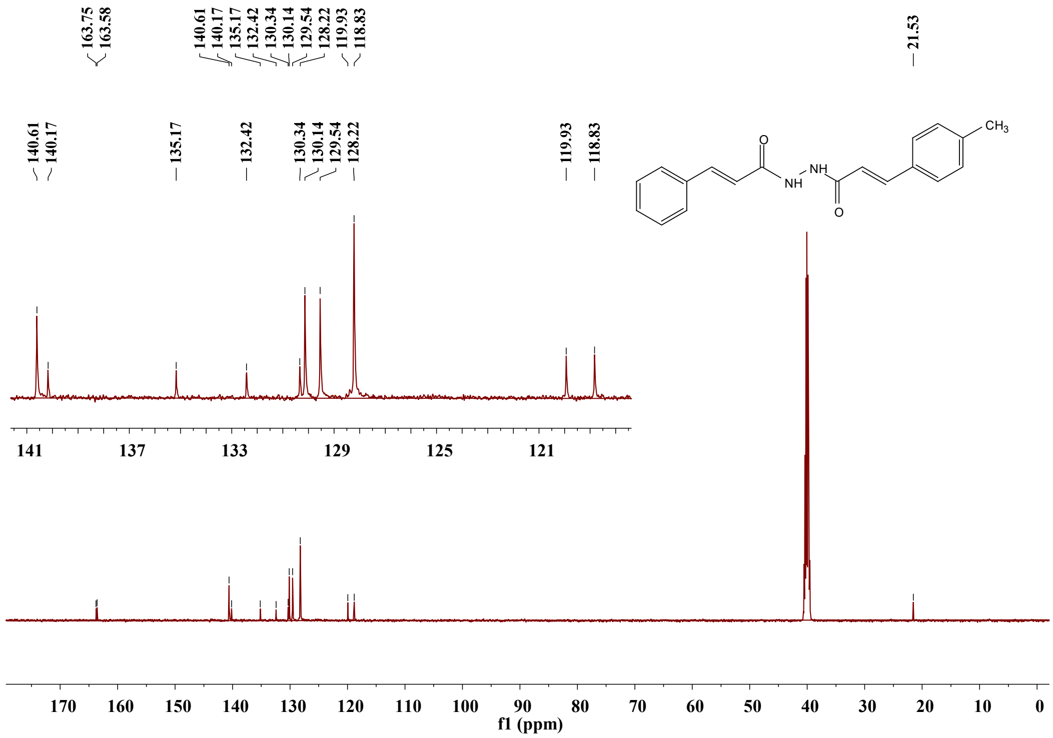

Figure S20. ^{13}C NMR Spectrum (DMSO-d_6, 126 MHz) of I_{10}.

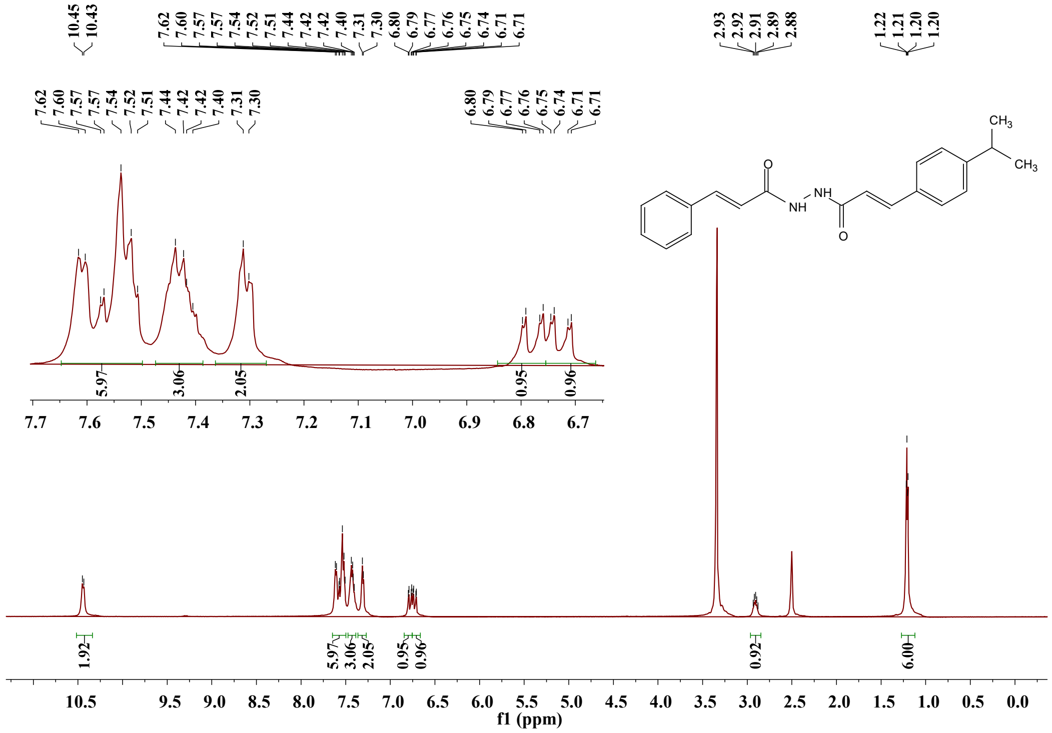

Figure S21. ^{1}H NMR Spectrum (DMSO-d_6, 500 MHz) of I_{11}.

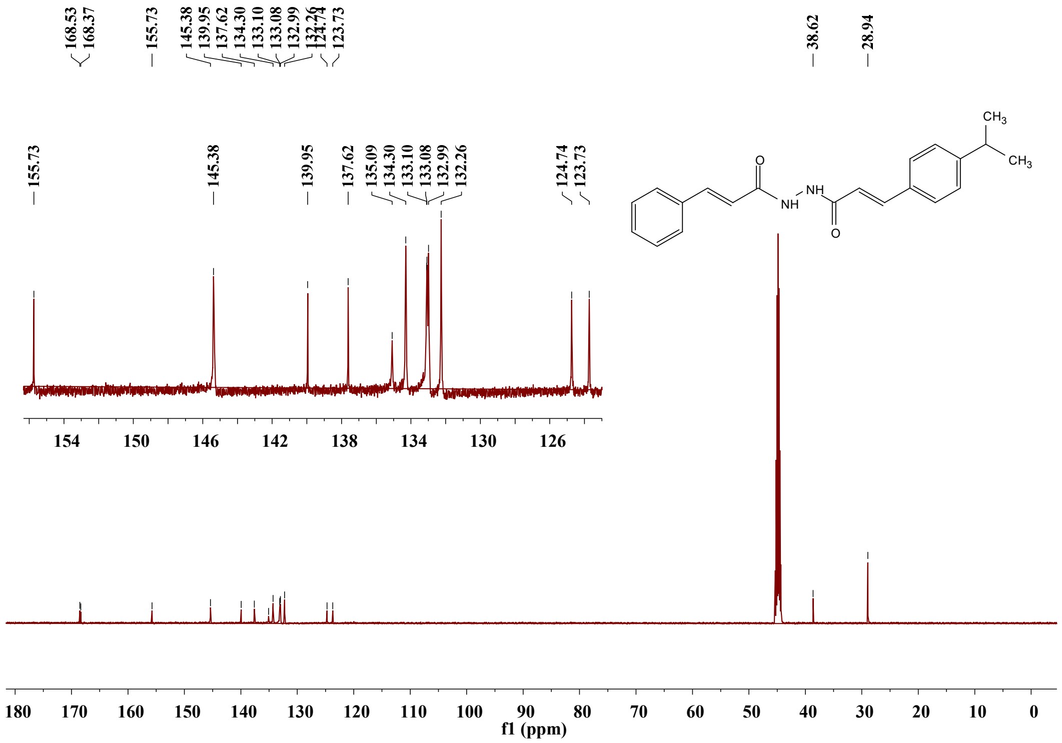

Figure S22. ^{13}C NMR Spectrum (DMSO-d_6, 126 MHz) of I_{11}.

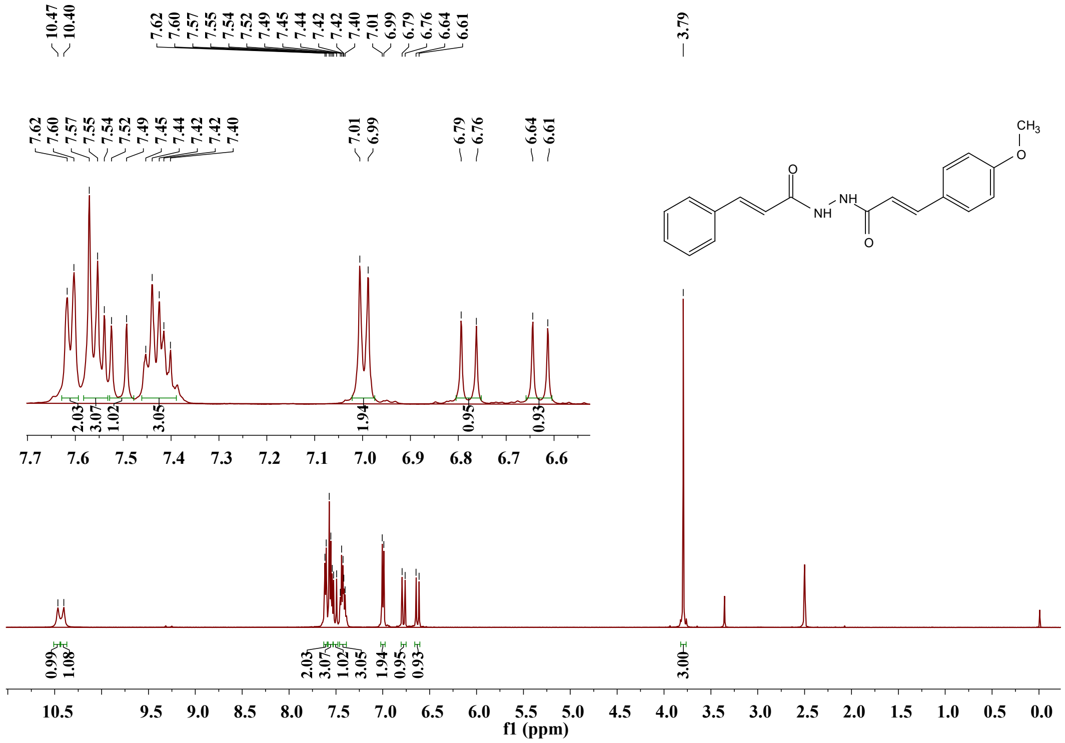

Figure S23. ^{1}H NMR Spectrum (DMSO-d_6, 500 MHz) of I_{12}.

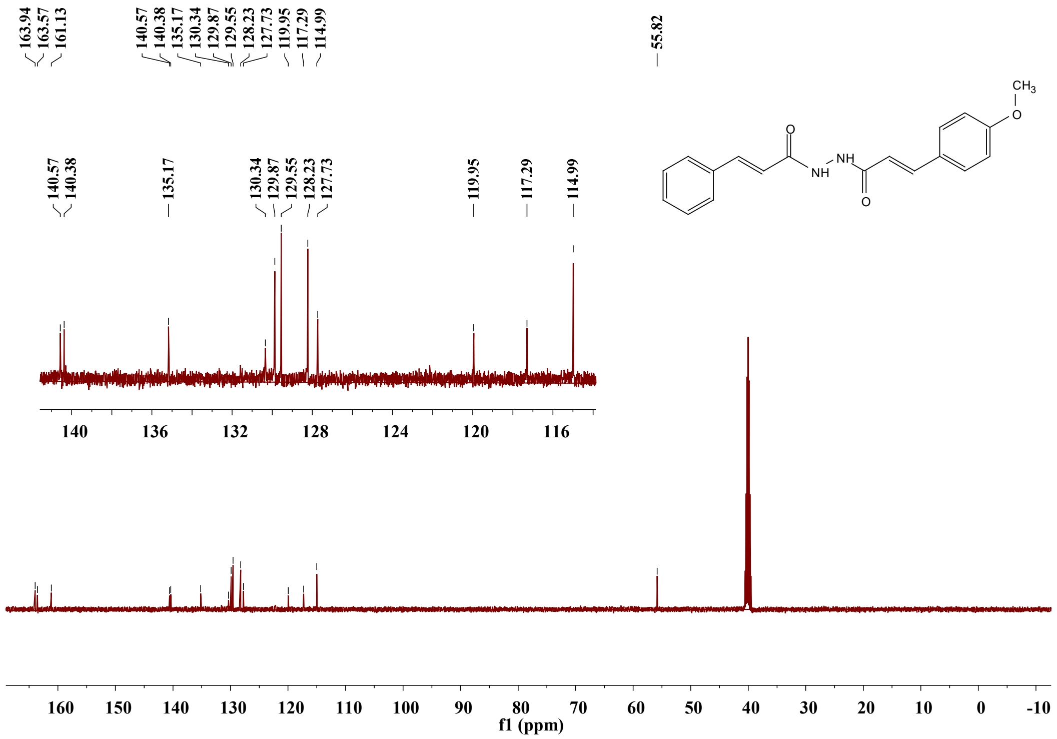

Figure S24. ^{13}C NMR Spectrum (DMSO-d_6, 126 MHz) of I_{12}.

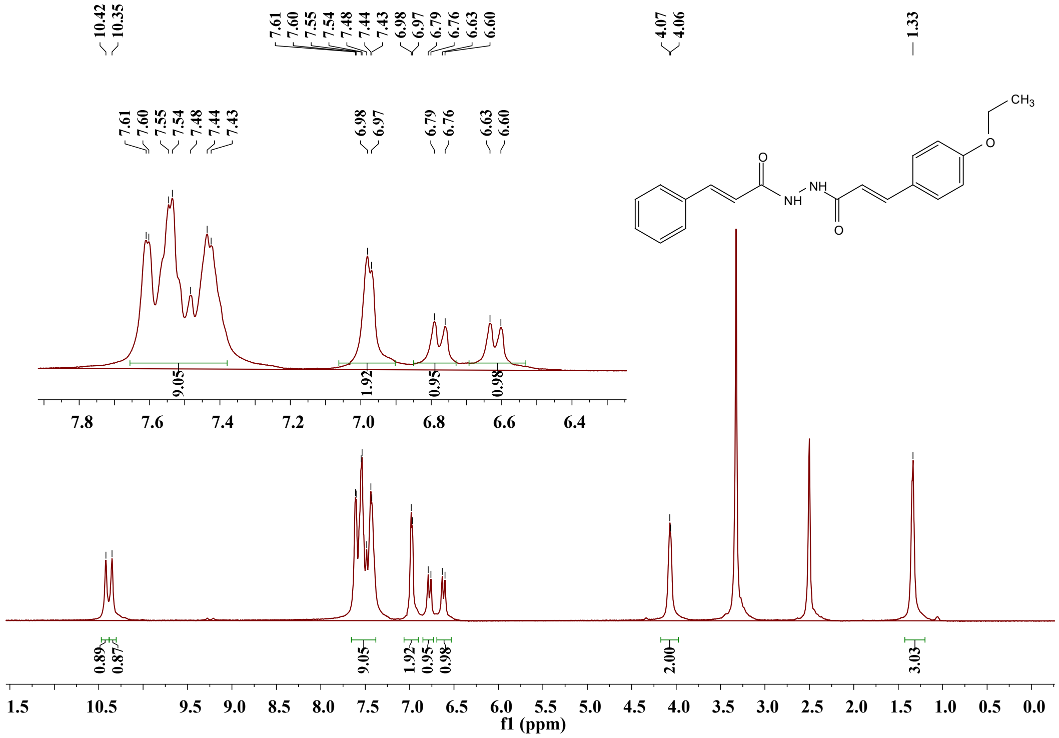

Figure S25. ^{1}H NMR Spectrum (DMSO-d_6, 500 MHz) of I_{13}.

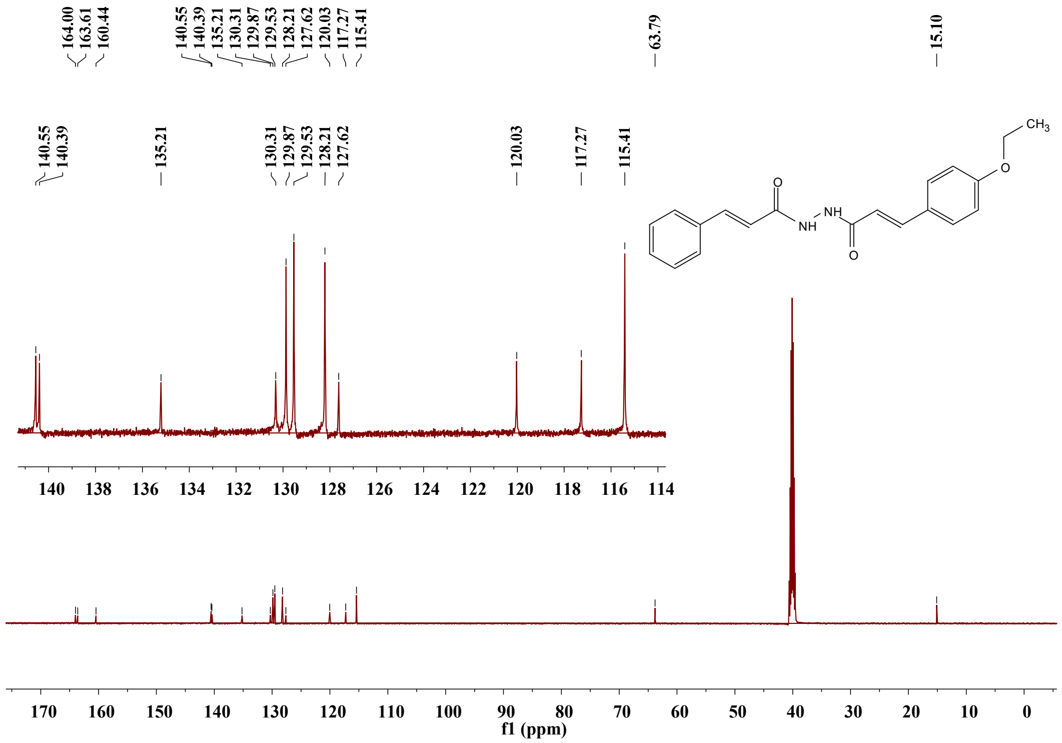

Figure S26. ^{13}C NMR Spectrum (DMSO-d_6, 126 MHz) of I_{13}.

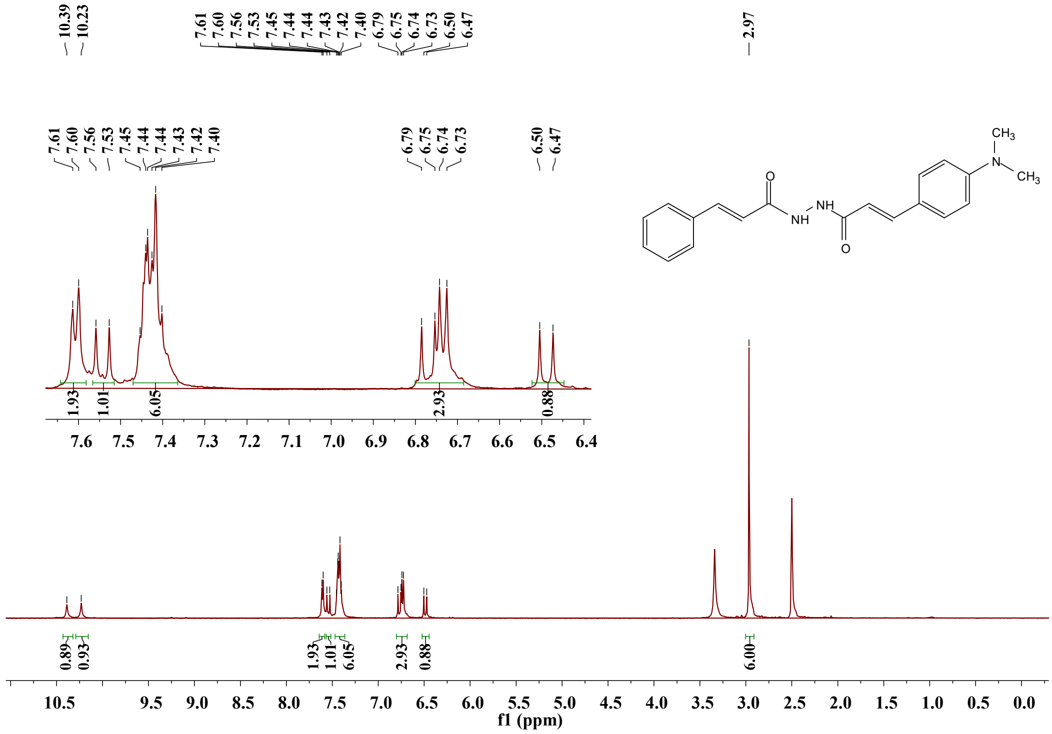

Figure S27. ^{1}H NMR Spectrum (DMSO-d_6, 500 MHz) of I_{14}.

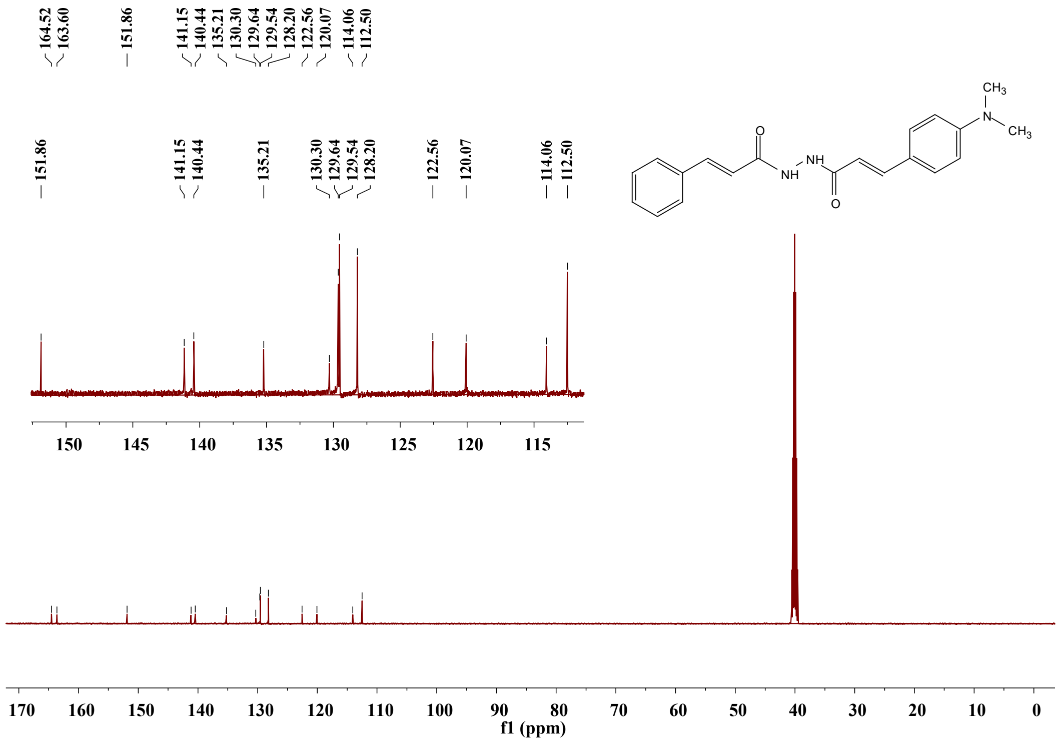

Figure S28. ^{13}C NMR Spectrum (DMSO-d_6, 126 MHz) of I_{14}.

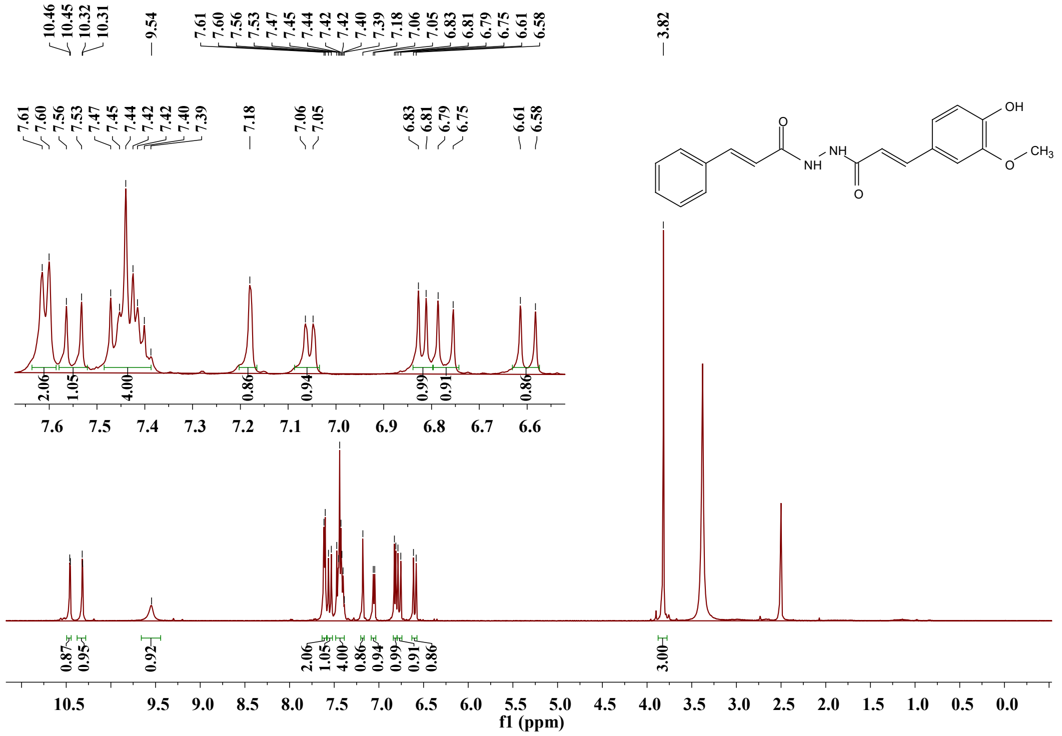

Figure S29. ^{1}H NMR Spectrum (DMSO-d_6, 500 MHz) of I_{15}.

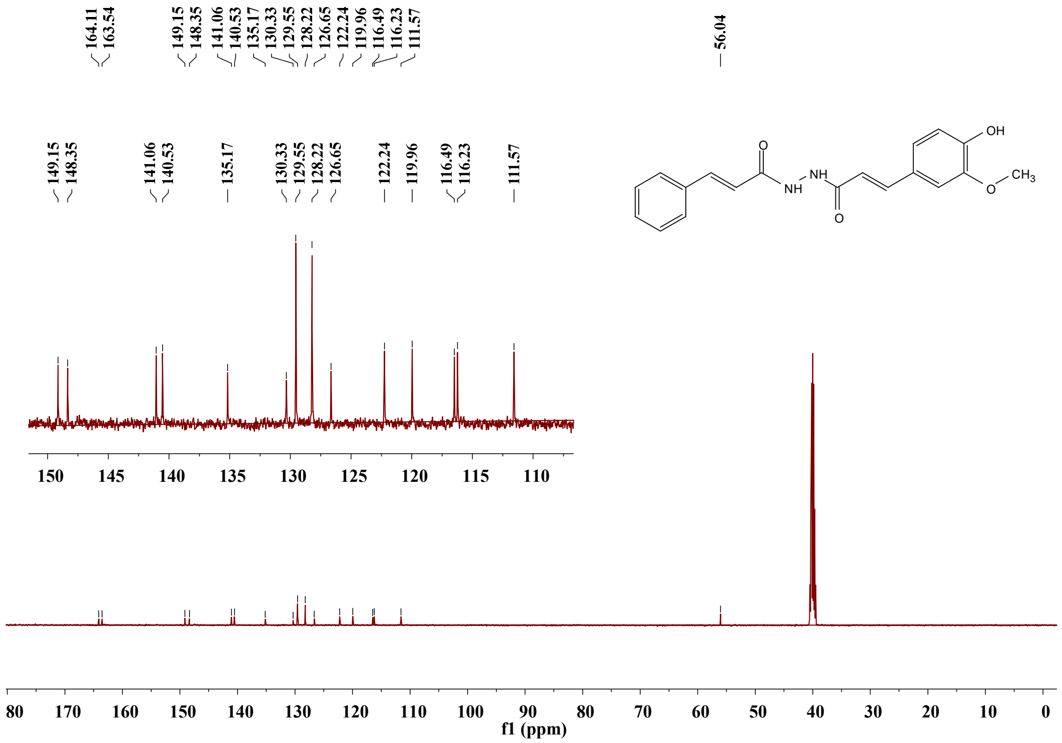

Figure S30. ^{13}C **NMR Spectrum (DMSO-*d*$_6$, 126 MHz) of I$_{15}$.**

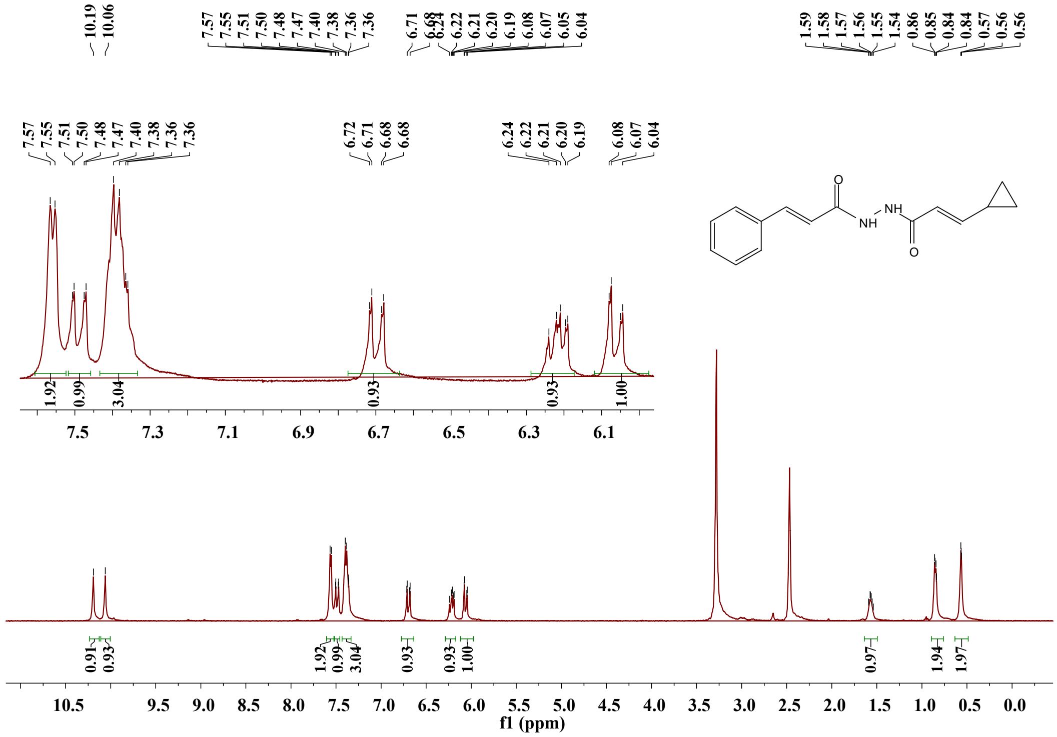

Figure S31. ^{1}H **NMR Spectrum (DMSO-*d*$_6$, 500 MHz) of I$_{16}$.**

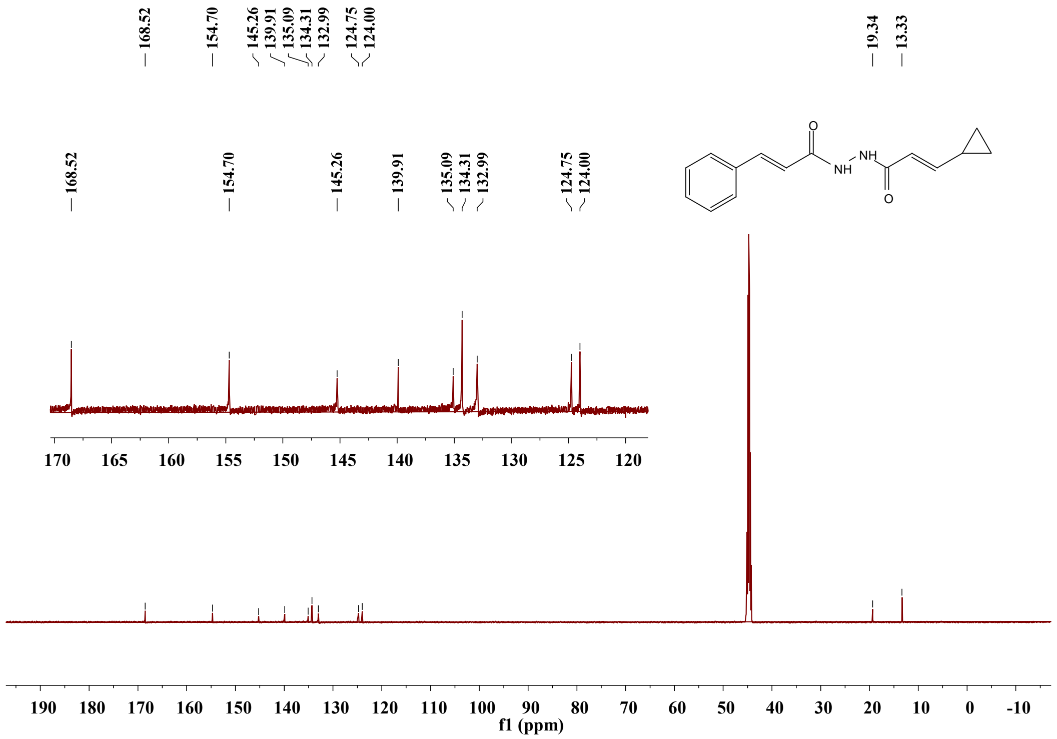

Figure S32. ^{13}C NMR Spectrum (DMSO-d_6, 126 MHz) of I_{16}.

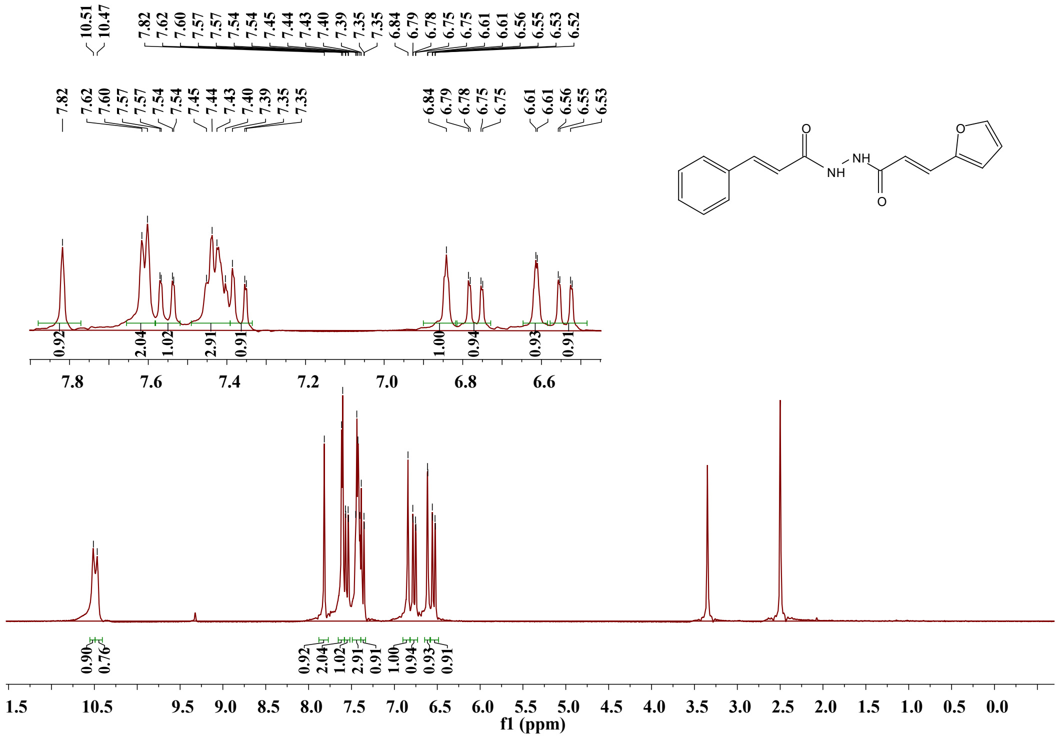

Figure S33. ^{1}H NMR Spectrum (DMSO-d_6, 500 MHz) of I_{17}.

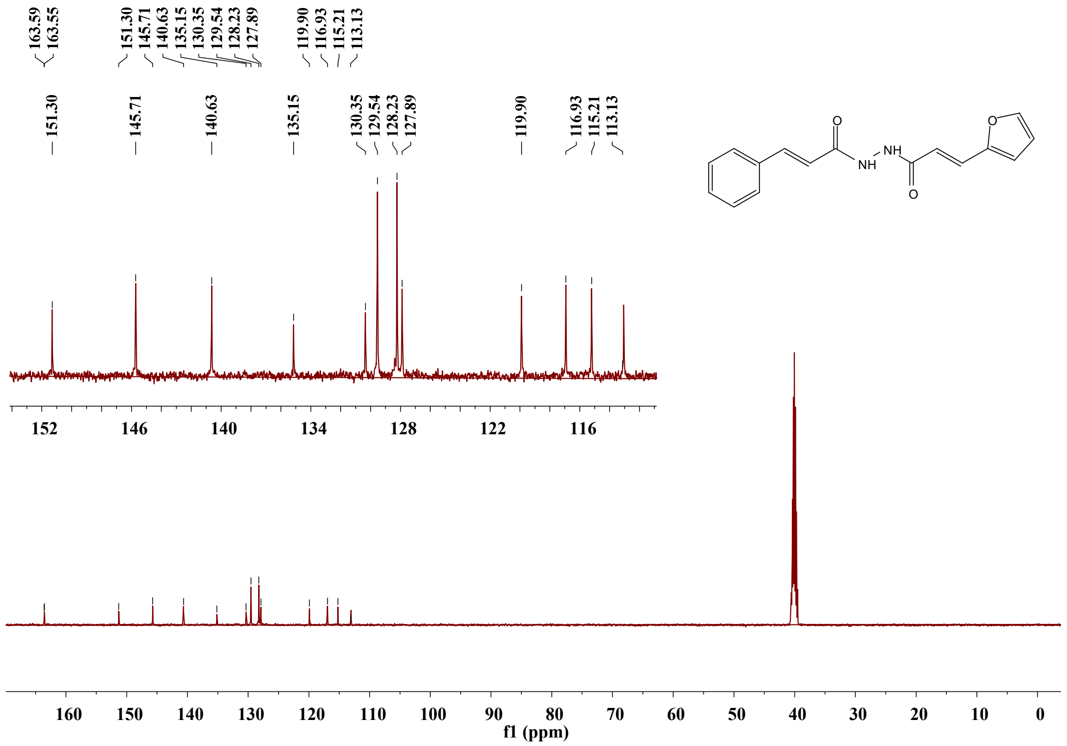

Figure S34. ^{13}C NMR Spectrum (DMSO-d_6, 126 MHz) of I_{17}.

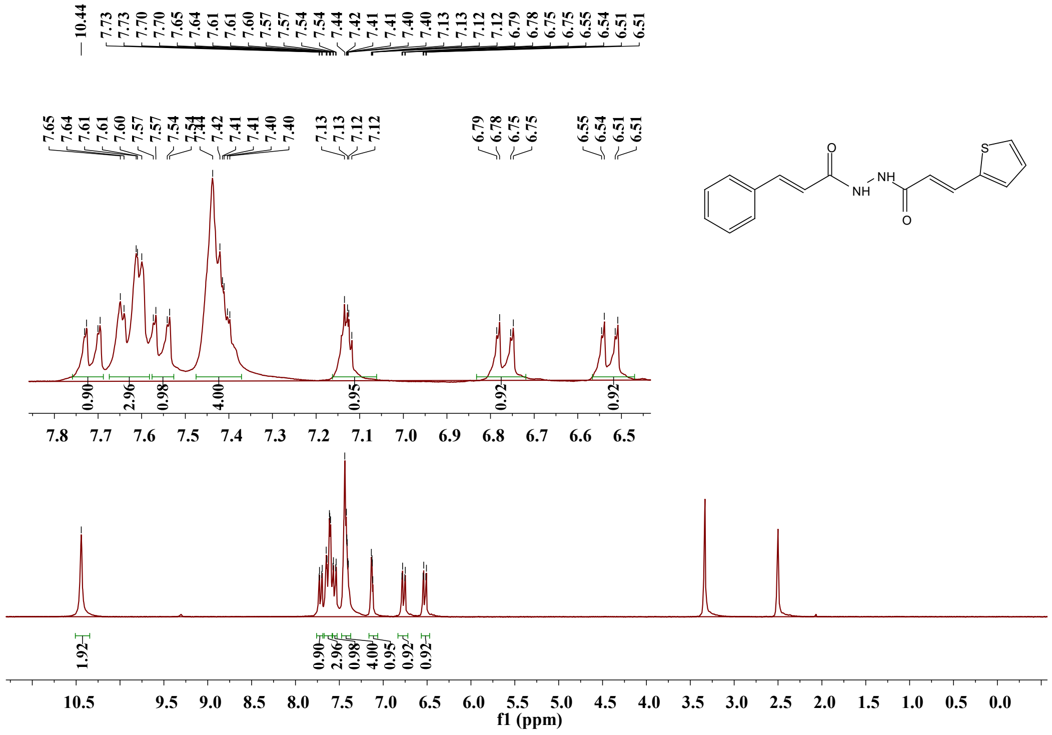

Figure S35. ^{1}H NMR Spectrum (DMSO-d_6, 500 MHz) of I_{18}.

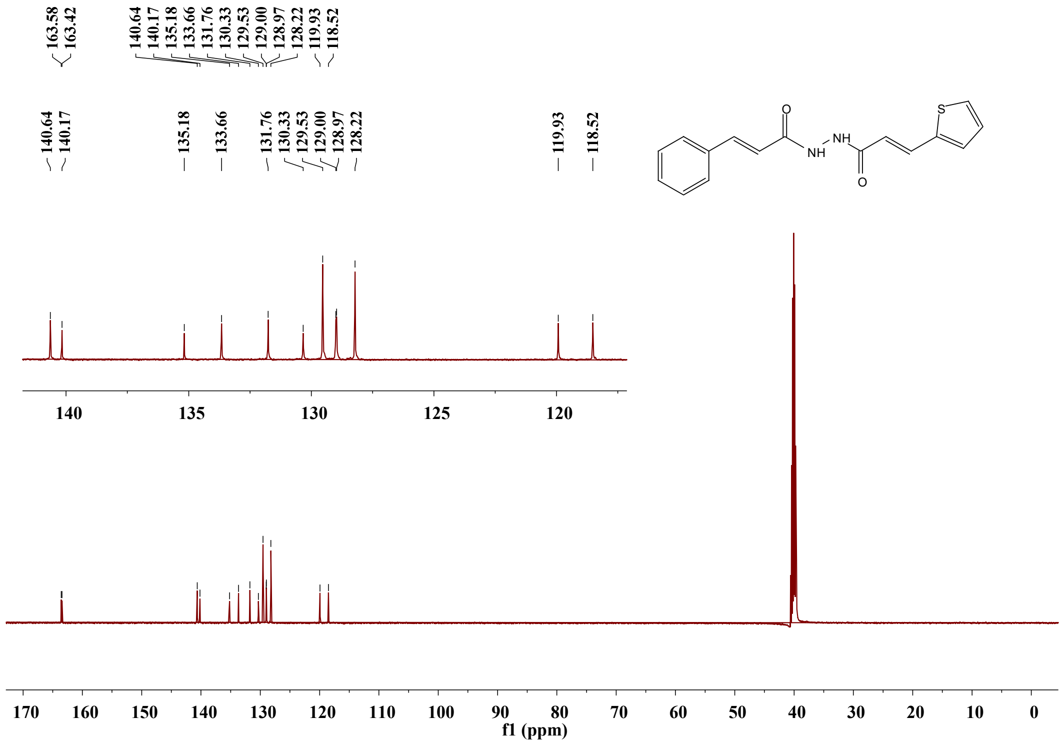

Figure S36. ^{13}C NMR Spectrum (DMSO-d_6, 126 MHz) of I_{18}.

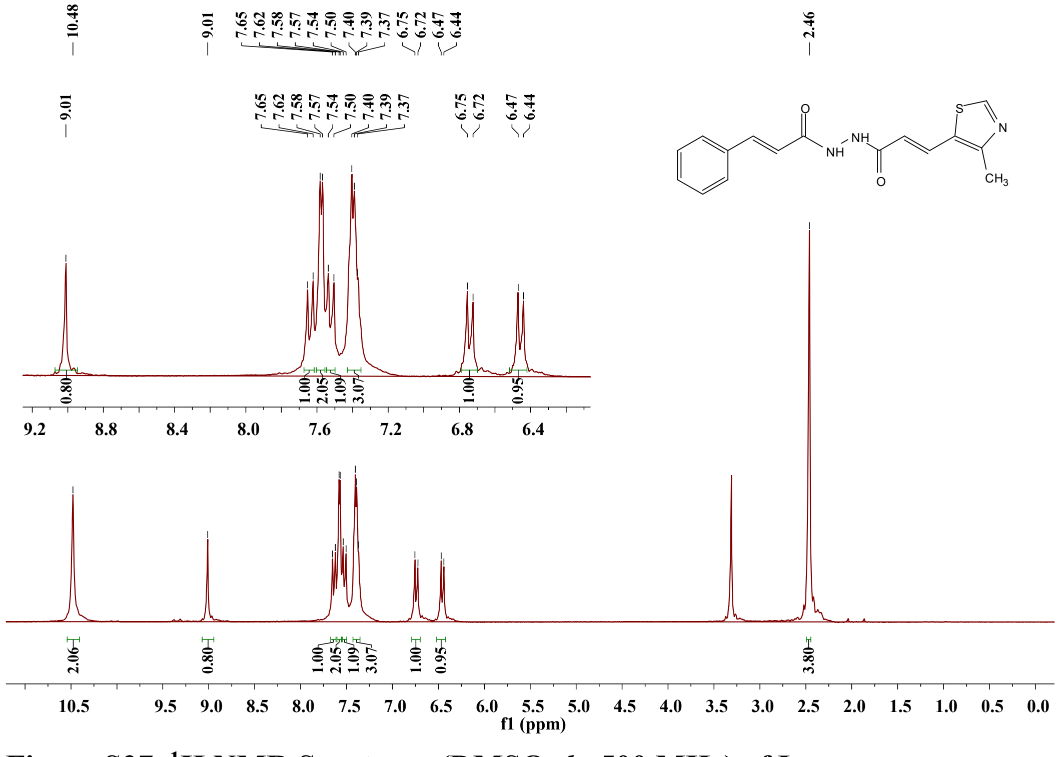

Figure S37. ^{1}H NMR Spectrum (DMSO-d_6, 500 MHz) of I_{19}.

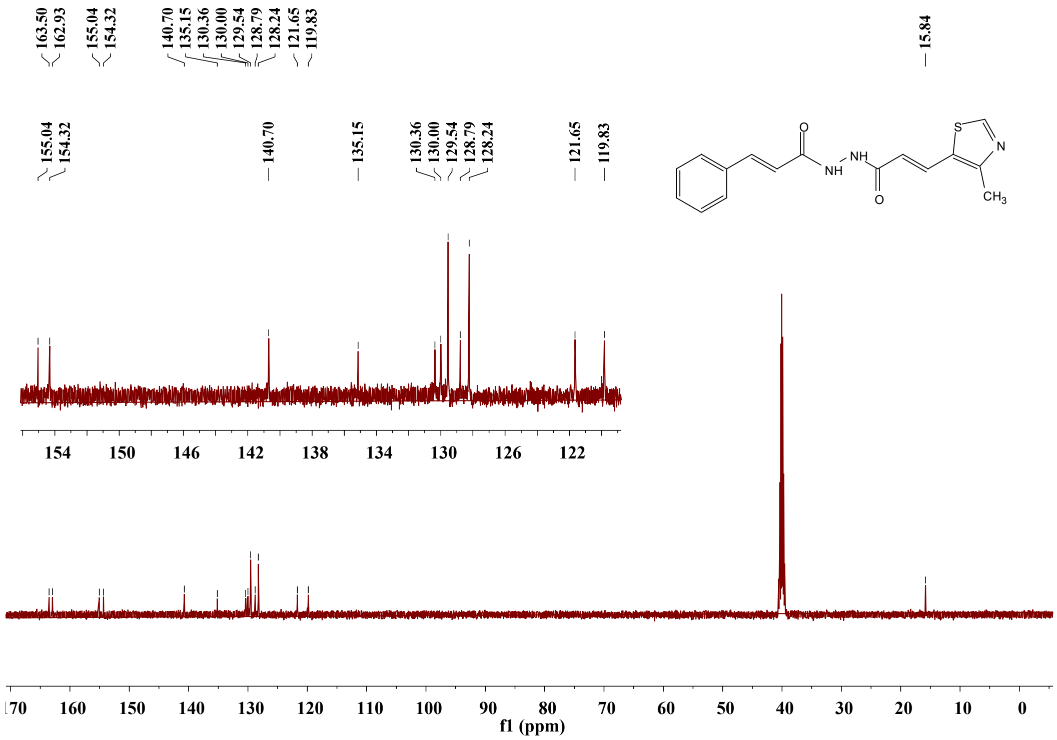

Figure S38. ^{13}C NMR Spectrum (DMSO-d_6, 126 MHz) of **I**$_{19}$.

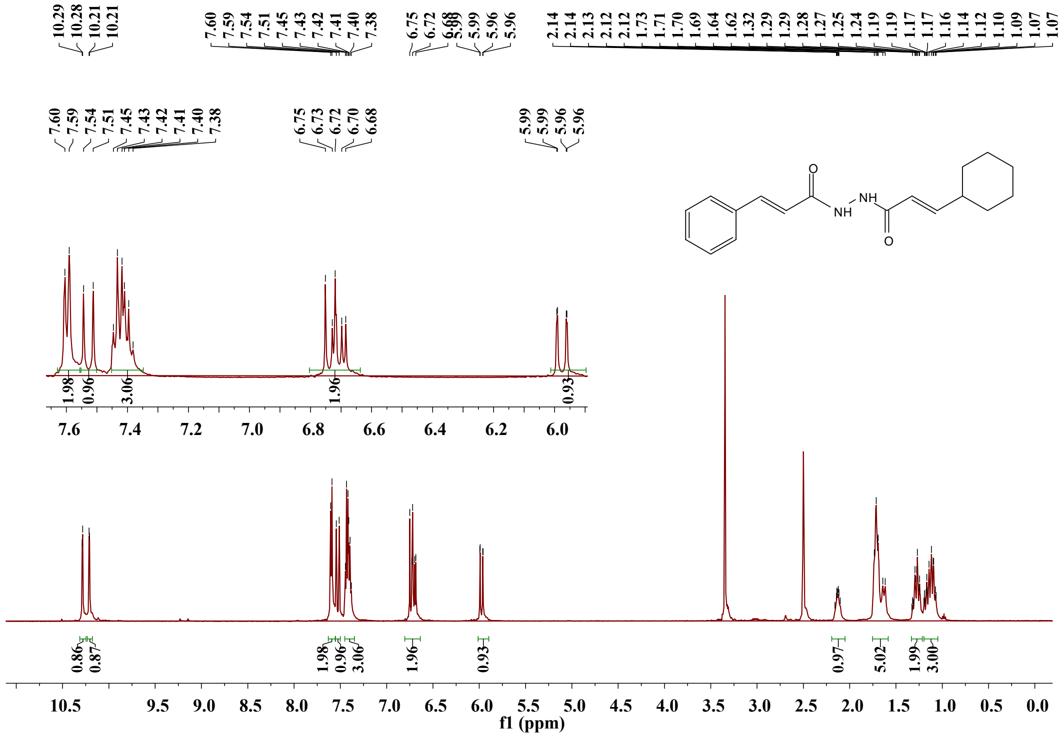

Figure S39. ^{1}H NMR Spectrum (DMSO-d_6, 500 MHz) of **I**$_{20}$.

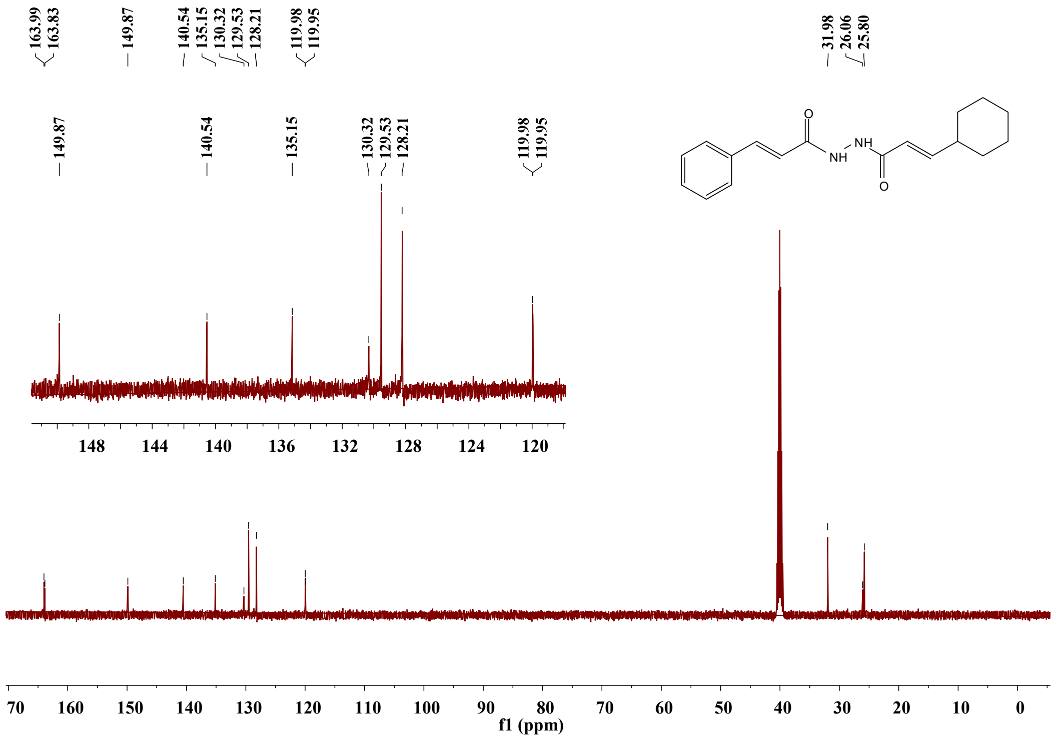

Figure S40. ^{13}**C NMR Spectrum (DMSO-*d*$_6$, 126 MHz) of I$_{20}$.**

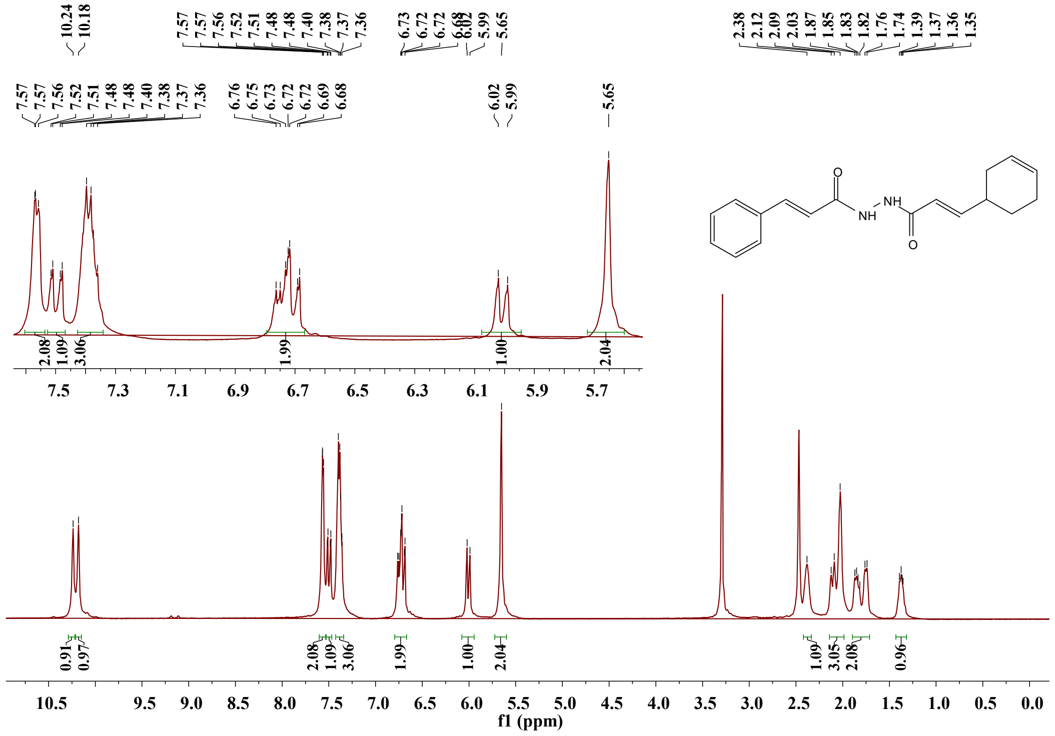

Figure S41. ^{1}**H NMR Spectrum (DMSO-*d*$_6$, 500 MHz) of I$_{21}$.**

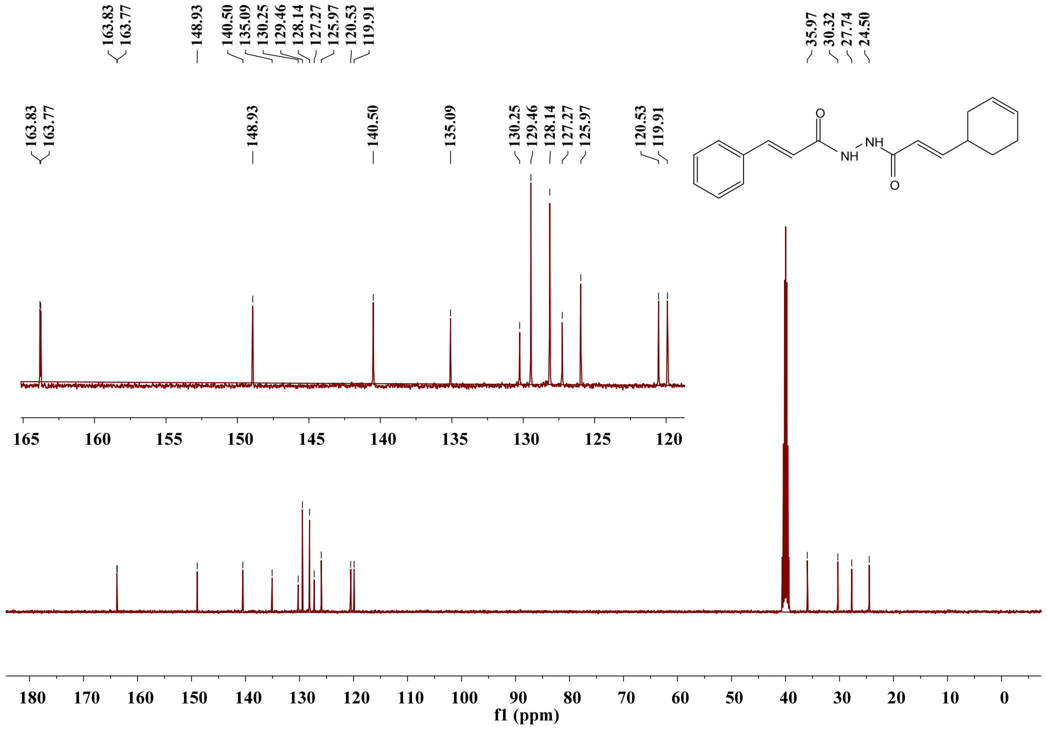

Figure S42. ^{13}**C NMR Spectrum (DMSO-*d*$_6$, 101 MHz) of I$_{21}$.**

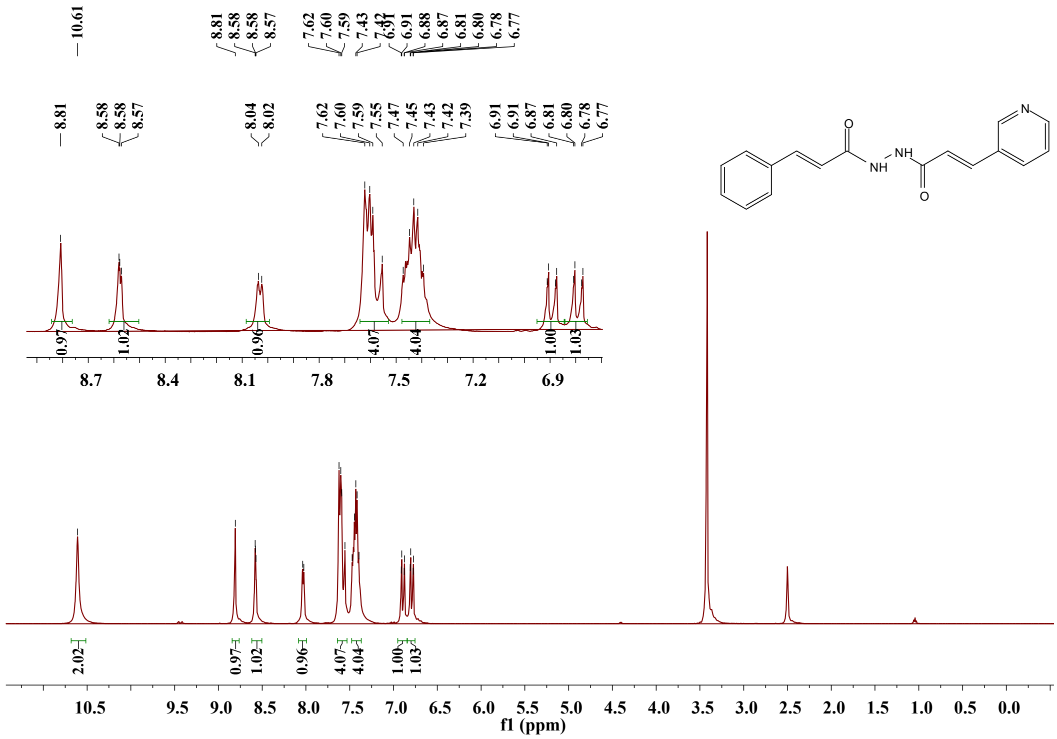

Figure S43. ^{1}**H NMR Spectrum (DMSO-*d*$_6$, 500 MHz) of I$_{22}$.**

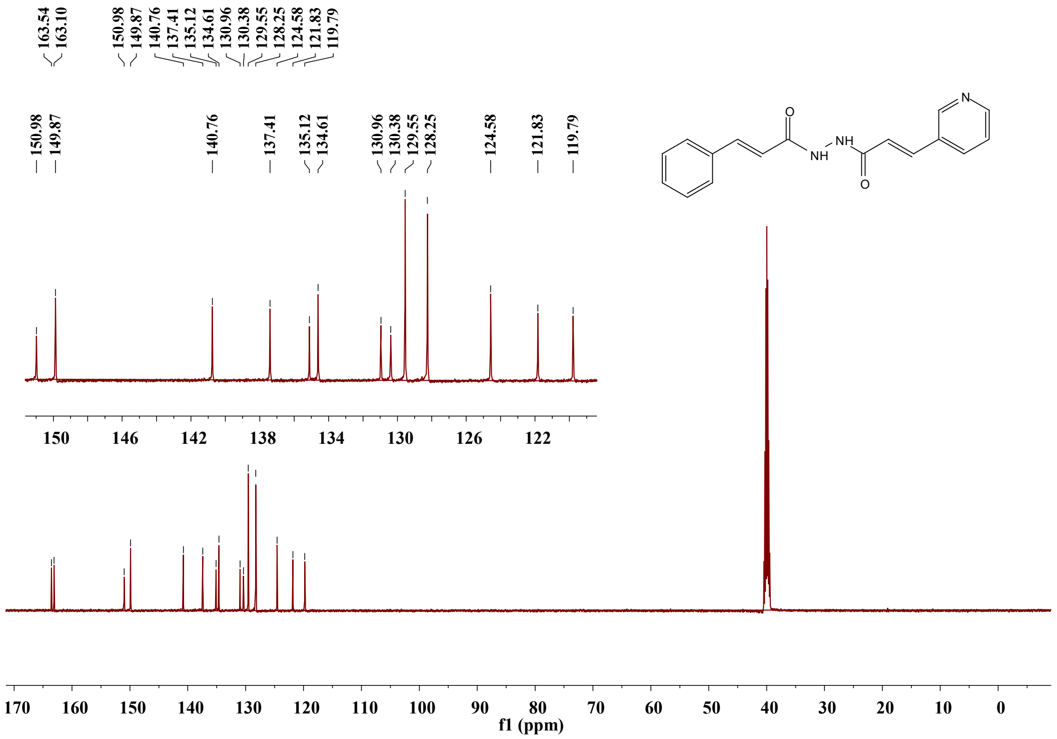

Figure S44. ^{13}C NMR Spectrum (DMSO-d_6, 126 MHz) of I_{22}.

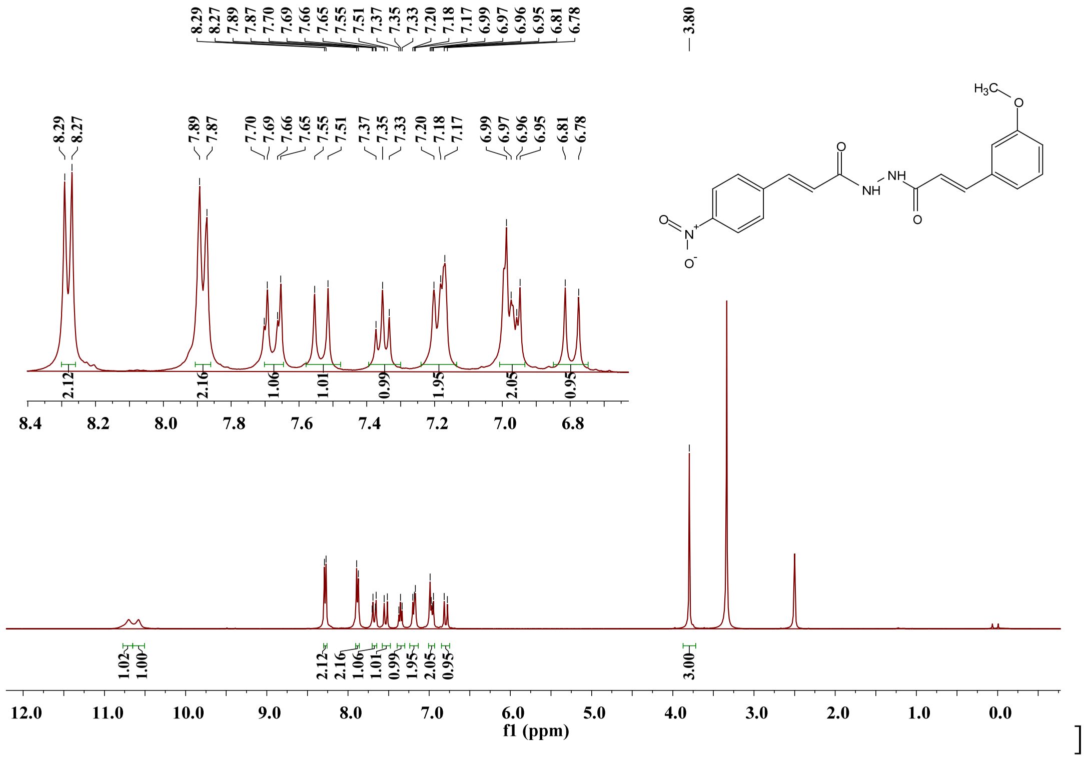

Figure S45. ^{1}H NMR Spectrum (DMSO-d_6, 400 MHz) of I_{23}.

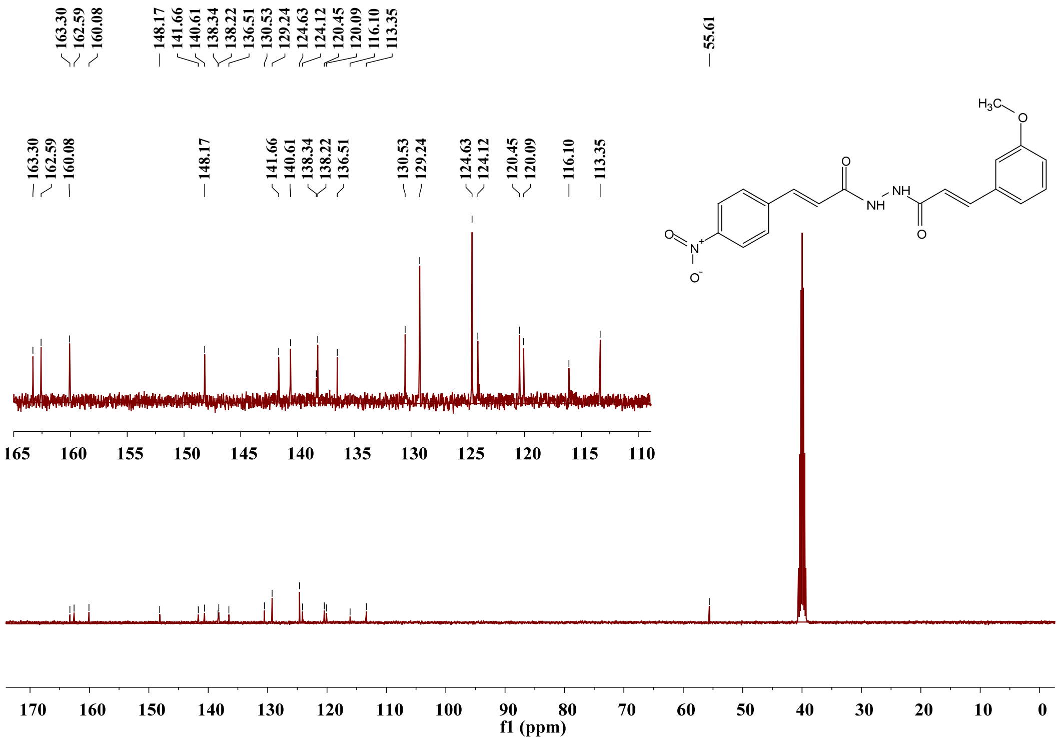

Figure S46. ^{13}C NMR Spectrum (DMSO-d_6, 101 MHz) of I_{23}.

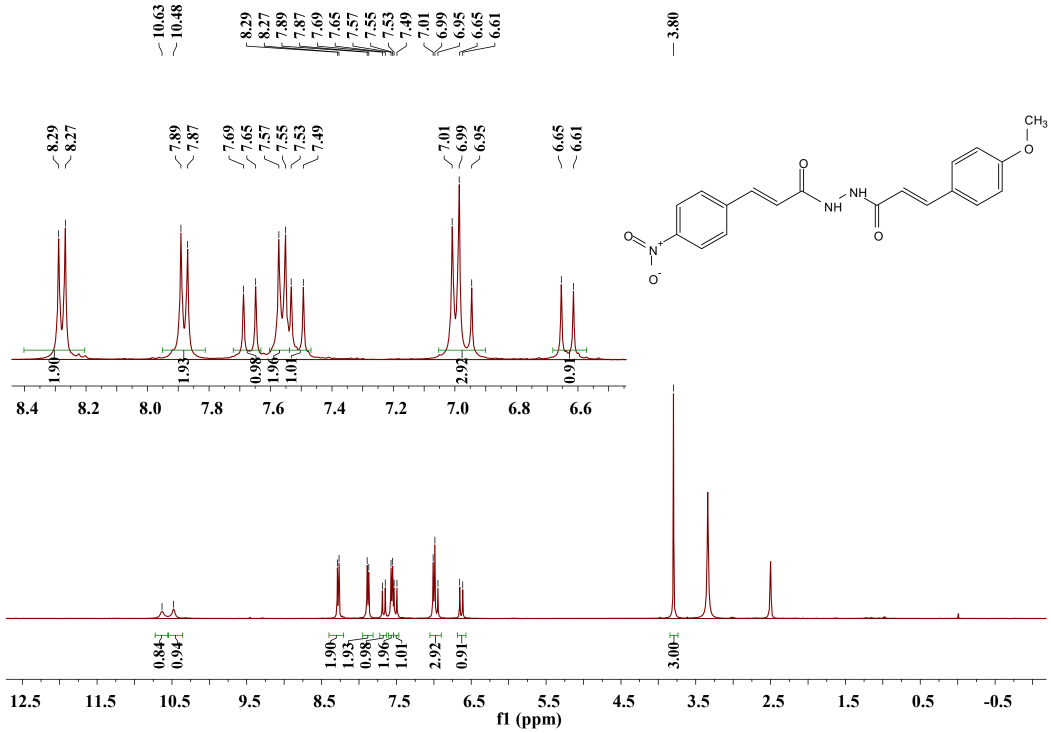

Figure S47. ^{1}H NMR Spectrum (DMSO-d_6, 400 MHz) of I_{24}.

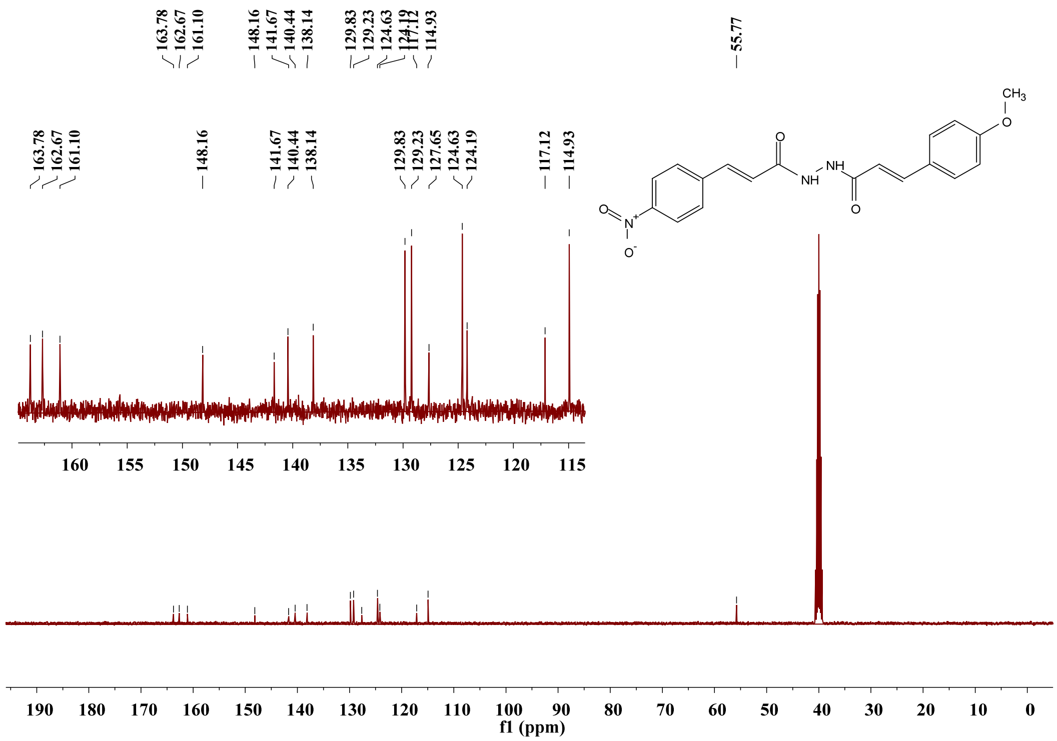

Figure S48. ^{13}C NMR Spectrum (DMSO-d_6, 101 MHz) of I_{24}.

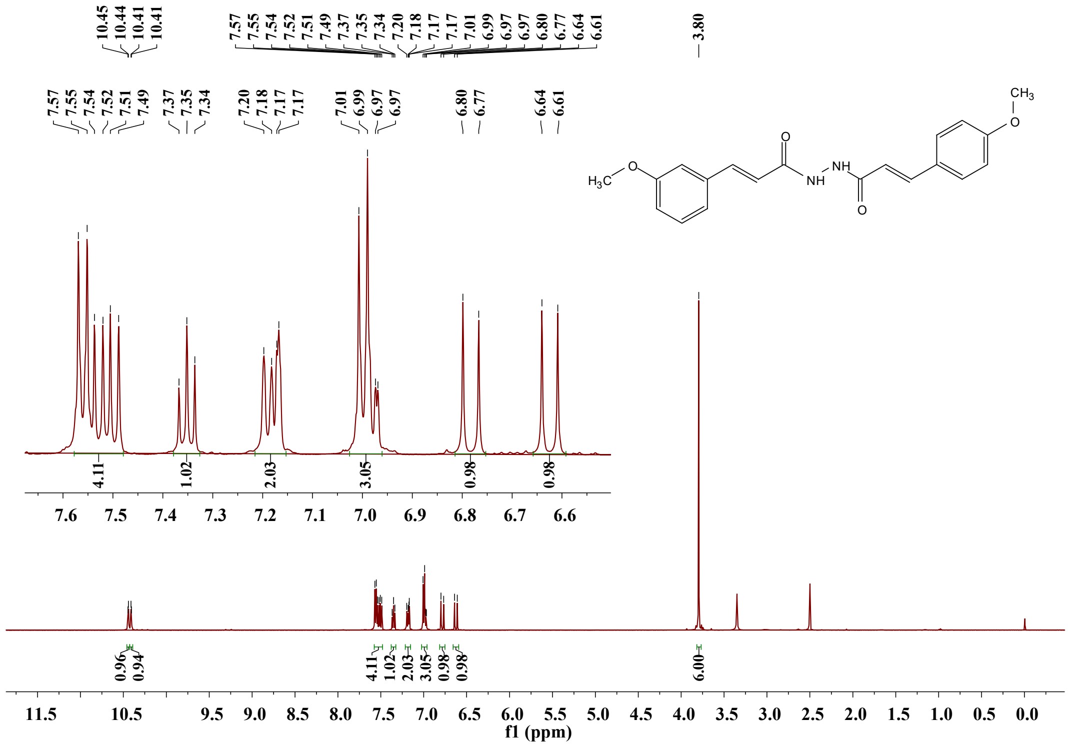

Figure S49. ^{1}H NMR Spectrum (DMSO-d_6, 500 MHz) of I_{25}.

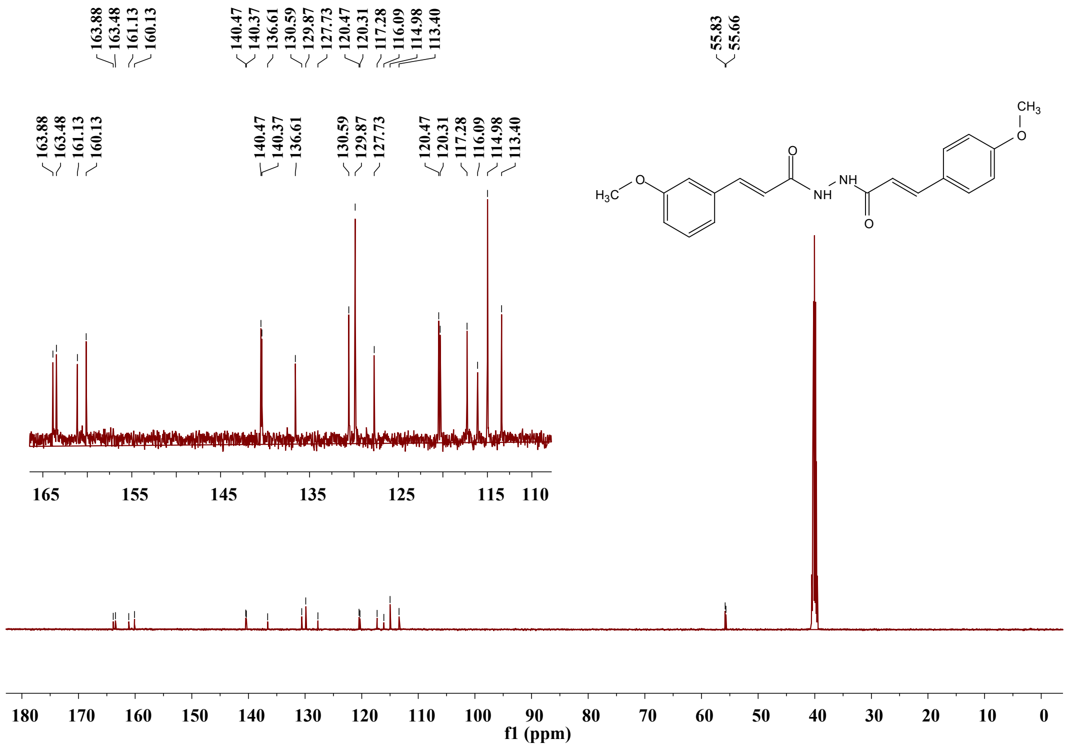

Figure S50. ^{13}C NMR Spectrum (DMSO-d_6, 126 MHz) of I_{25}.

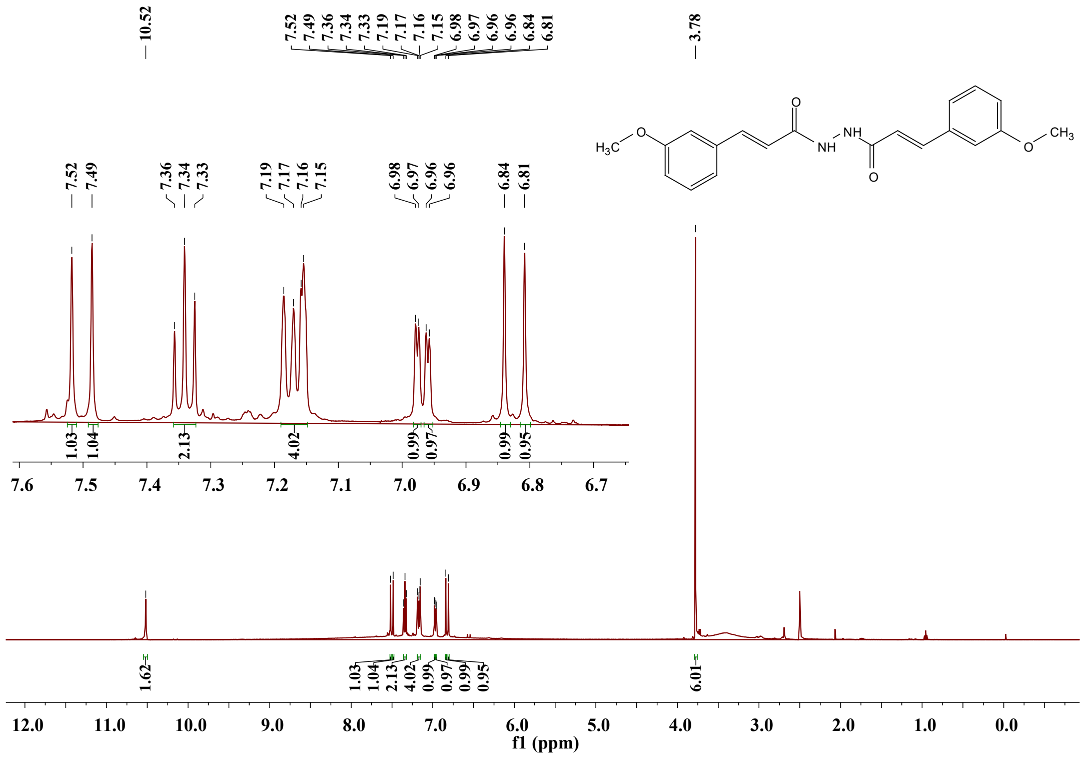

Figure S51. ^{1}H NMR Spectrum (DMSO-d_6, 500 MHz) of I_{26}.

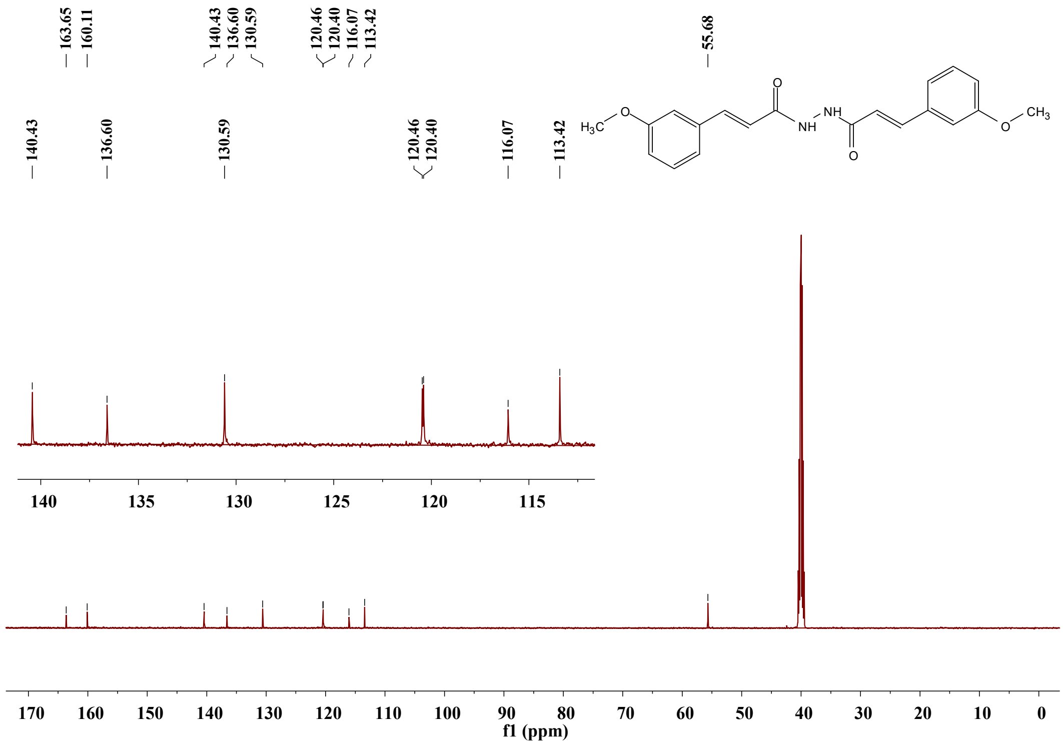

Figure S52. ^{13}**C NMR Spectrum (DMSO-*d*$_6$, 126 MHz) of I**$_{26}$**.**

References

1. X. N. Zhang, M. Breslav, J. Grimm, K. L. Guan, A. H. Huang, F. Q. Liu, C. A. Maryanoff, D. Palmer, M. Patel, Y. Qian, C. Shaw, K. Sorgi, S. Stefanick, D. W. Xu, A new procedure for preparation of carboxylic acid hydrazides, J. Org. Chem. 67 (2002) 9471-9474.
2. X. Zhou, Y. M. Feng, P. Y. Qi, W. B. Shao, Z. B. Wu, L. W. Liu, Y. Wang, H. D. Ma, P. Y. Wang, S. Yang, Synthesis and docking study of *N*-(Cinnamoyl)-*N'*-(substituted)acryloyl hydrazide derivatives containing pyridinium moieties as a novel class of filamentous temperature-sensitive protein Z inhibitors against the intractable *Xanthomonas oryzae pv. oryzae* infections in rice, J. Agric. Food Chem. 68 (2020) 8132-8142.
